# Furan- and Thiophene-2-Carbonyl Amino Acid Derivatives Activate Hypoxia-Inducible Factor via Inhibition of Factor Inhibiting Hypoxia-Inducible Factor-1

**DOI:** 10.3390/molecules23040885

**Published:** 2018-04-11

**Authors:** Shin-ichi Kawaguchi, Yuhei Gonda, Takuya Yamamoto, Yuki Sato, Hiroyuki Shinohara, Yohsuke Kobiki, Atsuhiko Ichimura, Takashi Dan, Motohiro Sonoda, Toshio Miyata, Akiya Ogawa, Tadayuki Tsujita

**Affiliations:** 1 Center for Education and Research in Agricultural Innovation, Faculty of Agriculture, Saga University, 152-1 Shonan-cho, Karatsu, Saga 847-0021, Japan; skawa@cc.saga-u.ac.jp; 2 Department of Applied Chemistry, Graduate School of Engineering, Osaka Prefecture University, 1-1 Gakuen-cho, Naka-ku, Sakai, Osaka 599-8531, Japan; y.gonda.0402@gmail.com (Y.G.); 0tg38t18086577t@ezweb.ne.jp (T.Y.); su108027@edu.osakafu-u.ac.jp (Y.S.); h.shinohara@katayamakagaku.co.jp (H.S.); yohsuke.kobiki@gmail.com (Y.K.); sonoda@bioinfo.osakafu-u.ac.jp (M.S.); ogawa@chem.osakafu-u.ac.jp (A.O.); 3 Department of Molecular Medicine and Therapy, Tohoku University Graduate School of Medicine, 2-1 Seiryo-machi, Aoba-ku, Sendai, Miyagi 980-8575, Japan; ichimura.atsuhiko.2r@kyoto-u.ac.jp (A.I.); dantks@med.tohoku.ac.jp (T.D.); miyata@med.tohoku.ac.jp (T.M.); 4 Keihanshin Consortium for Fostering the Next Generation of Global Leaders in Research (K-CONNEX), Kyoto University, Kyoto 606-8501, Japan; 5 Department of Applied Biosciences, Graduate School of Life and Environmental Sciences, Osaka Prefecture University, 1-1 Gakuen-cho, Naka-ku, Sakai, Osaka 599-8531, Japan; 6 Department of Applied Biochemistry and Food Science, Faculty of Agriculture, Saga University, 1 Honjyo-machi, Saga, 840-8502, Japan

**Keywords:** hypoxia inducible factor, factor inhibiting hypoxia inducible factor, furan, thiophene

## Abstract

Induction of a series of anti-hypoxic proteins protects cells during exposure to hypoxic conditions. Hypoxia-inducible factor-α (HIF-α) is a major transcription factor that orchestrates this protective effect. To activate HIF exogenously, without exposing cells to hypoxic conditions, many small-molecule inhibitors targeting prolyl hydroxylase domain-containing protein have been developed. In addition, suppression of factor inhibiting HIF-1 (FIH-1) has also been shown to have the potential to activate HIF-α. However, few small-molecule inhibitors of FIH-1 have been developed. In this study, we synthesized a series of furan- and thiophene-2-carbonyl amino acid derivatives having the potential to inhibit FIH-1. The inhibitory activities of these compounds were evaluated in SK-N-BE(2)c cells by measuring HIF response element (HRE) promoter activity. Several furan- and thiophene-2-carbonyl amino acid derivatives inhibited FIH-1 based on correlations among the docking score of the FIH-1 active site, the chemical structure of the compounds, and biological HIF-α/HRE transcriptional activity.

## 1. Introduction

**Figure 1:**
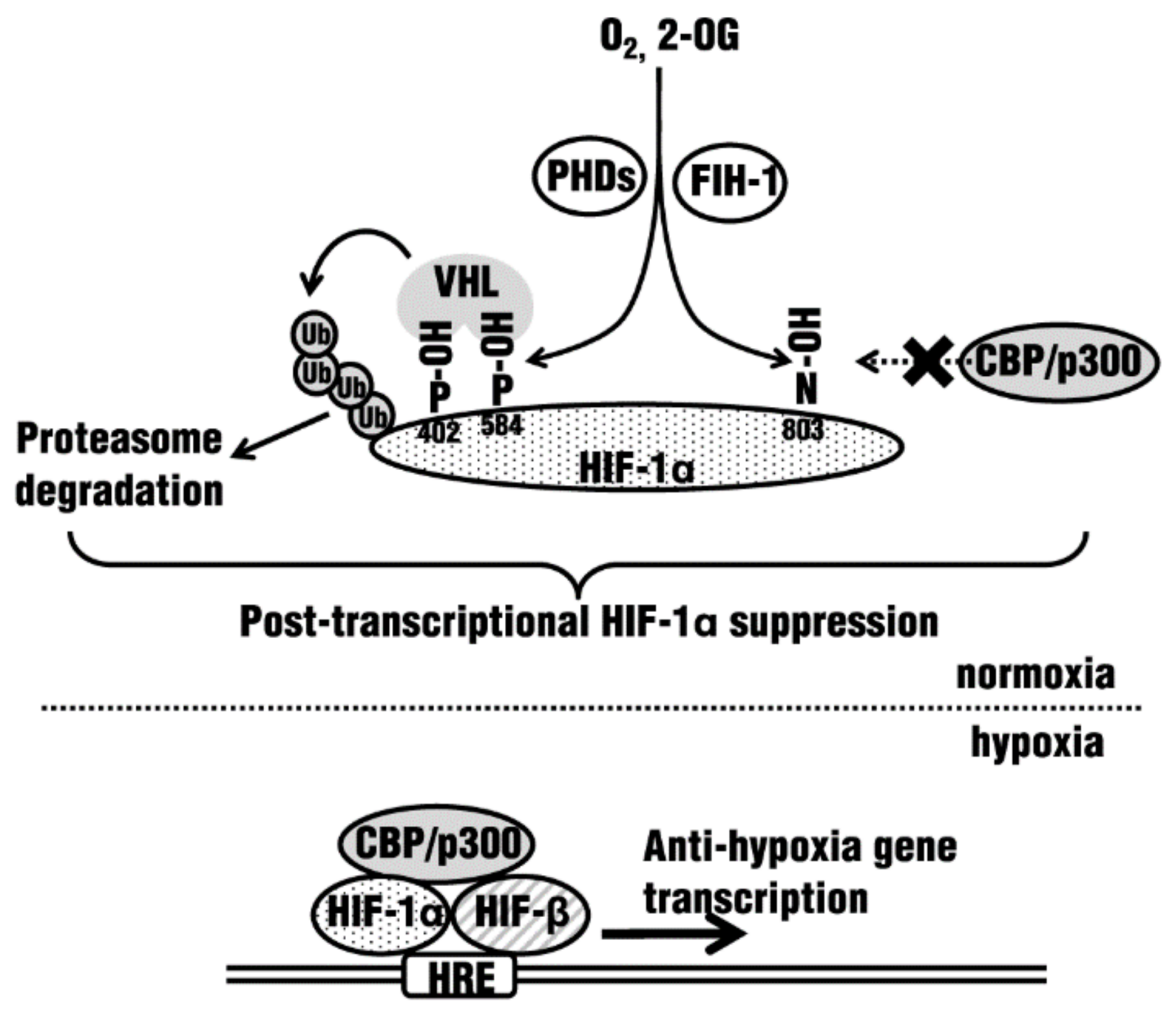
Regulatory mechanisms mediating the HIF-related pathway.

Hypoxia-inducible factors (HIFs) are pivotal transcription factors that contribute to the hypoxic stress response [1,2,3,4,5,6,7,8]. HIFs are classified into HIF-α and HIF-β subfamilies. HIF-α is hydroxylated by prolyl hydroxylase domain-containing proteins (PHD1, PHD2, and PHD3), and factor inhibiting HIF (FIH-1) under normoxic conditions (21% O_2_) [9,10,11,12] (Figure 1). Hydroxylated HIF-α is polyubiquitinated by von Hippel–Lindau E3 ubiquitin ligase (VHL) and rapidly degraded by the proteasome system. Enzymes that contribute to HIF-α quenching require molecular oxygen, 2-oxoglutarate (2OG), and ferric (Fe) ions. When cells encounter severe hypoxic conditions, HIF-α hydroxylation is inhibited due to low oxygen levels. Thus, the PHD/HIF-α molecular axis acts as an oxygen sensor system [1,2,3,4,5]. Under hypoxic conditions, HIF-α is stabilized and translocated to the nucleus, where it forms a heterodimeric complex with HIF-β. The HIF complex binds to HIF response element (HRE) ([A/G]CGTC) and transactivates genes that facilitate adaptation to hypoxic and/or ischemic stress, such as vascular endothelial growth factor (VEGF), erythropoietin (EPO), and glucose transporter-1 (GLUT-1).

To date, small molecules that can stabilize HIF-α via PHD inhibition have been widely investigated to treat ischemic heart failure, chronic kidney disease, and stroke [13,14,15,16,17,18,19,20,21,22,23,24,25,26,27,28]. On the other hand, under normoxic conditions, Asn803 on HIF-1α is hydroxylated and inhibited by FIH-1 [12]. This Asn803 hydroxylation blocks the interaction with the CREB-binding protein/p300 (CBP/p300) transcriptional coactivator. When cells are exposed to severe hypoxic conditions, FIH-1 fails to hydroxylate Asn803. Consequently, dehydroxylated HIF-1α can interact with CBP/p300 and contribute to the transactivation of anti-hypoxic proteins [9,10] (Figure 1). This mechanism has also been observed in FIH-1-knockout mice [29] and cells [30], however, selective small-molecule FIH-1 inhibitors [31,32,33,34] are still rare compared with PHD inhibitors [13,14,15,16,17,18,19,20,21,22,23,24,25,26,27,28]. Because HIF activation induced by PHD inhibition is different from that induced by FIH inhibition [35,36], the development of FIH-1 inhibitors is necessary.

Accordingly, in this study, we aimed to develop novel small-molecule inhibitors of FIH-1.

## 2. Results and Discussion

### 2.1. Design and Synthesis of New FIH-1 Inhibitors

**Figure 2:**
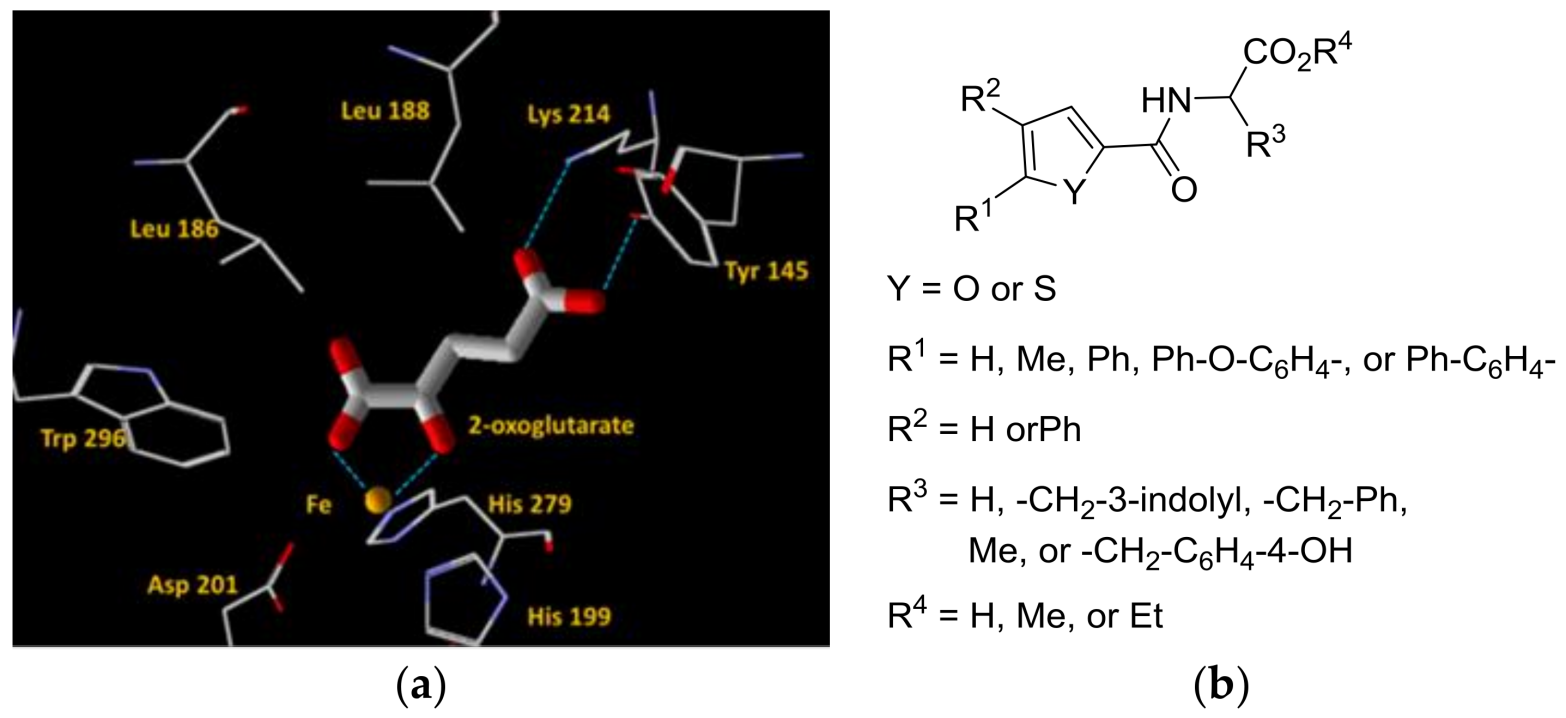
(**a**) The coordination of 2OG in the active center of FIH-1 (PBD ID: 1MZF); (**b**) The structure of the newly designed FIH-1 inhibitor structure based on the 2OG scaffold.

FIH-1 has an Fe atom in its active center, and this Fe is held in place by two histidine and aspartic acid residues. 2OG is a required cofactor that mediates FIH-1 activity. Based on X-ray crystallography data (PBD ID: 1MZF), 2OG binds the Fe atom bidentately, and forms a hydrogen bond with Lys214 and Tyr145 in human HIF-1 (Figure 2a) [37]. In this study, furan- and thiophene-2-carbonyl amino acid derivatives were designed to antagonize 2OG binding [38] based on the X-ray crystallography structure. The designed structure had a bidentate site for Fe atom binding and a carboxylic acid that could form a hydrogen bond with Lys214 and/or Tyr145 residues (Figure 2b). The R^1^ moiety contained an H, Me, phenyl, 4′-phenoxyphenyl, or 4′-biphenyl. The R^2^ moiety was prepared with either an H or phenyl group. The R^3^ moiety corresponded to an amino acid residue (glycine, alanine, phenylalanine, tryptophan, or tyrosine). A Me or Et group was introduced as the R^4^ moiety to increase the lipophilicity and cell membrane permeability. The R^4^ moiety was thought to be converted to H by hydrolysis in the cell. Many inhibitors have been reported to act as antagonists of 2OG [15,16,17,39]; however, the use of furan- and thiophene-2-carbonyl amino acid derivatives as antagonists of 2OG has not been evaluated.

**Scheme 1:**
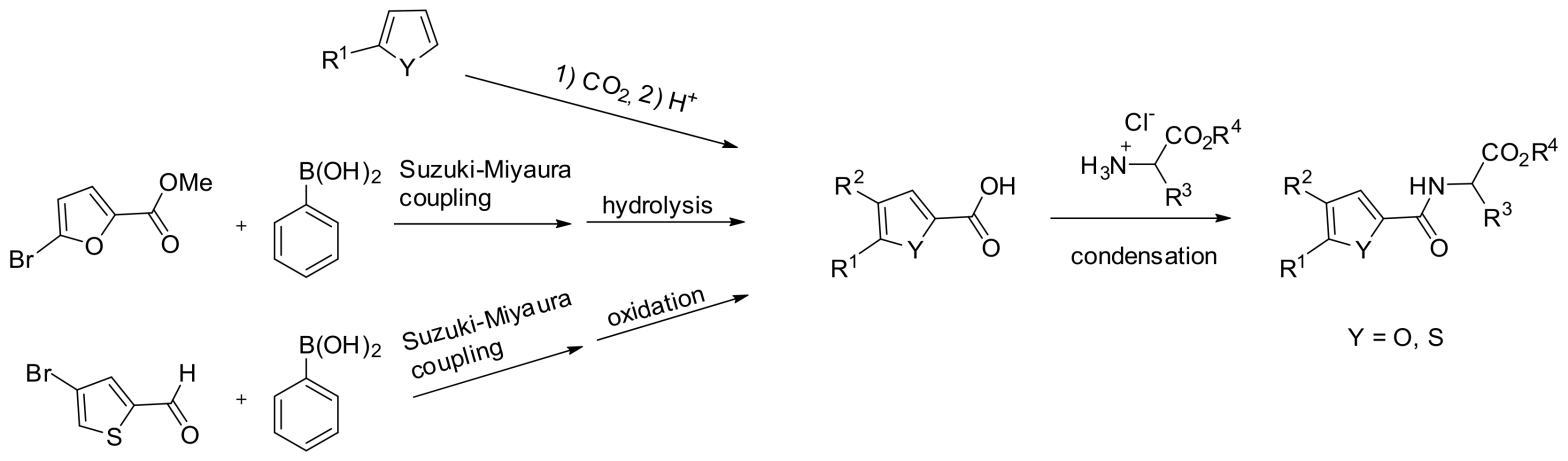
Synthetic routes of 2-carbonylfuran and 2-carbonylthiophene derivatives.

The synthetic strategy to design the compounds is summarized in Scheme 1. Furan- and thiophene-2-carbonyl amino acid derivatives were synthesized using condensation reactions of thiophene-2-carboxylic acids with amino acid salts. Nonsubstituted furan- or thiophene-2-carboxylic acids and benzofuran- or benzothiophene-2-carboxylic acids were obtained from commercial supplies. The other furan- or thiophene-2-carboxylic acids were synthesized as follows.

**Scheme 2:**
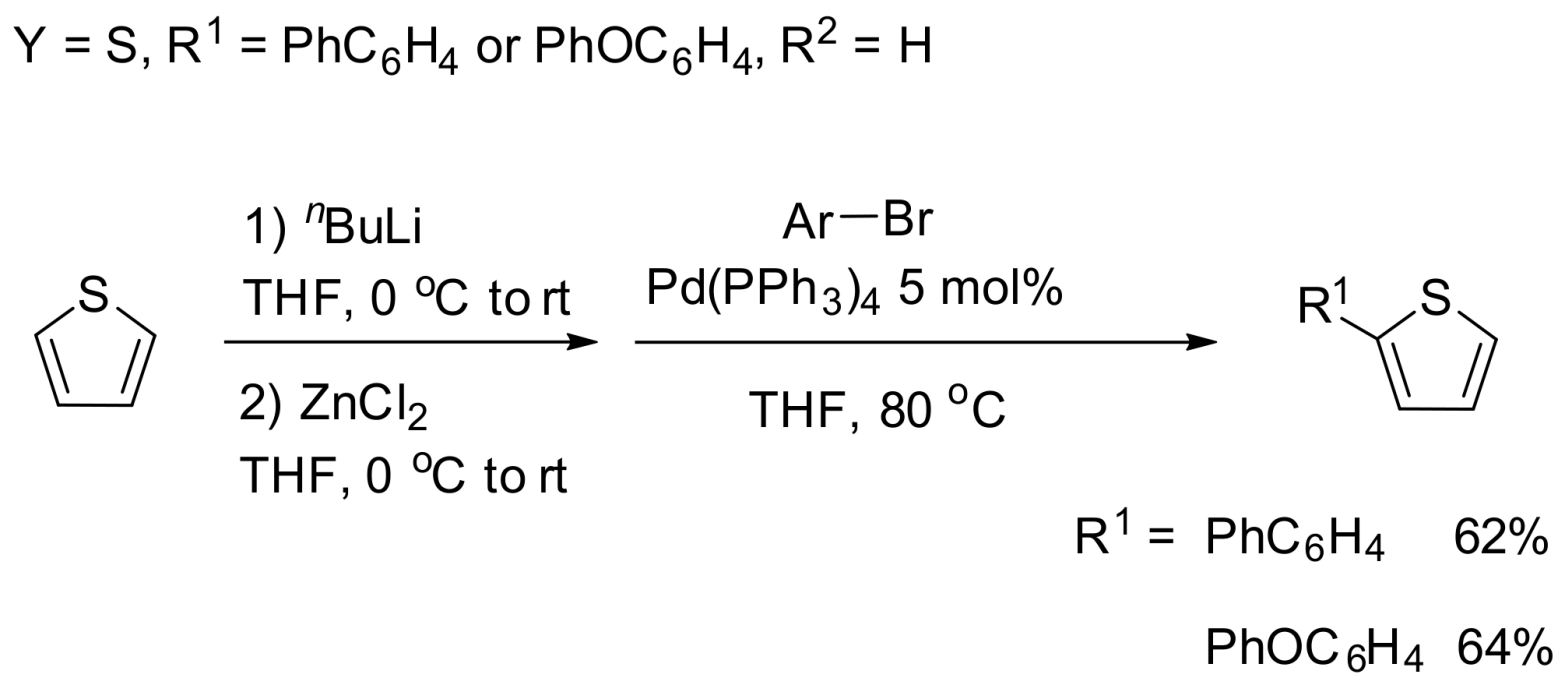
Preparation of 2-arylthiophene.

**Scheme 3:**
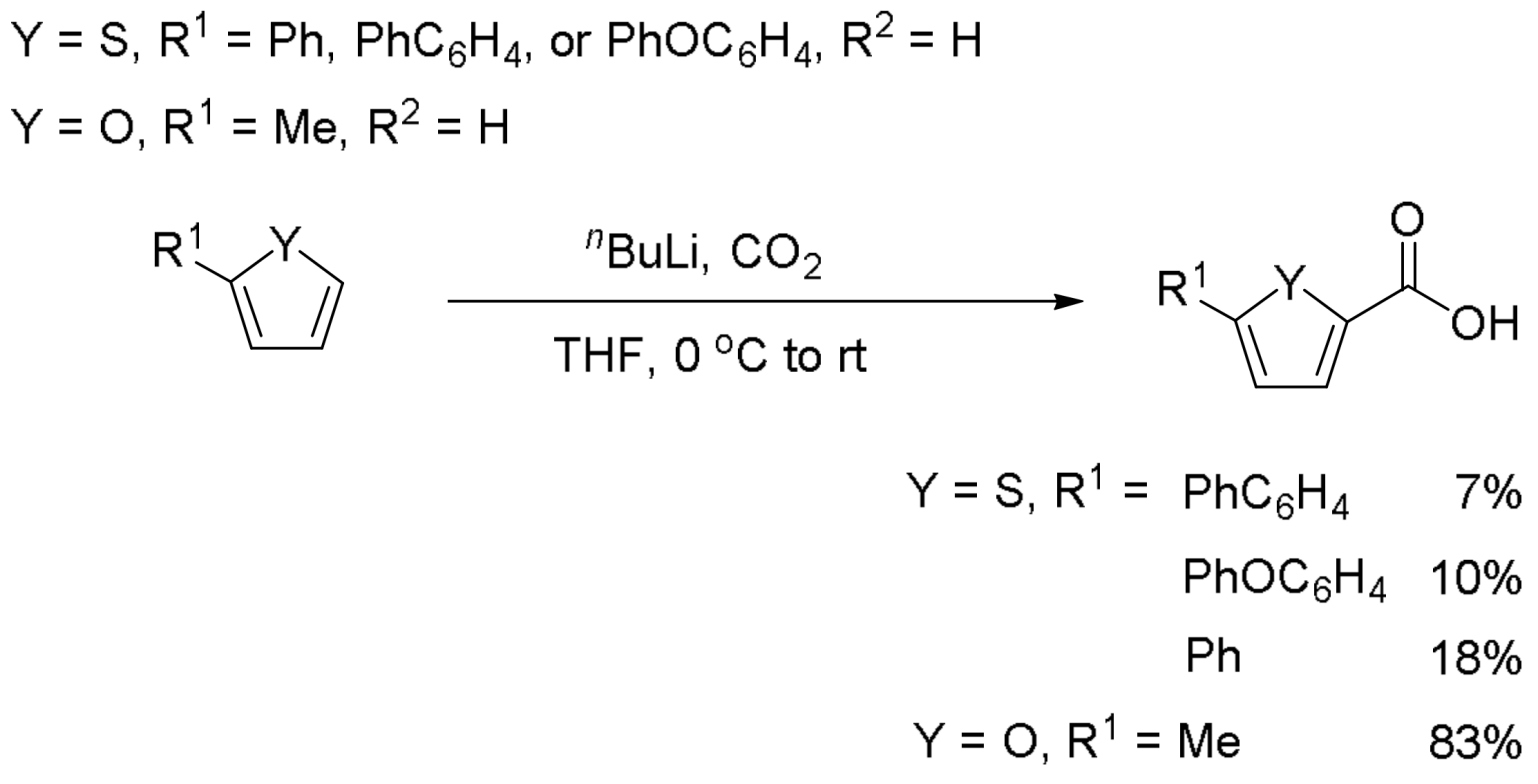
Preparation of 5-substituted thiophene-2-carboxylic acid and 5-methylfuran-2-carboxylic acid.

2-([1,1′-Biphenyl]-4-yl)thiophene and 2-(4-phenoxyphenyl)thiophene were prepared in moderate yields via a Negishi coupling reaction using the zinc salt of thiophene and aryl bromide (Scheme 2). Next, 5-substituted thiophene-2-carboxylic acids and 5-methylfuran-2-carboxylic acid were synthesized by carboxylating the corresponding furan or thiophene (Scheme 3).

**Scheme 4:**
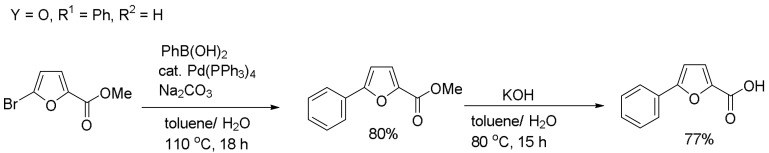
Preparation of 5-phenylfuran-2-carboxylic acid.

Preparation of 5-phenylfuran-2-carboxylic acid is shown in Scheme 4. Suzuki-Miyaura cross-coupling of phenyl boronic acid and methyl 5-bromofuran-2-carboxylate gave the coupling product, and subsequent hydrolysis produced the corresponding carboxylic acid in good yield.

**Scheme 5:**
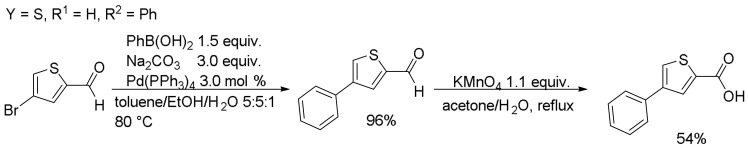
Preparation of 4-phenylthiophene-2-carboxylic acid.

Since the starting material was commercially available, the method for the synthesis of 4-phenylthiophene-2-carboxylic acid differed from that of 5-phenylfuran-2-carboxylic acid (Scheme 5). Suzuki-Miyaura cross-coupling of 4-bromo-2-thiophenecarbaldehyde and phenylboronic acid was used to obtain the corresponding coupling product, which yielded the corresponding carboxylic acid upon subsequent oxidation.

**Scheme 6:**
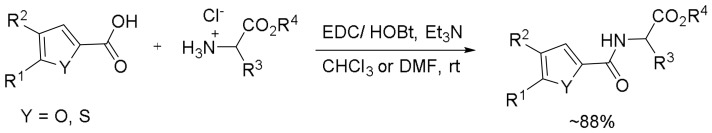
Condensation reactions with EDC.

For the condensation reaction, 1-ethyl-3-(3-dimethylaminopropyl)carbodiimide hydrochloride (EDC) was used as a condensation agent in the presence of 1-hydroxybenzotriazole (HOBt) (Scheme 6). The use of EDC/HOBt produced the condensation products in good yields and suppressed racemization of amide units.

### 2.2. Evaluation of HIF Activation by FIH-1 Inhibition

To evaluate HIF transactivation following FIH-1 inhibition in vivo, we employed a luciferase reporter assay system in the human neuroblastoma cell line SK-N-BE(2)c. The cells were stably transfected with a reporter vector possessing secretion-type luciferase (*Metridia longa* luciferase, MLuc) under control of the 7-time-repeat human *VEGFA* regulatory sequence (40 bp length containing the HRE) and mini TATA promoter. Hereafter these HRE reporter cells were designated SKN:HRE-MLuc cells [40]. To evaluate the inhibitory activity of FIH-1, SKN:HRE-MLuc cells were cultured under mild hypoxic conditions (3% O_2_) based on previous studies showing that under normoxic conditions (21% O_2_), FIH-1 inhibition does not significantly affect for HIF activation, whereas under 3% O_2_ conditions, FIH-1 inhibition elevates HIF transcriptional activity [1,30,41].

**Figure 3:**
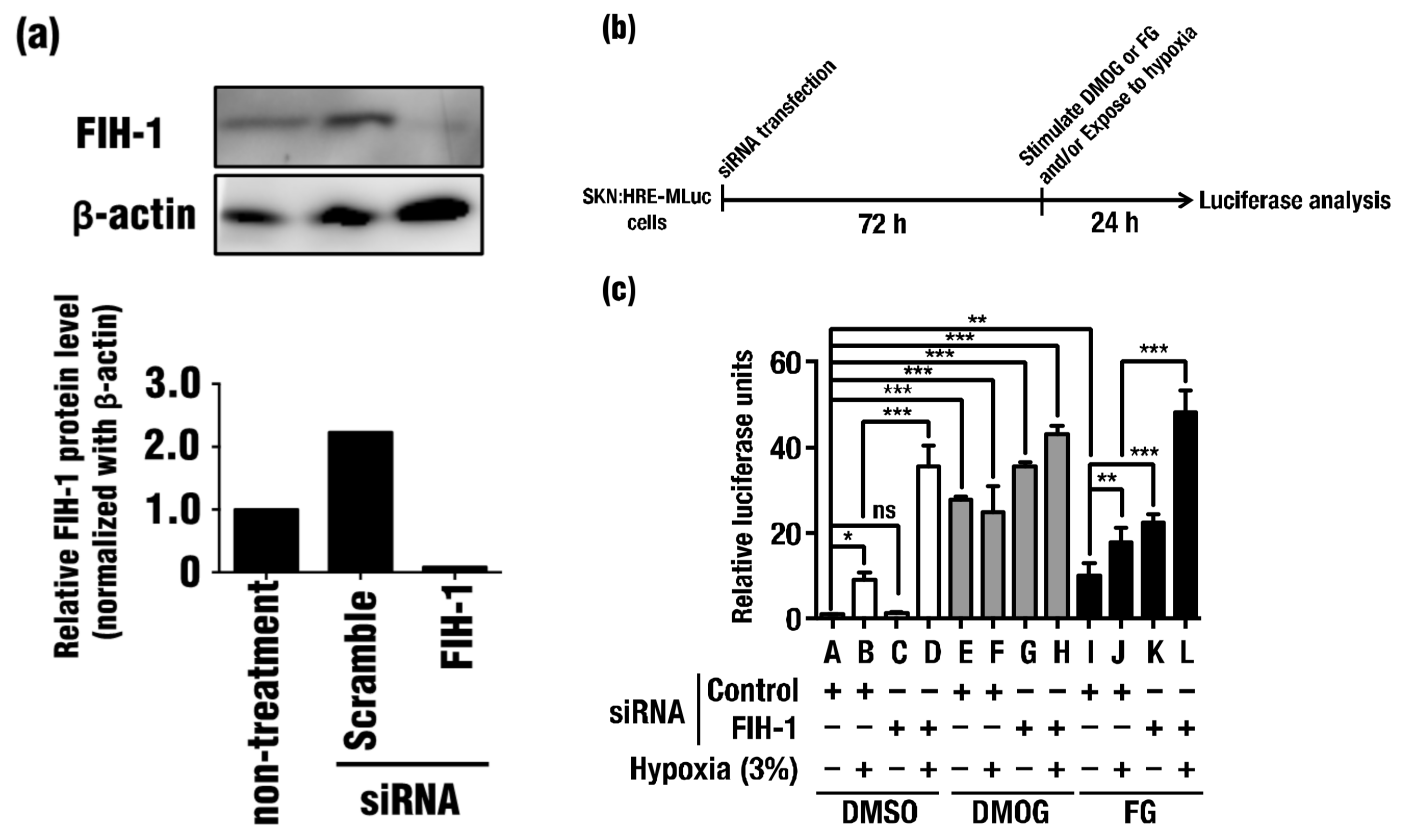
Establishment of the evaluation system for FIH-1 inhibitor activity. (**a**) Confirmation of siRNA knockdown efficiency for FIH-1. Scrambled siRNA and siRNA against FIH-1 were introduced into SK-N-BE(2)c cells. Transfected cells were cultured for 72 h with siRNA. After an additional 24 h, total cell lysates were analyzed by immunoblotting to detect FIH-1 or β-actin, which was used as an internal control; (**b**) Experimental design of the evaluation system for FIH-1 inhibitor activity; (**c**) HIF transcriptional activities were measured with secretion-type luciferase (*Metridia longa* luciferase, MLuc) based HRE transcriptional reporter analysis in SKN:HRE-MLuc reporter cells. To confirm the FIH-1 inhibitory activity, random target scrambled siRNA or siRNA against FIH-1 was transfected into SKN:HRE-MLuc cells, as indicated. The transfected cells were also treated with normoxic or mild hypoxic conditions (3% O_2_), DMOG (100 μM), or FG4592 (100 μM), as indicated. The degree of induction is presented as relative luciferase units, with the value from control siRNA, normoxia and DMSO treatment (column A) cells set as 1 for each treatment. All experiments were performed in triplicate. Data are means ± SEMs (*n* = 3). The statistical significance of results compared with data from the control group was calculated using one-way analysis of variance (ANOVA) with Newman-Keuls multiple-comparison test. ns, *p* > 0.05; * p = 0.05–0.01; ** p = 0.01–0.001; *** p < 0.001.

To confirm that our proposed system could be utilized to evaluate FIH-1 inhibitory activity, FIH-1 was transiently silenced by transfecting SKN:HRE-MLuc cells for 72 h with siRNA. After 24 h culture with fresh medium under normoxic conditions, FIH-1 protein was analyzed by immunoblotting. FIH-1 protein levels in FIH-1 siRNA transfectants were significantly reduced compared with that in untreated or scrambled siRNA-transfected control cells (Figure 3a). Using this FIH-1 siRNA system, we compared the efficacy of dimethyloxalyl glycine (DMOG) or FibroGen compound (FG4592) under normoxic or hypoxic conditions (3% O_2_) for 24 h (Figure 3b). Under hypoxic conditions, PHD proteins were mostly inactivated; therefore, the HIF-HRE top-up transcriptional activity under these conditions can be measured as FIH-1 inhibitory activity [30].

According to the experimental design indicated in Figure 3b, SKN:HRE-MLuc cells were transfected with the indicated siRNAs, after 72 h, the transfectants were treated with normoxic or hypoxic conditions (3% O_2_) for 24 h, and the luciferase activities were determined (Figure 3c). HIF transcriptional activity on SKN:HRE-MLuc cells was significantly elevated during hypoxia treatment (compare A with B). Moreover, HIF was not stabilized in FIH-1 knockdown cells under normoxic conditions (compare A with C). In contrast, under hypoxic conditions, HIF transcriptional activity was enhanced in FIH-1 knockdown cells, supporting the inhibitory activity of FIH-1 (compare B with D). Treatment with DMOG, which inhibits both PHDs and FIH-1, resulted in higher HIF stabilization activity (compare A with E, F, G, or H). On the other hand, treatment with FG4592, which is a selective inhibitor of PHD [36,42], stabilized HIF compared with vehicle-treated cells (compare A with I). The difference between E and I was supported by changes in FIH-1 activity. Therefore, FG4592 treatment under hypoxic conditions only slightly stabilized HIF (compare I with J). Importantly, FG4592 treatment did not affect FIH-1 inhibitory activity. Additionally, SKN:HRE-MLuc cells were significantly activated following FIH-1 knockdown treatment under mild hypoxic conditions, even in the presence of FG4592 (compare J with L). Taken together, these results suggested that measuring HIF-HRE transcriptional activity with continuous mild hypoxia may reflect FIH-1 activity.

**Figure 4:**
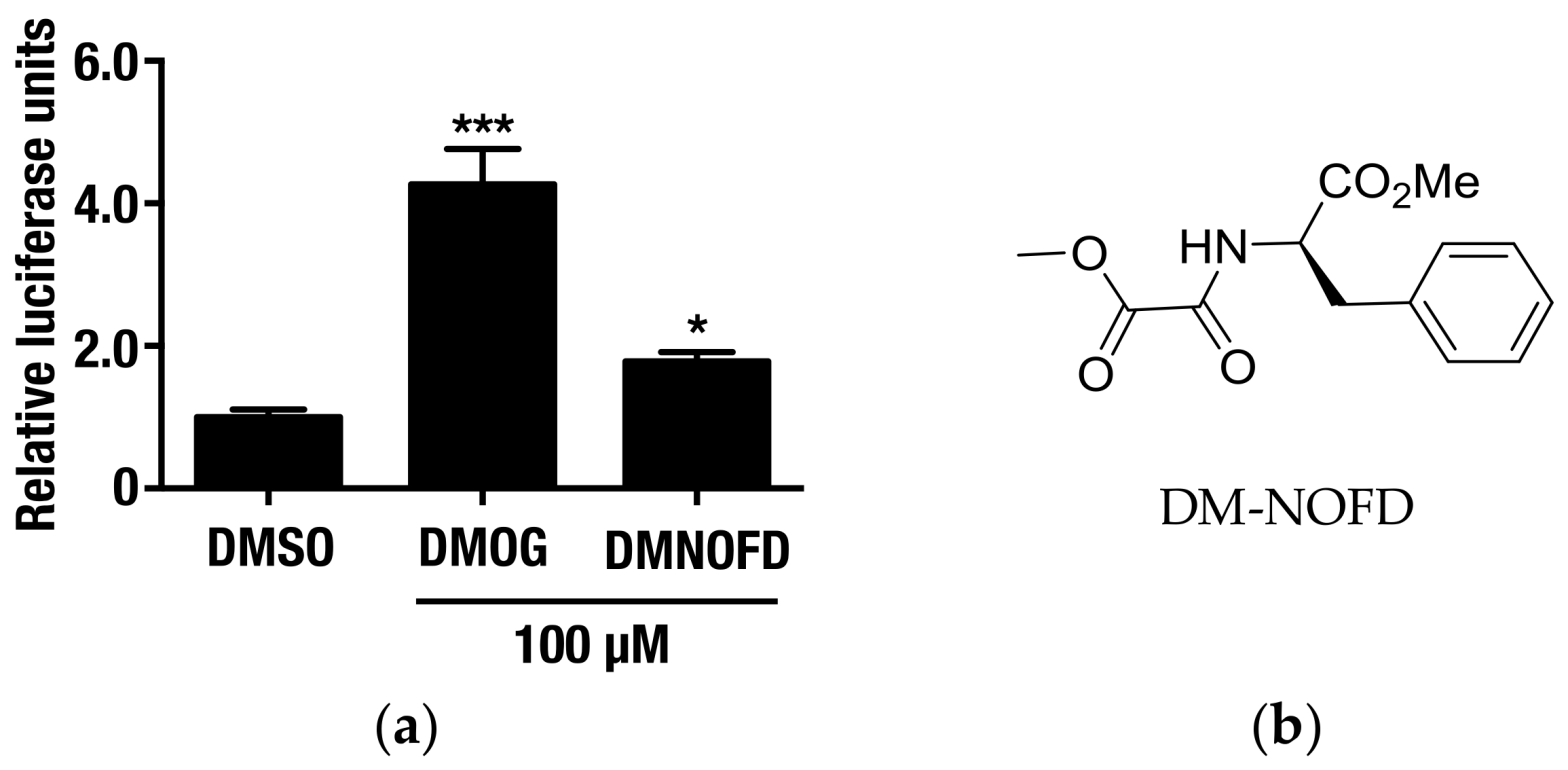
(**a**) Evaluation of HIF activation using an FIH-1 inhibitor (DM-NOFD). SKN:HRE-MLuc cells were treated with DMSO (1%), DMOG (100 μM) or DM-NOFD (100 μM) for 24 h under hypoxic conditions (3% O_2_). The degree of induction is presented as relative luciferase units, with the value from DMSO-treated cells set as 1 for each treatment. Experiments were performed in triplicate. Data are means ± SEMs (*n* = 3). The statistical significance of results compared with data from the control group was calculated using one-way ANOVA with Newman-Keuls multiple-comparison tests. * p = 0.05 to 0.01; *** p < 0.001; (**b**) Chemical structure of DM-NOFD.

Next, to confirm that the proposed system could be used to evaluate FIH-1 inhibition, we treated the cells with DMOG, which can inhibit both PHD and FIH as a positive control, or dimethyl *N*-oxalyl-d-phenylalanine (DM-NOFD), which is a prodrug of *N*-oxalyl-d-phenylalanine, a reported FIH-1-selective inhibitor (Figure 4b) [31,34]. Treatment with DM-NOFD (100 μM) significantly enhanced HIF activity (Figure 4a). This result indicated that our FIH-1 evaluation system could be utilized for quantification of further optimized small compounds derived from DM-NOFD. 

### 2.3. Evaluation of HIF Activation by Furan- and Thiophene-2-Carbonyl Amino Acid Derivatives under Hypoxic Conditions

Next, we evaluated the activation of HIF by furan- and thiophene-2-carbonyl amino acid derivatives using the evaluation methods described above. Simultaneously, we investigated cellular toxicity for those derivatives with a 3-(4,5-dimethylthiazol-2-yl)-5-(3-carboxymethoxyphenyl)-2-(4-sulfophenyl)-2*H*-tetrazolium (MTS) assay. To indicate cellular membrane permeability, the partition coefficients, which are frequently represented by calculated logP (clogP) are listed in Table 1 and Table 2.

Compounds **1**, **7**, **9**–**11**, **15**–**18**, **28**, **30**, and **38** stabilized HIF and transactivated the HRE-driven MLuc reporter in SKN:HRE-MLuc cells. Some trends were observed among the active compounds. For example, compounds with bulky R^1^ groups, such as Ph, 4-Ph-C_6_H_4_-, and 4-PhO-C_6_H_4_-, did not enhance HIF activity (Entry 19–25, 31–36). Some active compounds were obtained when the R^3^ group was an aromatic group, such as Ph-CH_2_- or 3-indolyl-CH_2_ (Compounds **7**, **9**–**11**, **15**–**18**, **28**, **30**, **38**). In some cases, both the *S* and *R* isomer had activity (**10**, **11** and **17**, **18,** respectively). Both furan and thiophene derivatives stabilized HIF activity. Only compound **30** showed cellular toxicity in the MTS assays.

### 2.4. Docking Simulations Using Furan- and Thiophene-2-Carbonyl Amino Acid Derivatives with FIH-1 

**Figure 5:**
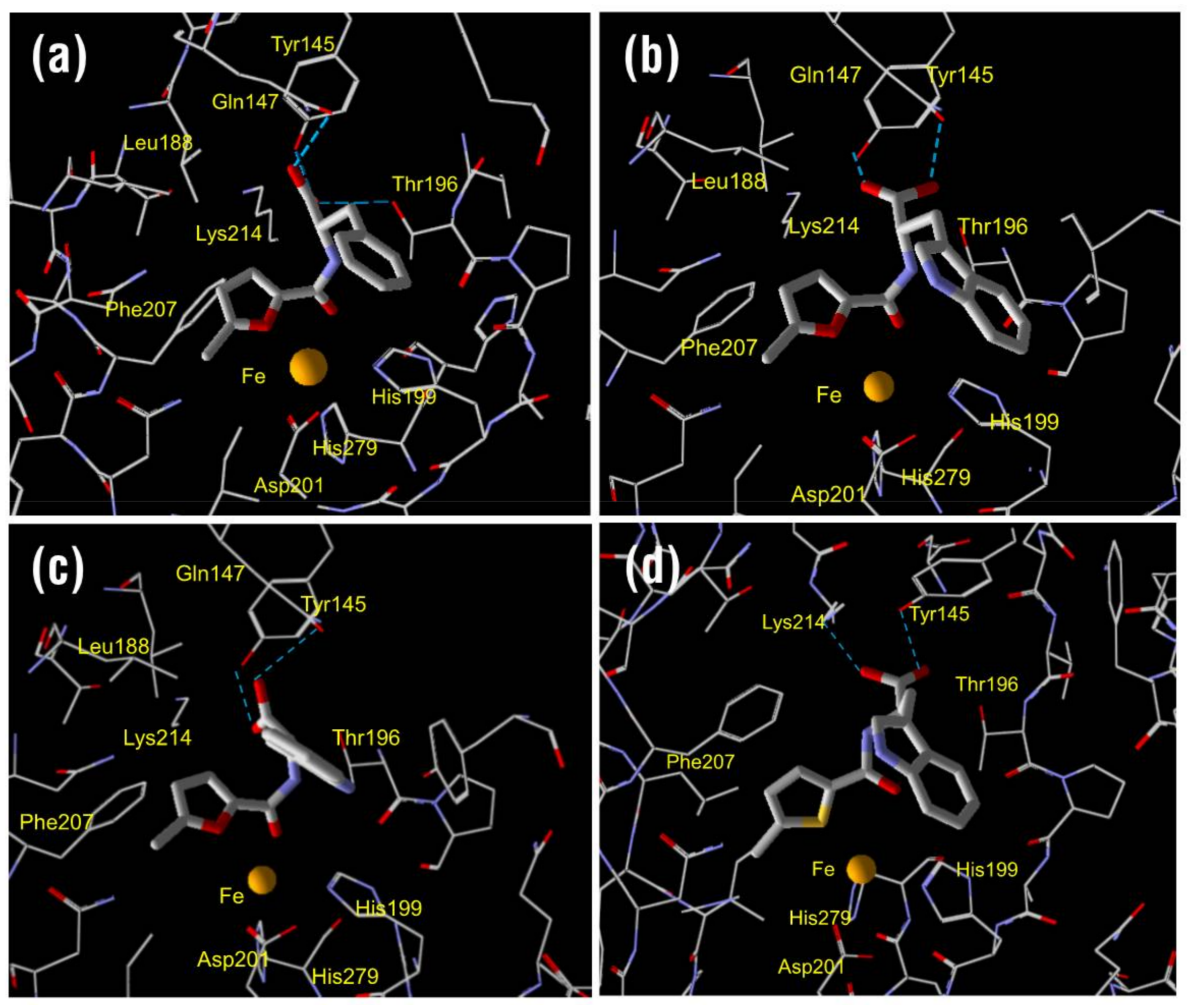
The predicted binding model of compound **7**, **9**, **10** (hydrolysate)**,** and **16** within the active center of FIH-1. (**a**) Compound **7** (hydrolysate); (**b**) Compound **9** (hydrolysate); (**c**) Compound **10** (hydrolysate); and (**d**) Compound **16**.

To understand the inhibitory tendencies observed in HRE-MLuc reporter assays on SKN:HRE-MLuc cells, docking simulations with FIH-1 were performed with four compounds (**7, 9**, **10**, and **16**); among the compounds that stabilized HIF activity (i.e., compounds **7**, **9**–**11**, **15**–**18**, **28**, **30,** and **38**), these four compounds had characteristic scaffolds. Docking simulations were run on Molegro Virtual Docker 6.0.0 [43]. The corresponding hydrolyzed compounds were used in the docking simulations (compounds **7**, **9**, and **10**). The results of the FIH-1 docking studies are shown in Table 3. For compound **7**, a furan derivative with a bulky group (such as Ph-CH_2_-) in the R^3^ position, and compound **9**, a furan derivative with a 3-indolyl-CH_2_ group in the R^3^ position, both dockings had good scores (entries 1 and 2). The predicted binding models of compounds **7** and **9** within the active center of FIH-1 are shown in Figure 5a,b. When compound **9**, which had an l-tryptophan scaffold, was compared with compound **10**, which had a d-tryptophan scaffold, good docking scores were obtained (Table 3, entries 2 and 3). The predicted binding models of compounds **9** and **10** showed that both 3-indolyl-CH_2_ groups in the R^3^ position fit within the space, although compounds **9** and **10** were enantiomers to each other (Figure 5b,c).

The thiophene compound **16**, which was related to compound **9**, also docked within FIH-1; however, it had a lower score (Table 3, entry 4). The predicted binding model of compound **16** showed a similar conformation (Figure 5d). These results supported the notion that compounds with bulky groups in the R^1^ position did not show the activity because there was not sufficient space ahead of the R^1^ position. In contrast, compound **1** did not selectively inhibit FIH-1 and could inhibit other enzymes that used 2OG as a cofactor because of its scaffold.

### 2.5. Inactibation of HIF by FIH-1 Inhibitors under Normoxic Conditions

**Figure 6:**
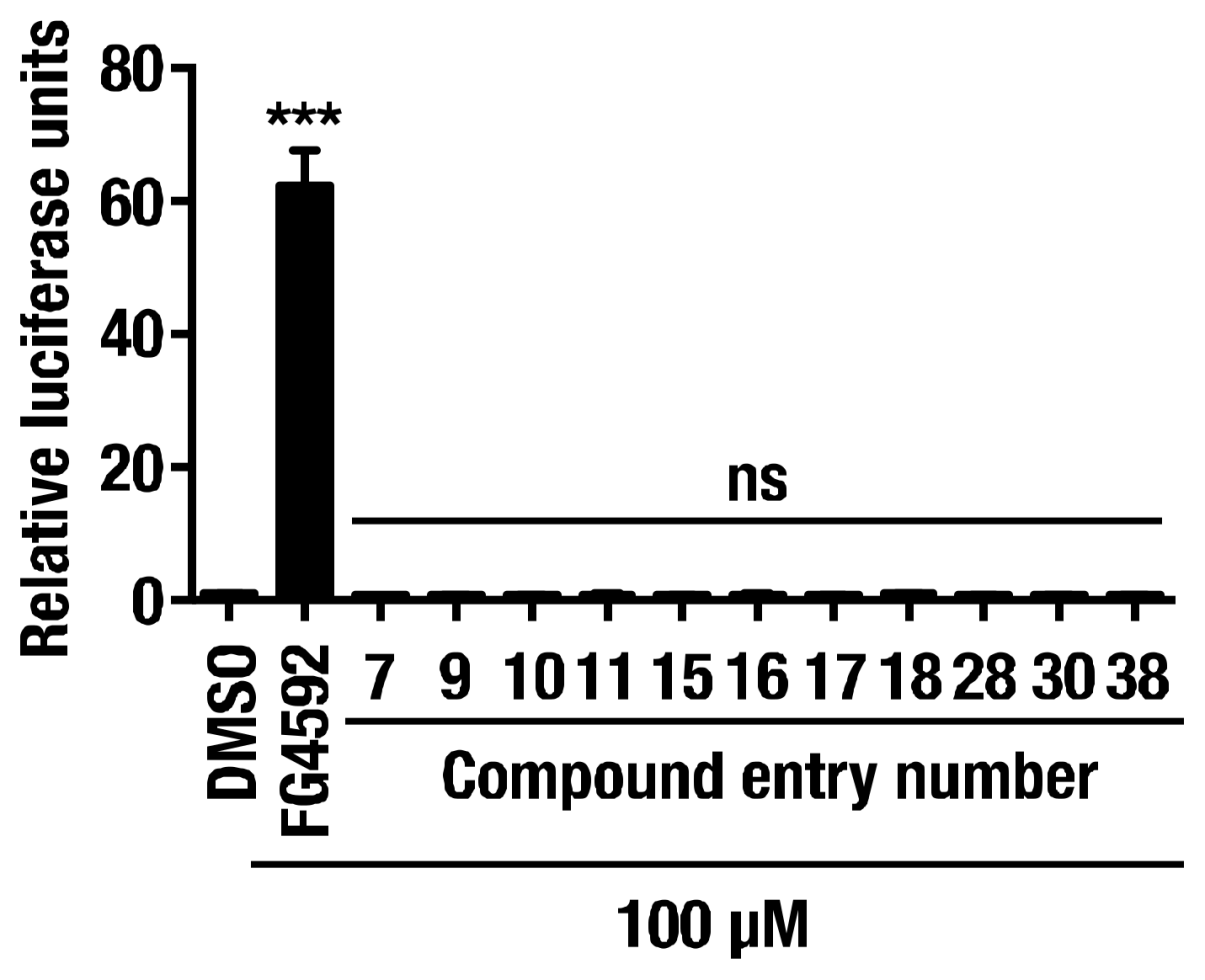
HIF activation using the identified compounds (**7**, **9**–**11**, **15**–**18**, **28**, **30**, and **38**) under normoxic conditions. The degree of induction is presented as relative luciferase units, with the value from DMSO treatment of cells set as 1 for each treatment. Experiments were performed in triplicate. Data are means ± SEMs (*n* = 3). The statistical significance of the results compared with data from the control group was calculated using one-way ANOVA with Newman-Keuls multiple-comparison tests. ns, *p* > 0.05; *** p < 0.001.

We next measured HIF transactivity of FIH-1 inhibitors under normoxic conditions to confirm whether these inhibitors stabilized HIF via inhibiting PHD. Under normoxic conditions, inhibition of PHD dramatically activated HRE reporter. In contrast, when PHD was inhibited under hypoxic conditions, the rate of activation of the HRE reporter was low because hypoxia already partially activated the HRE reporter. The compounds showing HIF activation under hypoxic conditions (compounds **7**, **9**–**11**, **15**–**18**, **28**, **30**, and **38**) were evaluated by the luciferase assays in SKN:HRE-MLuc cells under normoxic conditions (Figure 6). Treatment with 100 μM FG4592, a commonly used selective inhibitor of PHD, was used as a positive control and showed significant HRE reporter activity. In contrast, compounds **7**, **9**–**11**, **15**–**18**, **28**, **30**, and **38** did not elicit any HIF transactivation. This result indicated that these compounds selectively inhibited FIH-1. At the same time, these compounds (**7**, **9**–**11**, **15**–**18**, **28**, **30**, and **38**) did not fit into the catalytic domain of the PHD2 (data not shown).

### 2.6. mRNA Expression in SK-N-BE(2) Cells

**Figure 7:**
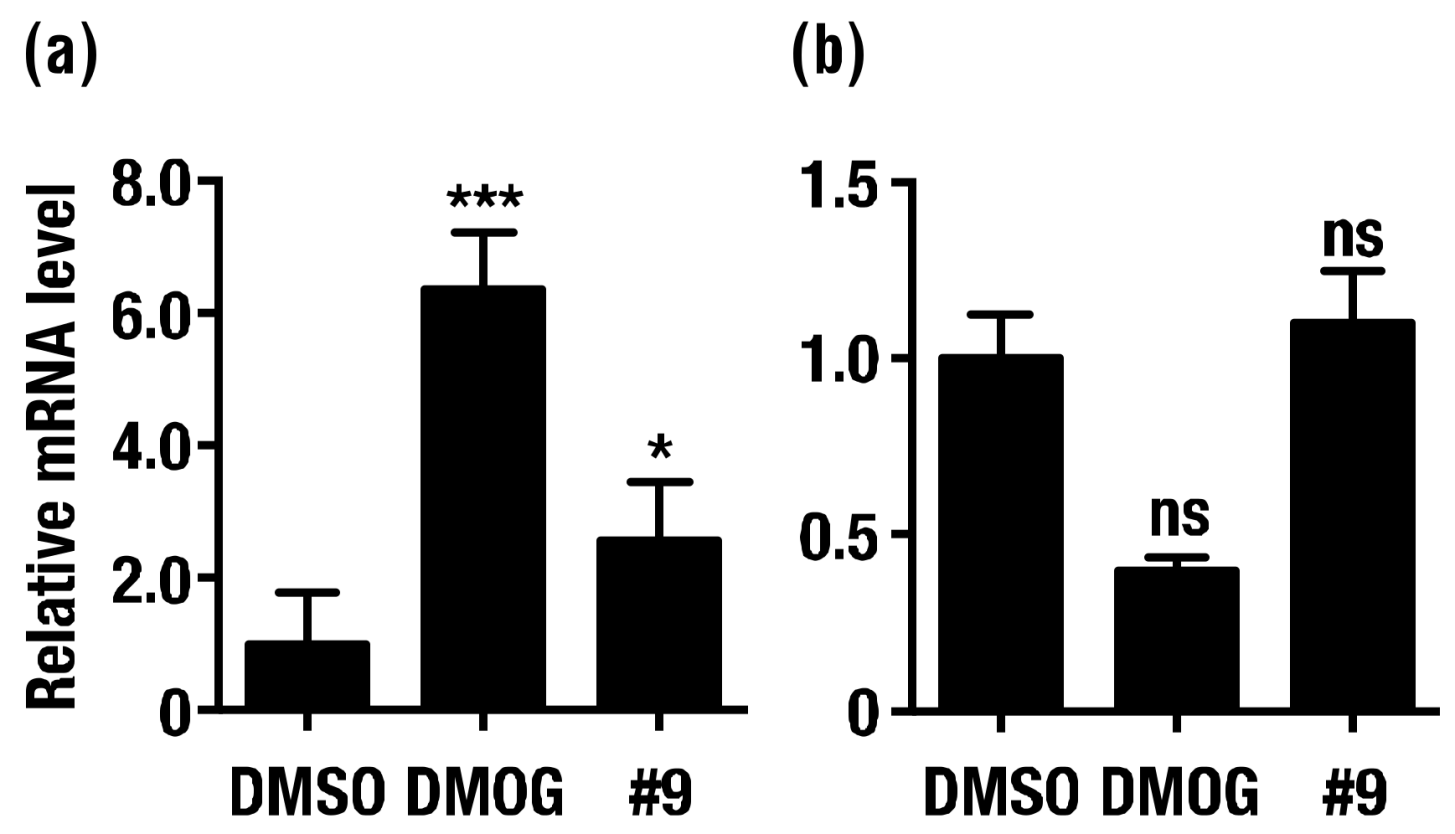
Expression levels of *CA9* (**a**) and *HIF-1α* mRNA. (**b**) SK-N-BE(2)c cells were stimulated with DMOG (100 μM) and compound **9** (100 μM) under hypoxic conditions (3% O_2_). Gene induction is presented as relative mRNA levels, with the qRT-PCR values from DMSO-treated cells set as 1 for each treatment. Experiments were performed in triplicate. Data are means ± SEMs (*n* = 3). The statistical significance of the results compared with data from the control group was calculated using one-way ANOVA with Newman-Keuls multiple-comparison tests. ns, *p* > 0.05; * p < 0.05–0.01; *** p < 0.001.

To confirm transactivation of HIF by our optimized compounds, the expression of HIF target genes was analyzed with quantitative polymerase chain reaction (PCR) in SK-N-BE(2)c cells. We attempted to analyze several HIF target genes including *CA9*, *VEGF-α*, *EPO*, and *PHD3*, under 3% O_2_ conditions. Among these genes, as described previously [30], *CA9* was significantly elevated when treated with compound **9** (Figure 7a). Notably, this induction of *CA9* did not occur via *HIF-1α* mRNA transactivation (Figure 7b). In addition, de novo translation of *HIF-1α* mRNA was slightly suppressed by DMOG treatment but not by treatment with compound **9**.

## 3. Conclusions

In this study, we used SKN:HRE-MLuc cells cultured under mild hypoxic conditions to reflect FIH-1 activity even in the presence of PHD proteins. To evaluate FIH-1 function more specifically, N to C transactivation domain of the HIF-1α (amino acids 531–826) and GAL4 fusion protein with a GAL4-DNA binding domain-driven reporter system would be a powerful alternative evaluation method and high-throughput screening system [44]. Additionally, we designed and synthesized novel FIH-1 inhibitors having furan- or thiophene-2-carbonyl amino acids as the main scaffold. Several compounds showed HIF activation potential using SKN:HRE-MLuc cells under mild hypoxic conditions. These compounds tended to have bulky lipophilic moieties in the R^3^ position. The results of docking simulations and luciferase assays under normoxic conditions supported the idea that the identified compounds activated HIF transcription by FIH-1 inhibition. Selective FIH-1 inhibitors are still rare; therefore, the identified compounds may provide alternative HIF activation tools.

## 4. Materials and Methods 

### 4.1. General Information

DMSO, DMOG, and FG4592 were obtained from Merck (Darmstadt, Germany), and other general reagents were purchased from Nacalai Tesque (Kyoto, Japan). DM-NOFD was synthesized according to a literature method [45].

### 4.2. Preparation of 2-([1,1′-biphenyl]-4-yl)thiophene and 2-(4-phenoxyphenyl)thiophene [46]

Under an N_2_ atmosphere, thiophene (1.5 g, 18 mmol) was placed in a three-necked flask with distilled tetrahydrofuran (THF, 30 mL). The flask was cooled at 0 °C, and *n*-butyllithium (*ca*. 1.6 M in hexane, 10 mL, 16 mmol) was added dropwise. After addition, the mixture was stirred at room temperature for 30 min. The flask was cooled at 0 °C again, and dried zinc chloride (2.0 g, 15 mmol) suspended in THF (10 mL) was added dropwise. After addition, the mixture was stirred at room temperature for 30 min., 4-bromobiphenyl or 4-bromodiphenyl ether (15 mmol) and Pd(PPh_3_)_4_ (0.69 g, 0.6 mmol) in THF (10 mL) were added, and the mixture was stirred at 80 °C. After stirring for 16 h, the mixture was cooled to room temperature and quenched with water. The resulting mixture was extracted with diethyl ether and dried with MgSO_4_. After filtration, the solvent was removed under reduced pressure. The desired pure thiophene was obtained after purification by column chromatography on silica gel.

### 4.3. Preparation of 2-((1,1′-biphenyl)-4-yl)thiophene-2-carboxylic acid, 2-(4-phenoxyphenyl)thiophene-2-carboxylic acid, 5-phenylthiophene-2-carboxylic acid, and 5-methylfuran-2-carboxylic acid [47]

2-Methylfuran was obtained from commercial supplies. 2-Phenylthiophene was prepared according to a previously described method [48]. Under an N_2_ atmosphere, 2-([1,1′-biphenyl]-4-yl)thiophene, 2-(4-phenoxyphenyl)thiophene, or 2-methylfuran (1.0 equiv.) was placed in a three-necked flask with distilled THF or Et_2_O. The flask was cooled at 0 °C, and *n*-butyllithium (1.1 equiv.) was added dropwise over a period of 15 min. The mixture was then stirred for 30 min at room temperature. After the mixture was cooled at −78 °C with a dry ice/acetone bath, CO_2_ gas was introduced under vigorous stirring and cooling. After stirring for 2 h, the mixture was warmed to room temperature and quenched with water. The ether layer was separated and washed with water. The combined solution was acidified with a 3 N HCl solution at 0 °C, and then extracted with EtOAc. The organic layer was washed with brine, dried with MgSO_4_, and concentrated under reduced pressure. The desired products were obtained without further purification.

### 4.4. Preparation of 5-phenylfuran-2-carboxylic acid

Methyl 5-bromofuran-2-carboxylate and phenylboronic acid were obtained from commercial supplies. Under an N_2_ atmosphere, methyl 5-bromofuran-2-carboxylate (26.8 g, 131 mmol), phenylboronic acid (20.7 g, 133 mmol), toluene (30 mL), Na_2_CO_3_ (2 M, 70 mL), and Pd(PPh_3_)_4_ (7.7 g, 6.5 mmol) were added to a three-necked flask. The mixture was refluxed for 18 h. The reaction mixture was extracted with EtOAc, dried over MgSO_4_, filtered, and concentrated under reduced pressure. The crude product was purified by column chromatography on silica gel (eluent: hexane/Et_2_O = 9/1) to obtain the product. In a three-necked flask, methyl 5-phenylfuran-2-carboxylate (14.9 g, 63 mmol), toluene (40 mL), and KOH (2 M, 400 mL) were added. The mixture was heated at 70 °C for 15 h. EtOAcwas added to the reaction solution, and the water layer was extracted. HCl aq. (3 N) was added to the water layer at 0 °C, and the solution was extracted with EtOAcand washed with brine. The combined organic phases were dried over Na_2_SO_4_, filtered, and concentrated under reduced pressure. 5-Phenylfuran-2-carboxylic acid was obtained as a white solid and its structure confirmed by ^1^H nuclear magnetic resonance (NMR) [49].

### 4.5. Preparation of 4-phenylthiophene-2-carboxylic acid

4-Bromo-2-thiophenecarbaldehyde and phenylboronic acid were obtained from commercial suppliers. 4-Bromo-2-thiophenecarbaldehyde (1.9 g, 10 mmol) was added to a solution of phenylboronic acid (1.8 g, 15 mmol), Na_2_CO_3_ (3.2 g, 30 mmol), Pd(PPh_3_)_4_ (0.35 g, 0.4 mmol) in toluene (15 mL)/EtOH (15 mL), and the mixture was refluxed for 12 h. The reaction was filtered with silica gel and concentrated. The resulting residue was purified by column chromatography on silica gel to give 4-phenyl-2-thiophenecarbaldehyde (96%). The product was confirmed by ^1^H-NMR.[50] Then KMnO_4_ (1.7 g, 11 mmol) in water (15 mL) was added dropwise to a solution of 4-phenyl-2-thiophenecarbaldehyde (1.8 g, 9.5 mmol) in acetone (10 mL), and the mixture was refluxed for 12 h. After cooling, the reaction mixture was filtered with silica gel. The mixture was extracted with Et_2_O and saturated NaHCO_3_ aq. (3 × 20 mL). The aqueous layer was neutralized with 3 N HCl aq. and extracted with Et_2_O (3 × 20 mL). The resulting organic layer was washed with brine, dried over Na_2_SO_4_, and concentrated. The crude product was purified by column chromatography on silica gel to obtain 4-phenylthiophene-2-carboxylic acid (53%). The product was confirmed by ^1^H-NMR [51]. 

### 4.6. General Procedure for Furan- and Thiophene-2-Carbonyl Amino Acid Derivatives: Condensation Reaction of Furan- and Thiophene-2-Carboxylic Acids with Amino Acid Ester Hydrochlorides

Furan-2-carboxylic acid, thiophene-2-carboxylic acid, 5-methylthiophene-2-carboxylic acid, benzofuran-2-carboxylic acid, benzothiophene-2-carboxylic acid, and amino acid ester hydrochlorides were obtained from commercial supplies and were used without further purification. To a two–necked flask, an amino acid ester hydrochloride (1.1 mmol), 1–ethyl–3–dimethylaminopropylcarbodiimide hydrochloride (EDC, 0.29 g, 1.5 mmol), HOBt (0.20 g, 1.5 mmol), Et_3_N (0.42 mL, 3.0 mmol), and CHCl_3_ or DMF (2.2 mL) were added, and the mixture was stirred at 0 °C for 10 min. Furan-2-carboxylic acid or thiophene-2-carboxylic acid dissolved in DMF (2.2 mL) was added, and then stirred for 17 h. The reaction mixture was diluted with water and extracted with EtOAc (3 × 20 mL). The organic phase was then washed with 3 N HCl aq. (3 × 20 mL), saturated NaHCO_3_ aq. (3 × 20 mL), and brine (20 mL). The combined organic phases were dried over Na_2_SO_4_, filtered, and concentrated under reduced pressure. The residue was purified by column chromatography on silica gel (eluent: hexane/EtOAc = 1/1) to obtain the product. The purity of the product was confirmed by ^1^H-NMR. Stereochemistry of the final product was measured by high-performance liquid chromatography (HPLC) with a chiral column (Compound **16**). The chart is shown in the supporting information.

Compounds **39** [52], **41** [53] were previously characterized. The analytical data of the final products are described below.

*Methyl (furan-2-carbonyl)glycinate* (**1**) was synthesized from furan-2-carboxylic acid and glycine methyl ester hydrochloride; yield 76%; colorless oil; ^1^H-NMR (400 MHz, CDCl_3_) δ 3.80 (s, 3H), 4.23 (d, *J* = 5.2 Hz, 2H), 6.51 (dd, *J* = 3.2 Hz, 1.6 Hz, 1H), 7.15 (d, *J* = 3.2 Hz, 1H), 7.47 (d, *J* = 1.6 Hz, 1H); ^13^C-NMR (100 MHz, CDCl_3_) δ 40.9, 52.6, 112.3, 114.9, 144.3, 147.4, 158.4, 170.3; HRMS (FAB) Calcd for C_8_H_10_NO_4_ [M + H]^+^: 184.0610, Found: 184.0639.

*Methyl (thiophene-2-carbonyl)glycinate (***2**) was synthesized from thiophen-2-carboxylic acid and glycine methyl ester hydrochloride; yield 81%; white solid; ^1^H-NMR (400 MHz, CDCl_3_) δ 3.79 (s, 3H), 4.22 (d, *J* = 5.2 Hz, 2H), 6.72 (br, 1H), 7.07 (dd, *J* = 4.0 Hz, 4.8 Hz, 1H), 7.49 (d, *J* = 5.2 Hz, 1H), 7.57 (d, *J* = 4.4 Hz, 1H); ^13^C-NMR (100 MHz, CDCl_3_) δ 41.6, 52.6, 127.8, 128.7, 130.6, 138.1, 162.1, 170.5; HRMS (FAB) Calcd for C_8_H_10_NO_3_S [M + H]^+^: 200.0381, Found: 200.0406.

*(S)-Methyl (furan-2-carbonyl)tryptophanate* (**3**) was synthesized from furan-2-carboxylic acid and l-tryptophan methyl ester hydrochloride; yield 88%; white solid; ^1^H-NMR (400 MHz, CDCl_3_) δ 3.41 (dd, *J* = 5.6 Hz, 2.0 Hz 2H), 5.11 (dt, *J* = 8.4 Hz, 5.6 Hz, 1H), 6.46 (dd, *J* = 1.6 Hz, 3.2 Hz, 1H), 6.46 (dd, *J* = 1.6 Hz, 3.2 Hz, 1H), 6.89 (d, *J* = 7.6 Hz, 1H), 7.01 (d, *J* = 2.4 Hz, 1H), 7.07 (t, *J* = 7.6 Hz, 1H), 7.10 (d, *J* = 3.2 Hz, 1H), 7.18 (t, *J* = 7.6 Hz, 1H), 7.33–7.38 (m, 2H), 7.54 (d, *J* = 7.6 Hz, 1H), 8.24 (brs, 1H); ^13^C-NMR (100 MHz, CDCl_3_) δ 28.1, 52.6, 52.7, 109.9, 111.4, 112.2, 114.8, 118.7, 119.7, 122.3, 123.0, 127.6, 136.3, 144.3, 147.5, 158.0, 172.2; HRMS (EI) HRMS (FAB) Calcd for C_17_H_17_N_2_O_4_ [M + H]^+^: 313.1188, Found: 313.1163.

*(R)-Methyl (furan-2-carbonyl)tryptophanate* (**4**) was synthesized from furan-2-carboxylic acid and d-tryptophan methyl ester hydrochloride; yield 69%; white solid; ^1^H-NMR (400 MHz, CDCl_3_) δ 3.410 (d, *J* = 5.6 Hz, 1H), 3.414 (d, *J* = 5.2 Hz, 1H), 3.69 (s, 3H), 5.11 (dt, *J* = 8.0 Hz, 5.6 Hz, 1H), 6.46 (dd, *J* = 1.6 Hz, 3.2 Hz, 1H), 6.47 (dd, *J* = 1.2 Hz, 3.6 Hz, 1H), 6.89 (d, *J* = 7.6 Hz, 1H), 7.02 (d, *J* = 2.4 Hz, 1H), 7.08 (t, *J* = 7.6 Hz, 1H), 7.10 (d, *J* = 4.0 Hz, 1H), 7.17 (t, *J* = 7.6 Hz, 1H), 7.32–7.37 (m, 2H), 7.54 (d, *J* = 7.6 Hz, 1H), 8.29 (s, 1H); ^13^C-NMR (100 MHz, CDCl_3_) δ 27.9, 52.4, 52.5, 109.9, 111.2, 112.1, 114.7, 118.6, 119.6, 122.2, 122.8, 127.5, 136.1, 144.2, 147.4, 157.9, 172.1; HRMS (FAB) Calcd for C_17_H_17_N_2_O_4_ [M + H]^+^: 313.1188, Found: 313.1189.

*(S)-Methyl (thiophene-2-carbonyl)tryptophanate* (**5**) was synthesized from furan-2-carboxylic acid and l-tryptophan methyl ester hydrochloride; yield 68%; white solid; ^1^H-NMR (300 MHz, CDCl_3_) δ 3.42 (d, *J* = 5.2 Hz, 2H), 3.70 (s, 3H), 5.11 (dt, *J* = 7.2 Hz, 5.2 Hz, 1H), 6.55 (d, 8.0 Hz, 1H), 6.98–7.01 (m, 2H), 7.07–7.11 (m, 1H), 7.16–7.20 (m, 1H), 7.33–7.36 (m, 2H), 7.45 (dd, *J* = 5.2 Hz, 1.2 Hz, 1H), 7.55 (d, *J* = 8.4 Hz, 1H), 8.31 (br, 1H); ^13^C-NMR (100 MHz, CDCl_3_) δ 27.7, 52.4, 53.4, 109.8, 111.3, 118.6, 119.7, 122.3, 122.9, 127.6, 128.4, 130.4, 136.1, 138.3, 161.4, 172.2; HRMS (FAB) Calcd for C_17_H_17_N_2_O_3_S [M + H]^+^: 329.0960, Found: 329.0944.

*(R)-Methyl (thiophene-2-carbonyl)tryptophanate* (**6**) was synthesized from thiophene-2-carboxylic acid and d-tryptophan methyl ester hydrochloride; yield 43%; white solid; ^1^H-NMR (400 MHz, CDCl_3_) δ 3.42 (d, *J* = 5.2 Hz, 2H), 3.70 (s, 3H), 5.12 (dt, *J* = 8.4 Hz, 5.2 Hz, 1H), 6.54 (d, *J* = 8.0 Hz, 1H), 6.98–7.02 (m, 2H), 7.09 (t, *J* = 7.2 Hz, 1H), 7.19 (t, *J* = 7.2 Hz, 1H), 7.32–7.37 (m, 2H), 7.45 (d, *J* = 5.2 Hz, 1H), 7.55 (d, *J* = 7.6 Hz, 1H), 8.30 (br, 1H); ^13^C-NMR (100 MHz, CDCl_3_) δ 27.8, 52.6, 53.5, 109.9, 111.5, 118.7, 119.9, 122.4, 123.0, 127.70, 127.73, 128.6, 130.5, 136.2, 138.4, 161.6, 172.3; HRMS (FAB) Calcd for C_17_H_17_N_2_O_4_S [M + H]^+^: 329.0960, Found: 329.0973.

*(S)-Methyl (5-methylfuran-2-carbonyl)phenylalaninate* (**7**) was synthesized from 5-methylthiophene-2-carboxylic acid and l-phenylalanine methyl ester hydrochloride; yield 84%; pale yellow oil; ^1^H NMR (400 MHz, CDCl_3_) δ 2.33 (s, 3H), 3.18–3.28 (m, 2H), 3.74 (s, 3H), 5.05 (dt, *J* = 8.4 Hz, 5.2 Hz, 1H), 6.09 (br, 1H), 6.70 (d, *J* = 8.0 Hz, 1H), 7.01 (d, *J* = 3.2 Hz, 1H), 7.13–7.18 (m, 2H), 7.23–7.33 (m, 3H); ^13^C-NMR (100 MHz, CDCl) δ 14.0, 38.3, 52.5, 52.9, 108.7, 116.1, 127.2, 128.7, 129.4, 135.9, 145.9, 155.0, 158.0, 172.0; HRMS (FAB) Calcd for C_16_H_18_NO_4_ [M + H]^+^: 288.1236, Found: 288.1246.

*(R)-Methyl (5-methylfuran-2-carbonyl)phenylalaninate* (**8**) was synthesized from 5-methylfuran-2-carboxylic acid and d-phenylalanine methyl ester hydrochloride; yield 81%; pale yellow oil; ^1^H-NMR (400 MHz, CDCl_3_) δ 2.33 (s, 3H), 3.18–3.28 (m, 2H), 3.74 (s, 3H), 5.05 (dt, *J* = 8.4 Hz, 5.2 Hz, 1H), 6.09 (br, 1H), 6.70 (d, *J* = 8.0 Hz, 1H), 7.01 (d, *J* = 3.2 Hz, 1H), 7.13–7.18 (m, 2H), 7.23–7.33 (m, 3H); ^13^C-NMR (100 MHz, CDCl_3_) δ 14.0, 38.3, 52.5, 52.9, 108.7, 116.1, 127.2, 128.7, 129.4, 135.9, 145.9, 155.0, 158.0, 172.0; HRMS (FAB) Calcd for C_16_H_18_NO_4_ [M + H]^+^: 288.1236, Found: 288.1258.

*(S)-Methyl (5-methylfuran-2-carbonyl)tryptophanate* (**9***)* was synthesized from 5-methylfuran-2-carboxylic acid and l-tryptophan methyl ester hydrochloride; yield 78%; white solid; ^1^H-NMR (400 MHz, CDCl_3_) δ 2.26 (s, 3H), 3.35–3.45 (m, 2H), 3.69 (s, 3H), 5.11 (dt, *J* = 8.0 Hz, 5.2 Hz, 1H), 6.06 (br, 1H), 6.78 (d, *J* = 8.4 Hz, 1H), 6.97–7.04 (m, 2H), 7.09 (t, *J* = 8.0 Hz, 1H), 7.18 (t, *J* = 8.0 Hz, 1H), 7.35 (d, *J* = 8.4 Hz, 1H), 7.56 (d, *J* = 8.0 Hz, 1H), 8.21 (br, 1H); ^13^C-NMR (100 MHz, CDCl_3_) δ 13.9, 28.1, 52.5, 52.6, 108.6, 110.2, 111.3, 116.0, 118.8, 119.7, 122.3, 122.9, 127.7, 136.2, 145.9, 155.0, 158.1, 172.4; HRMS (FAB) Calcd for C_18_H_19_N_2_O_4_ [M + H]^+^: 327.1345, Found: 327.1331.

*(R)-Methyl (5-methylfuran-2-carbonyl)tryptophanate* (**10**) was synthesized from 5-methylfuran-2-carboxylic acid and d-tryptophan methyl ester hydrochloride; yield 81%; white solid; ^1^H-NMR (400 MHz, CDCl_3_) δ 2.26 (s, 3H), 3.35–3.45 (m, 2H), 3.69 (s, 3H), 5.11 (dt, *J* = 8.0 Hz, 5.2 Hz, 1H), 6.06 (br, 1H), 6.78 (d, *J* = 8.4 Hz, 1H), 6.97–7.04 (m, 2H), 7.09 (t, *J* = 8.0 Hz, 1H), 7.18 (t, *J* = 8.0 Hz, 1H), 7.35 (d, *J* = 8.4 Hz, 1H), 7.56 (d, *J* = 8.0 Hz, 1H), 8.14 (br, 1H); ^13^C-NMR (100 MHz, CDCl_3_) δ 13.9, 28.1, 52.5, 52.6, 108.6, 110.2, 111.3, 116.0, 118.8, 119.7, 122.3, 122.9, 127.7, 136.2, 145.9, 155.0, 158.1, 172.4; HRMS (FAB) Calcd for C_18_H_19_N_2_O_4_ [M + H]^+^: 327.1345, Found: 327.1362.

*(5-Methylthiophene-2-carbonyl)glycine* (**11**) was synthesized by hydrolysis of methyl (5-methylthiophene-2-carbonyl)glycinate; yield 36%; white solid; ^1^H-NMR (400 MHz, DMSO) δ 2.49 (s, 3H), 3.94 (d, *J* = 6.0 Hz, 2H), 6.87 (dd, *J* = 3.6 Hz, 1.2 Hz, 1H), 7.64 (d, *J* = 3.6 Hz, 1H), 8.75 (t, *J* = 6.0 Hz, 1H); ^13^C-NMR (100 MHz, DMSO) δ 15.4, 41.2, 126.6, 128.9, 136.9, 145.2, 161.8, 171.8; HRMS (FAB) Calcd for C_8_H_10_NO_3_S [M + H]^+^: 200.0381, Found: 200.0379.

*(S)-(5-Methylthiophene-2-carbonyl)alanine* (**12**) was synthesized by hydrolysis of **13**; yield 74%, white solid; ^1^H-NMR (400 MHz, DMSO) δ 1.41 (d, *J* = 7.2 Hz, 1H), 2.55 (t, *J* = 2.0 Hz, 3H), 4.32 (quint, *J* = 7.2 Hz, 1H), 6.89 (dd, *J* = 3.6 Hz, 0.8 Hz, 1H), 7.70 (d, *J* = 3.6 Hz, 1H), 8.59 (d, *J* = 7.6 Hz, 1H); ^13^C-NMR (100 MHz, DMSO) δ 15.8, 17.5, 48.5, 127.0, 129.4, 137.5, 145.5, 161.6, 174.8; LRMS (FAB) *m*/*z* = 214 ([M + H]^+^). 

*(S)-Ethyl (5-methylthiophene-2-carbonyl)alaninate* (**13**) was synthesized from 5-methylthiophene-2-carboxylic acid and l-alanine ethyl ester hydrochloride; yield 65%; white solid; ^1^H-NMR (400 MHz, CDCl_3_) δ 1.30 (t, *J* = 4.8 Hz, 3H), 1.49 (d, *J* = 6.8 Hz, 3H), 2.50 (d, *J* = 0.8 Hz, 3H), 4.23 (q, *J* = 7.2 Hz, 2H), 4.73 (quint, *J* = 7.2 Hz, 1H), 6.56 (d, *J* = 6.4 Hz, 1H), 6.73 (dd, *J* = 3.6 Hz, 1.2 Hz, 1H), 7.36 (d, *J* = 4.0 Hz, 1H); ^13^C-NMR (100 MHz, CDCl_3_) δ 14.0, 15.6, 18.6, 48.3, 61.5, 126.0, 128.7, 135.6, 145.5; HRMS (FAB) Calcd for C_11_H_16_NO_3_S [M + H]^+^: 242.0851, Found: 242.0850.

*(R)-Methyl (5-methylthiophene-2-carbonyl)phenylalaninate* (**14**) was synthesized from 5-methylthiophene-2-carboxylic acid and d-phenylalanine methyl ester hydrochloride; yield 83%; pale yellow solid; ^1^H-NMR (400 MHz, CDCl_3_) δ 2.50 (s, 3H), 3.18–3.28 (m, 2H), 3.75 (s, 3H), 5.05 (dt, *J* = 7.6 Hz, 5.6 Hz, 1H), 6.32 (br, 1H), 6.72 (d, *J* = 3.6 Hz, 1H), 7.10–7.15 (m, 2H), 7.23–7.32 (m, 4H); ^13^C-NMR (100 MHz, CDCl_3_) δ 15.8, 38.1, 52.5, 53.4,126.2, 127.3, 128.7, 128.9, 129.4, 135.5, 135.9, 145.9, 161.4, 172.0; HRMS (FAB) Calcd for C_16_H_18_NO_3_S [M + H]^+^: 304.1007, Found: 304.1006.

*(S)-Ethyl (5-methylthiophene-2-carbonyl)phenylalaninate* (**15**) was synthesized from 5-methylthiophene-2-carboxylic acid and l-phenylalanine ethyl ester hydrochloride; yield 37%; white solid; ^1^H-NMR (400 MHz, CDCl_3_) δ 1.26 (t, *J* = 7.6 Hz, 3H), 2.50 (s, 3H), 3.21 (d, *J* = 1.6 Hz, 2H), 4.19 (q, *J* = 7.6 Hz, 2H), 5.00 (dt, *J* = 7.6 Hz, 5.6 Hz, 1H), 6.35 (d, *J* = 7.6 Hz, 1H), 6.71 (d, *J* = 3.6 Hz, 1H), 7.14 (dd, *J* = 8.0 Hz, 1.6 Hz, 2H), 7.24–7.30 (m, 4H); ^13^C-NMR (100 MHz, CDCl_3_) δ 14.1, 15.6, 38.0, 53.3, 61.6, 126.1, 127.1, 128.5, 128.7, 129.4, 135.5, 135.8, 145.7, 161.2, 171.5; HRMS (FAB) Calcd for C_17_H_20_NO_3_S [M + H]^+^: 318.1164, Found: 318.1152.

*(R)-(5-methylthiophene-2-carbonyl)tryptophan* (**16**) was synthesized by hydrolysis of **18**; yield 99%; pale yellow solid; ^1^H-NMR (300 MHz, CDCl_3_) δ 2.48 (s, 3H), 3.47 (t, *J* = 4.8 Hz, 2H), 3.49 (s, 1H), 5.02–5.04 (m, 1H), 6.36 (d, *J* = 6.8 Hz, 1H), 6.67 (d, *J* = 2.9 Hz, 1H), 7.07–7.29 (m, 4H), 7.39 (d, *J* = 8.0 Hz, 1H), 7.63 (d, *J* = 8.3 Hz, 1H), 8.18 (br, 1H); ^13^C-NMR (75 MHz, DMSO–*d*_6_) δ 15.5, 26.9, 53.7, 110.5, 111.6, 118.3, 118.6, 121.2, 123.7, 126.6, 127.3, 129.0, 136.3, 137.0, 145.1, 161.3, 173.7; HRMS (FAB) Calcd for C_8_H_10_NO_3_S [M + H]^+^: 200.0381, Found: 200.0379.

*(S)-Ethyl (5-methylthiophene-2-carbonyl)tryptophanate* (**17**) was synthesized from 5-methylthiophene-2-carboxylic acid and l-phenylalanine ethyl ester hydrochloride; yield 86%; white solid; ^1^H-NMR (300 MHz, CDCl_3_) δ 1.23 (t, *J* = 6.8 Hz, 3H), 2.50 (s, 3H), 3.42 (d, *J* = 5.1 Hz, 2H), 4.15 (m, 2H), 5.08 (m, 1H), 6.42 (d, *J* = 7.8 Hz, 1H), 6.68 (d, *J* = 2.9 Hz, 1H), 7.03–7.27 (m, 4H), 7.36 (d, *J* = 8.1 Hz, 1H), 7.55 (d, *J* = 8.0 Hz, 1H), 8.08 (br, 1H); ^13^C-NMR (100 MHz, CDCl_3_) δ 14.1, 15.6, 27.8, 53.3, 61.6, 110.0, 111.2, 118.7, 119.6, 122.2, 122.9, 126.0, 127.7, 128.8, 135.6, 136.1, 145.7, 161.5, 171.9; HRMS (FAB) Calcd for C_19_H_21_N_2_O_3_S [M + H]^+^: 357.1273, Found: 357.1279.

*(R)-Ethyl (5-methylthiophene-2-carbonyl)tryptophanate* (**18**) was synthesized from 5-methylthiophene-2-carboxylic acid and d-phenylalanine ethyl ester hydrochloride; yield 88%; white solid; ^1^H-NMR (CDCl_3,_ 400 MHz) δ 1.21 (t, *J* = 7.3 Hz, 3H), 2.46 (s, 3H), 3.40 (d, *J* = 5.4 Hz, 2H), 4.13 (m, 2H), 5.07 (dt, *J* = 8.2 Hz, 5.4 Hz, 1H), 6.48 (d, *J* = 7.8 Hz, 1H), 6.65 (d, *J* = 2.9 Hz, 1H), 6.98 (d, *J* = 2.2 Hz, 1H), 7.05–7.26 (m, 3H), 7.33 (d, *J* = 8.1 Hz, 1H), 7.55 (d, *J* = 7.8 Hz, 1H), 8.41 (br, 1H); ^13^C-NMR (75 MHz, CDCl_3_) δ 14.4, 16.0, 28.2, 53.7, 62.0, 110.3, 111.7, 119.1, 120.0, 122.5, 123.4, 126.5, 128.1, 129.2, 136.0, 136.5, 146.1, 161.9, 172.3; HRMS (FAB) Calcd for C_19_H_21_N_2_O_3_S [M + H]^+^: 357.1273, Found: 357.1272.

*(S)-(5-Phenylfuran-2-carbonyl)phenylalanine* (**19**) was synthesized by hydrolysis of **20**; yield 16%; yellow solid; ^1^H-NMR (400 MHz, DMSO) δ 3.11 (dd, *J* = 14.0 Hz, 10.4 Hz, 1H), 3.22 (dd, *J* = 14.0 Hz, 4.4 Hz, 1H), 4.60–4.66 (m, 1H), 7.08 (d, *J* = 3.6 Hz, 1H), 7.17 (d, *J* = 3.6 Hz, 1H), 7.18 (t, *J* = 6.8 Hz, 1H), 7.26 (t, *J* = 4.0 Hz, 2H), 7.31 (d, *J* = 6.8 Hz, 2H), 7.38 (t, *J* = 7.2 Hz, 1H), 7.48 (t, *J* = 4.0 Hz, 2H), 7.90 (d, *J* = 7.2 Hz, 2H), 8.67 (d, *J* = 8.4 Hz, 1H); ^13^C-NMR (100 MHz, CDCl_3_) δ 36.2, 53.3, 107.6, 116.0, 124.3, 126.4, 128.2, 128.6, 128.9, 129.0, 129.3, 138.0, 146.5, 154.6, 157.5, 172.9; HRMS (FAB) Calcd for C_8_H_10_NO_3_S [M + H]^+^: 200.0381, Found: 200.0379.

*(S)-Methyl (5-phenylfuran-2-carbonyl)phenylalaninate* (**20**) was synthesized from 5-phenylfuran-2-carboxylic acid and l-phenylalanine methyl ester hydrochloride; yield 11%; colorless oil; ^1^H-NMR (400 MHz, CDCl_3_) δ 3.25 (d, *J* = 7.8 Hz, 2H), 3.76 (s, 3H), 5.04–5.10 (m, 1H), 6.72 (d, *J* = 4.9 Hz, 1H), 6.81 (d, *J* = 10.2 Hz, 1H), 7.15–7.46 (m, 9H), 7.66 (d, *J* = 9.8 Hz, 2H).

*(S)-(5-Phenylthiophene-2-carbonyl)alanine* (**21**) was synthesized by hydrolysis of **22**; yield 72%; pale yellow solid; ^1^H-NMR (400 MHz, DMSO) δ 1.40 (d, *J* = 3.6 Hz, 3H), 4.40 (quint, *J* = 7.2 Hz, 1H), 7.37 (t, *J* = 7.2 Hz, 1H), 7.45 (t, *J* = 7.6 Hz, 2H), 7.56 (d, *J* = 3.6 Hz, 1H), 7.71 (d, *J* = 7.2 Hz, 2H), 7.87 (d, *J* = 3.6 Hz, 1H); ^13^C-NMR (100 MHz, DMSO) δ 16.9, 48.1, 124.3, 125.7, 128.5, 129.2, 129.7, 133.1, 138.3, 147.6, 160.8, 174.1; HRMS (FAB) Calcd for C_14_H_14_NO_3_S [M + H]^+^: 276.0694, Found: 276.0701.

*(S)-Ethyl (5-phenylthiophene-2-carbonyl)alaninate* (**22**) was synthesized from 5-phenylthiophene-2-carboxylic acid and l-alanine ethyl ester hydrochloride; yield 53%; white solid; ^1^H-NMR (400 MHz, CDCl_3_) δ 1.32 (t, *J* = 7.1 Hz, 3H), 1.55 (m, 3H), 4.26 (q, *J* = 7.0 Hz, 2H), 4.76 (m, 1H), 6.56 (br, 1H), 7.19–7.43 (m, 4H), 7.51 (d, *J* = 7.4 Hz, 1H), 7.63 (m, 2H).

*(S)-(5-Phenylthiophene-2-carbonyl)phenylalanine* (**23**) was synthesized by hydrolysis of **24**; yield 70%; pale yellow solid; ^1^H-NMR (400 MHz, DMSO) δ 3.05 (dd, *J* = 14.0 Hz, 10.8 Hz, 1H), 3.19 (dd, *J* = 14.0 Hz, 4.4 Hz, 1H), 4.59 (ddd, *J* = 11.2 Hz, 8.8 Hz, 4.4 Hz, 1H), 7.18 (dt, *J* = 7.2 Hz, 1.6 Hz, 1H), 7.27 (t, *J* = 7.6 Hz, 2H), 7.31 (d, *J* = 6.8 Hz, 2H), 7.44 (t, *J* = 7.6 Hz, 2H), 7.53 (d, *J* = 3.6 Hz, 1H), 7.69 (dt, *J* = 6.8 Hz, 1.6 Hz, 2H), 7.82 (d, *J* = 4.0 Hz, 1H); ^13^C-NMR (100 MHz, DMSO) δ 36.3, 54.1, 124.3, 125.7, 126.4, 128.2, 128.5, 129.0, 129.2, 129.6, 133.0, 138.2, 147.7, 161.0, 173.0; HRMS (FAB) Calcd for C_20_H_18_NO_3_S [M + H^+^]: 352.1007, Found: 352.1001. 

*(S)-Methyl (5-phenylthiophene-2-carbonyl)phenylalaninate* (**24**) was synthesized from 5-phenylthiophene-2-carboxylic acid and l-phenylalanine methyl ester hydrochloride; yield 61%; white solid; ^1^H-NMR (400 MHz, CDCl_3_) δ 3.25 (t, *J* = 6.8 Hz, 2H), 3.77 (s, 3H), 5.07 (dt, *J* = 10.2 Hz, 5.3 Hz, 1H), 6.39(d, *J* = 10.2 Hz, 1H), 7.14 (m, 2H), 7.24–7.44 (m, 8H), 7.62 (m, 2H).

*(S)-Methyl (5-phenylthiophene-2-carbonyl)tryptophanate* (**25**) was synthesized from 5-phenylthiophene-2-carboxylic acid and l-tryptophan methyl ester hydrochloride; yield 41%; pale yellow solid; ^1^H-NMR (400 MHz, CDCl_3_) δ 3.44 (d, *J* = 5.2 Hz, 2H), 3.72 (s, 3H), 5.13 (dt, *J* = 8.0 Hz, 4.8 Hz, 1H), 6.50 (d, *J* = 8.0 Hz, 1H), 7.03 (d, *J* = 2.4 Hz, 1H), 7.11 (t, *J* = 7.6 Hz, 1H), 7.16–7.22 (m, 2H), 7.30–7.42 (m, 5H), 7.55–7.61 (m, 3H), 8.14 (br, 1H); ^13^C-NMR (100 MHz, CDCl_3_) δ 27.9, 52.6, 53.5, 110.1, 111.4, 118.8, 119.9, 122.5, 123.0, 123.5, 126.2, 127.7, 128.7, 129.1, 129.5, 133.5, 136.2, 136.4, 149.4, 161.4, 172.3; HRMS (FAB) Calcd for C_23_H_21_N_2_O_3_S [M + H]^+^: 405.1273, Found: 405.1288.

*Methyl (4-phenylthiophene-2-carbonyl)glycinate* (**26**) was synthesized from 4-phenylthiophene-2-carboxylic acid and glycine methyl ester hydrochloride; yield 75%; white powder; mp. 93–96 °C; ^1^H-NMR (400 MHz, CDCl_3_) δ 3.81 (s, 3H), 4.25 (d, *J* = 5.5 Hz, 2H), 6.65 (br, 1H), 7.31 (t, *J* = 7.8 Hz, 1H), 7.40 (t, *J* = 7.8 Hz, 2H), 7.56 (d, *J* = 7.7 Hz, 2H), 7.58 (d, *J* = 1.4 Hz, 1H), 7.84 (d, *J* = 1.4 Hz, 1H); ^13^C-NMR (100 MHz, CDCl_3_) δ 41.7, 52.7, 125.1, 126.4, 127.7, 127.8, 128.7, 129.1, 135.0, 138.5, 143.1, 161.9, 170.5; HRMS (FAB) Calcd for C_14_H_14_NO_3_S [M + H]^+^: 276.0694, Found: 276.0701.

*(S)-Ethyl (4-phenylthiophene-2-carbonyl)phenylalaninate* (**27**) was synthesized from 4-phenylthiophene-2-carboxylic acid and l-phenylalanine ethyl ester hydrochloride; yield 70%; white powder; mp. 94–96 °C; ^1^H-NMR (400 MHz, CDCl_3_) δ 1.20 (t, *J* = 7.0 Hz, 3H), 3.16–3.27 (m, 2H), 4.15 (q, *J* = 7.0 Hz, 2H), 5.01–5.06 (m, 1H), 6.99 (d, *J* = 7.8 Hz, 1H), 7.18 (t, *J* = 7.3 Hz, 2H), 7.19–7.22 (m, 1H), 7.21–7.25 (m, 2H), 7.23–7.27 (m, 1H), 7.33 (t, *J* = 7.3 Hz, 2H), 7.59 (d, *J* = 7.3 Hz, 2H), 7.59 (d, *J* = 1.4 Hz, 1H), 7.95 (d, *J* = 1.4 Hz, 1H); ^13^C-NMR (100 MHz, CDCl_3_) δ 14.2, 38.1, 53.6, 61.8, 125.0, 126.4, 127.3, 127.5, 127.8, 128.7, 129.1, 129.5, 135.0, 135.8, 138.9, 143.1; HRMS (FAB) Calcd for C_22_H_22_NO_3_S [M + H]^+^: 380.1320, Found: 380.1333.

*(S)-Methyl (4-phenylthiophene-2-carbonyl)tryptophanate* (**28**) was synthesized from 4-phenylthiophene-2-carboxylic acid and l-tryptophan methyl ester hydrochloride; yield 69%; flesh color powder; mp. 150–152 °C; ^1^H-NMR (400 MHz, CDCl_3_) δ 3.43 (dd, *J* = 5.0 Hz, 2H), 3.70 (s, 3H), 5.12 (dt, *J* = 8.2 Hz, 5.0 Hz, 1H), 6.62 (d, *J* = 8.2 Hz, 1H), 6.99 (d, *J* = 2.3 Hz, 1H), 7.10 (dt, *J* = 6.8 Hz, 0.9 Hz, 1H), 7.19 (dt, *J* = 6.8 Hz, 1.4 Hz, 1H), 7.30 (dt, *J* = 6.8 Hz, 0.9 Hz, 1H), 7.35–7.39 (m, 3H), 7.46 (dd, *J* = 6.8 Hz, 1.4 Hz, 1H), 7.46 (d, *J* = 8.2 Hz, 1H), 7.53 (dd, *J* = 9.5 Hz, 1.4 Hz, 2H), 7.58 (d, *J* = 8.2 Hz, 1H), 8.43 (s, 1H); ^13^C-NMR (100 MHz, CDCl_3_) δ 27.7, 52.6, 53.6, 110.0, 111.5, 118.7, 120.0, 122.5, 123.0, 125.0, 126.4, 127.5, 127.8, 129.0, 134.9, 136.2, 139.0, 142.9, 161.4, 172.2; HRMS (FAB) Calcd for C_23_H_21_N_2_O_3_S [M + H]^+^: 405.1273, Found: 405.1272.

*(R)-Methyl (4-phenylthiophene-2-carbonyl)tryptophanate* (**29**) was synthesized from 4-phenylthiophene-2-carboxylic acid and d-tryptophan methyl ester hydrochloride; yield 80%; pale yellow solid; mp. 145–149 °C; ^1^H-NMR (400 MHz, DMSO-*d*_6_) δ3.18–3.30 (m, 2H), 3.60 (s, 3H), 4.63–4.69 (m, 1H), 6.95 (dd, *J* = 8.2 Hz, 7.3 Hz, 1H), 7.03 (dd, *J* = 8.2 Hz, 7.7 Hz, 1H), 7.18 (d, *J* = 2.3 Hz, 1H), 7.30 (t, *J* = 7.3 Hz, 1H), 7.30 (d, *J* = 7.7 Hz, 1H), 7.42 (t, *J* = 7.7 Hz, 2H), 7.54 (d, *J* = 7.7 Hz, 1H), 7.66 (d, *J* = 7.7 Hz, 1H), 8.06 (d, *J* = 1.4 Hz, 1H), 8.30 (d, *J* = 1.4 Hz, 1H), 8.86 (d, *J* = 7.7 Hz, 1H), 10.8 (s, 1H); ^13^C-NMR (100 MHz, DMSO-*d*_6_) δ 26.9, 52.0, 53.7, 109.7, 111.5, 118.1, 118.4, 121.0, 123.7, 125.8, 125.9, 127.0, 127.4, 127.6, 129.0, 134.5, 136.1, 139.6, 141.7, 161.1, 172.3; HRMS (FAB) Calcd for C_23_H_21_N_2_O_3_S [M + H]^+^: 405.1273, Found: 405.1279.

*(S)-Methyl (4-phenylthiophene-2-carbonyl)tyrosinate* (**30**) was synthesized from 4-phenylthiophene-2-carboxylic acid and l-tyrosine methyl ester hydrochloride; yield 70%; white solid; mp. 176–178 °C; ^1^H-NMR (400 MHz, DMSO-*d*_6_) δ 2.90–3.07 (m, 2H), 3.63 (s, 3H), 4.50–4.60 (m, 1H), 6.65 (d, *J* = 8.2 Hz, 2H), 7.08 (d, *J* = 8.2 Hz, 2H), 7.34 (t, *J* = 7.7 Hz, 1H), 7.47 (t, *J* = 7.7 Hz, 2H), 7.70 (d, *J* = 7.7 Hz, 2H), 8.10 (s, 1H), 8.33 (s, 1H), 8.87 (d, *J* = 8.2 Hz, 1H), 9.23 (br, 1H); ^13^C-NMR (100 MHz, DMSO-*d*_6_) δ 35.7, 52.0, 54.5, 115.1, 125.8, 126.0, 127.3, 127.6, 127.9, 129.1, 130.0, 134.5, 139.6, 141.7, 156.0, 161.0, 172.1; HRMS (FAB) Calcd for C_21_H_20_NO_4_S [M + H]^+^: 382.1113, Found: 382.1110.

*Methyl (5-((1,1′-biphenyl)-4-yl)thiophene-2-carbonyl)glycinate* (**31**) was synthesized from 5-((1,1′-biphenyl)-4-yl)thiophene-2-carboxylic acid and glycine methyl ester hydrochloride; yield 2%; yellow solid; mp. 183–185 °C; ^1^H-NMR (400 MHz, CDCl_3_) δ 3.82 (s, 3H), 4.26 (d, *J* = 5.2 Hz, 2H), 6.48 (t, *J* = 4.8 Hz, 1H), 7.32 (d, *J* = 4.0 Hz, 1H), 7.37 (t, *J* = 7.6 Hz, 1H), 7.46 (t, *J* = 7.6 Hz, 2H), 7.55 (d, *J* = 4.0 Hz, 1H), 7.62 (d, *J* = 8.4 Hz, 2H), 7.64 (d, *J* = 7.6 Hz, 2H), 7.71 (d, *J* = 8.0 Hz, 2H); ^13^C-NMR (100 MHz, CDCl_3_) δ 41.7, 52.6, 123.6, 126.6, 127.0, 127.8, 127.8, 129.0, 129.7, 132.4, 136.5, 140.3, 141.5, 149.2, 161.9, 170.5; HRMS (FAB) Calcd for C_20_H_18_NO_3_S [M + H]^+^: 352.1007, Found: 352.1006.

*(S)-Ethyl (5-((1,1′-biphenyl)-4-yl)thiophene-2-carbonyl)phenylalaninate* (**32**) was synthesized from 5-((1,1′-biphenyl)-4-yl)thiophene-2-carboxylic acid and l-phenylalanine ethyl ester hydrochloride; yield 15%; yellow solid; ^1^H-NMR (400 MHz, CDCl_3_) δ 1.28 (t, *J* = 7.2 Hz, 3H), 3.24 (dd, *J* = 12.0 Hz, 5.2 Hz, 1H), 3.28 (dd, *J* = 12.0 Hz, 6.0 Hz, 1H), 5.05 (dt, *J* = 7.6 Hz, 5.2 Hz, 1H), 6.45 (d, *J* = 7.2 Hz, 1H), 7.16 (d, *J* = 6.4 Hz, 1H), 7.27–7.32 (m, 3H), 7.37 (t, *J* = 7.6 Hz, 1H), 7.45 (d, *J* = 4.8 Hz, 1H), 7.47 (d, *J* = 7.6 Hz, 2H), 7.62 (d, *J* = 7.6 Hz, 2H), 7.64 (d, *J* = 7.6 Hz, 2H), 7.69 (d, *J* = 7.6 Hz, 2H); ^13^C-NMR (100 MHz, CDCl_3_) δ 14.1, 38.1, 53.5, 61.7, 123.5, 126.5, 126.9, 127.2, 127.6, 127.7, 128.6, 128.9, 129.3, 129.4, 132.4, 135.8, 136.8, 140.2, 141.4, 149.0, 161.1, 171.5; HRMS (FAB) Calcd for C_28_H_26_NO_3_S [M + H]^+^: 456.1633, Found: 456.1637.

*Methyl (5-(4-phenoxyphenyl)thiophene-2-carbonyl)glycinate* (**33**) synthesized from 5-(4-phenoxyphenyl)thiophene-2-carboxylic acid and glycine methyl ester hydrochloride; yield 22%; yellow solid; ^1^H-NMR (400 MHz, CDCl_3_) δ 3.81 (s, 3H), 4.24 (d, *J* = 5.2 Hz, 2H), 6.47 (t, *J* = 4.8 Hz, 1H), 7.02 (dt, *J* = 8.8 Hz, 1.6 Hz, 2H), 7.05 (d, *J* = 7.6 Hz, 2H), 7.15 (t, *J* = 7.2 Hz, 1H), 7.20 (d, *J* = 3.6 Hz, 1H), 7.37 (t, *J* = 8.0 Hz, 2H), 7.51 (d, *J* = 4.0 Hz, 1H), 7.58 (dt, *J* = 8.8 Hz, *J* = 1.6 Hz, 2H); ^13^C-NMR (100 MHz, CDCl_3_) δ 41.6, 52.5, 118.9, 119.3, 123.0, 123.8, 127.6, 128.4, 129.6, 129.9, 136.0, 149.0, 156.5, 158.0, 161.8, 170.4; HRMS (FAB) Calcd for C_20_H_18_NO_4_S [M + H]^+^: 368.0957, Found: 368.0962.

*(S)-Methyl (5-(4-phenoxyphenyl)thiophene-2-carbonyl)alaninate* (**34**) was synthesized from 5-(4-phenoxyphenyl)thiophene-2-carboxylic acid and l-alanine methyl ester hydrochloride; yield 18%; pale yellow solid; ^1^H-NMR (400 MHz, CDCl_3_) δ 1.32 (t, *J* = 7.2 Hz, 3H), 1.52 (d, *J* = 6.8 Hz, 3H), 4.25 (q, *J* = 7.2 Hz, 2H), 4.76 (quint, *J* = 7.2 Hz, 1H), 6.61 (d, *J* = 7.2 Hz, 1H), 7.02 (dt, *J* = 8.8 Hz, *J* = 2.4 Hz, 2H), 7.05 (d, *J* = 8.4 Hz, 2H), 7.19 (d, *J* = 4.0 Hz, 1H), 7.37 (tt, *J* = 8.4 Hz, 2.4 Hz, 2H), 7.50 (d, *J* = 3.6 Hz, 1H), 7.57 (dt, *J* = 9.2 Hz, 2.4 Hz, 2H); ^13^C-NMR (100 MHz, CDCl_3_) δ 14.1, 18.7, 48.5, 61.7, 118.9, 119.3, 123.0, 123.8, 127.6, 128.5, 129.3, 129.9, 136.5, 148.8, 156.6, 157.9, 161.2, 173.2; HRMS (FAB) Calcd for C_22_H_22_NO_4_S [M + H]^+^: 396.1270, Found: 396.1245.

*(S)-Ethyl (5-(4-phenoxyphenyl)thiophene-2-carbonyl)phenylalaninate* (**35**) was synthesized from 5-(4-phenoxyphenyl)thiophene-2-carboxylic acid and l-phenylalanine ethyl ester hydrochloride; yield 8%; yellow oil; ^1^H-NMR (400 MHz, CDCl_3_) δ 1.28 (t, *J* = 7.2 Hz, 2H), 3.25 (q, *J* = 7.2 Hz, 2H), 4.21 (q, *J* = 7.2 Hz, 2H), 5.04 (dt, *J* = 7.2 Hz, *J* = 6.0 Hz, 1H), 6.44 (d, *J* = 7.6 Hz, 1H), 7.02 (dt, *J* = 8.8 Hz, 2.4 Hz, 2H), 7.05 (d, *J* = 8.0 Hz, 2H), 7.25–7.31 (m, 3H), 7.37 (t, *J* = 8.4 Hz, 2H), 7.41 (d, *J* = 4.0 Hz, 1H), 7.57 (dt, *J* = 8.4 Hz, 1.6 Hz, 2H); ^13^C-NMR (100 MHz, CDCl_3_) δ 14.1, 38.1, 53.5, 61.7, 119.0, 119.3, 123.0, 123.8, 127.2, 127.6, 128.4, 128.6, 129.4, 129.9, 135.8, 136.3, 148.9, 156.5, 158.0, 161.2, 171.5; HRMS (FAB) Calcd for C_28_H_26_NO_4_S [M + H]^+^: 472.1583, Found: 472.1587.

*(S)-Methyl (5-(4-phenoxyphenyl)thiophene-2-carbonyl)tryptophanate* (**36**) was synthesized from 5-(4-phenoxyphenyl)thiophene-2-carboxylic acid and l-tryptophan methyl ester hydrochloride; yield 79%; white solid; ^1^H-NMR (400 MHz, CDCl_3_) δ 3.43 (d, *J* = 4.8 Hz, 2H), 3.71 (s, 3H), 5.12 (dt, *J* = 8.0 Hz, *J* = 4.8 Hz, 1H), 6.51 (d, *J* = 7.6 Hz, 1H), 6.99 (d, *J* = 8.8 Hz, 2H), 7.00 (s, 1H), 7.04 (dd, *J* = 8.8 Hz, 1.2 Hz, 2H), 7.10 (td, *J* = 7.6 Hz, 1.2 Hz, 1H), 7.10 (d, *J* = 4.0 Hz, 1H), 7.14 (t, *J* = 8.8 Hz, 1H), 7.19 (t, *J* = 7.6 Hz, 1H), 7.27 (d, *J* = 4.4 Hz, 1H), 7.34 (d, *J* = 7.2 Hz, 1H), 7.36 (t, *J* = 7.2 Hz, 2H), 7.53 (dt, *J* = 8.4 Hz, *J* = 1.6 Hz, 2H), 7.57 (d, *J* = 7.6 Hz, 1H), 8.26 (s, 1H); ^13^C-NMR (100 MHz, CDCl_3_) δ 27.7, 52.4, 53.4, 109.9, 111.3, 118.6, 118.9, 119.3, 119.8, 122.3, 122.92, 122.94, 123.8, 127.5, 127.6, 128.4, 129.4, 129.9, 136.1, 136.4, 148.9, 156.5, 157.9, 161.3, 172.2; HRMS (FAB) Calcd for C_29_H_25_N_2_O_4_S [M + H]^+^: 497.1535, Found: 497.1538.

*(S)-Ethyl (benzofuran-2-carbonyl)phenylalaninate* (**37**) was synthesized from benzofuran-2-carboxylic acid and l-phenylalanine ethyl ester hydrochloride; yield 8%; colorless oil; ^1^H-NMR (400 MHz, CDCl_3_) δ 1.27 (t, *J* = 7.2 Hz, 3H), 3.25–3.28 (m, 2H), 4.21 (q, *J* = 7.2 Hz, 2H), 5.08 (dd, *J* = 6.0 Hz, 2.0 Hz, 1H), 7.12 (d, *J* = 8.0 Hz, 1H), 7.18 (d, *J* = 7.6 Hz, 2H), 7.23–7.32 (m, 4H), 7.41 (td, *J* = 8.0 Hz, 1.2 Hz, 1H), 7.47 (d, *J* = 0.8 Hz, 1H), 7.50 (dd, *J* = 8.4 Hz, *J* = 0.8 Hz,), 7.66 (d, *J* = 8.0 Hz, 1H); ^13^C-NMR (100 MHz, CDCl_3_) δ 14.1, 38.2, 53.1, 61.7, 110.8, 111.9, 122.7, 123.7, 127.0, 127.1, 127.5, 128.6, 129.3, 135.7, 148.1, 154.8, 158.2, 171.2; HRMS (FAB) Calcd for C_20_H_20_NO_4_ [M + H]^+^: 338.1392, Found: 338.1393.

*(S)-Methyl (benzofuran-2-carbonyl)tryptophanate* (**38**) was synthesized from benzofuran-2-carboxylic acid and l-tryptophan methyl ester hydrochloride; yield 31%; white solid; ^1^H-NMR (400 MHz, CDCl_3_) δ 3.45 (d, *J* = 5.2 Hz, 2H), 3.71 (s, 3H), 5.16 (dt, *J* = 8.0 Hz, 5.6 Hz, 1H), 7.04 (d, *J* = 2.4 Hz, 1H), 7.08 (t, *J* = 7.6 Hz, 1H), 7.18 (t, *J* = 7.2 Hz, 2H), 7.28 (t, *J* = 7.6 Hz, 1H), 7.35 (d, *J* = 8.4 Hz, 1H), 7.39 (dd, *J* = 6.8 Hz, 1.2 Hz, 1H), 7.41 (d, *J* = 6.8 Hz, 1H), 7.46 (s, 1H); ^13^C-NMR (100 MHz, CDCl_3_) δ 27.9, 52.5, 52.8, 109.8, 110.8, 111.3, 111.9, 119.7, 122.2, 122.7, 123.6, 127.0, 127.4, 127.5, 136.2, 148.2, 154.8, 158.4, 171.9; HRMS (FAB) Calcd for C_21_H_19_N_2_O_4_ [M + H]^+^: 363.1345, Found: 363.1341. 

*Methyl (benzothiophene-2-carbonyl)glycinate* (**39**) was synthesized from benzothiophene-2-carboxylic acid and glycine methyl ester hydrochloride; yield 45%; white solid; ^1^H-NMR (400 MHz, DMSO) δ 3.67 (s, 3H), 4.05 (d, *J* = 6.0 Hz, 2H), 7.44–7.48 (m, 2H), 7.96 (dd, *J* = 6.4 Hz, 1.6 Hz, 1H), 8.03 (dd, *J* = 6.8 Hz, 2.0 Hz, 1H), 8.13 (s, 1H), 9.25 (t, *J* = 2.0 Hz, 1H); ^13^C-NMR (100 MHz, DMSO) δ 41.1, 51.8, 122.8, 125.0, 125.3, 125.4, 126.4, 139.0, 139.1, 140.3, 162.0, 170.2; HRMS (FAB) Calcd for C_12_H_12_NO_3_S [M + H]^+^: 250.0538, Found: 250.0538.

*(S)-Ethyl (benzothiophene-2-carbonyl)alaninate* (**40**) was synthesized from benzothiophene-2-carboxylic acid and l-alanine ethyl ester hydrochloride; yield 50%; white solid; ^1^H-NMR (400 MHz, DMSO) δ 1.20 (t, *J* = 7.6 Hz, 3H), 1.43 (d, *J* = 7.6 Hz, 2H), 4.12 (qd, *J* = 7.6 Hz, 1.2 Hz, 2H), 4.46 (quint, *J* = 7.6 Hz, 1H), 7.42–7.49 (m, 2H), 7.97 (dd, *J* = 6.4 Hz, 2.4 Hz, 1H), 8.02 (dd, *J* = 6.8 Hz, 1.6 Hz, 1H), 8.21 (s, 1H), 9.08 (d, *J* = 6.8 Hz, 1H); ^13^C-NMR (100 MHz, DMSO) δ 14.1, 16.7, 48.4, 60.6, 122.8, 124.9, 125.3, 125.5, 126.3, 139.1, 140.3, 161.5, 172.4; HRMS (FAB) Calcd for C_14_H_16_NO_3_S [M + H]^+^: 278.0851, Found: 278.0871.

*(S)-Ethyl (benzothiophene-2-carbonyl)phenylalaninate* (**41**) was synthesized from benzothiophene-2-carboxylic acid and l-phenylalanine ethyl ester hydrochloride; yield 35%; white solid; ^1^H-NMR (400 MHz, CDCl_3_) δ 1.31 (t, *J* = 7.1 Hz, 3H), 3.25–3.35 (dd, *J* = 8.6 Hz, 5.9 Hz, 2H), 4.23–4.28 (q, *J* = 7.2 Hz, 2H), 5.07–5.12 (q, *J* = 7.3 Hz, 1H), 6.62 (d, *J* = 7.3 Hz, 1H), 7.18 (d, *J* = 7.8 Hz, 2H), 7.29–7.31 (m, 3H), 7.40–7.44 (m, 2H), 7.75 (s, 1H), 7.84 (t, *J* = 9.0 Hz, 2H); ^13^C-NMR (100 MHz, CDCl_3_) δ 14.2, 38.0, 53.6, 61.8, 122.7, 125.0, 125.1, 125.5, 126.5, 127.2, 128.6, 129.5, 135.7, 137.9, 139.0, 141.0, 161.6, 171.3.

*(R)-Ethyl (benzothiophene-2-carbonyl)phenylalaninate* (**42**) was synthesized from benzothiophene-2-carboxylic acid and d-phenylalanine ethyl ester hydrochloride; yield 23%; white solid; ^1^H-NMR (400 MHz, CDCl_3_) δ 3.21–3.33 (m, 2H), 3.77 (s, 3H), 5.09 (dt, *J* = 7.6 Hz, 5.6 Hz, 1H), 6.60 (d, *J* = 7.2 Hz, 1H), 7.15 (dd, *J* = 7.6 Hz, 1.2 Hz, 2H), 7.24–7.32 (m, 3H), 7.37–7.44 (m, 2H), 7.72 (s, 1H), 7.83 (t, *J* = 8.0 Hz, 2H); ^13^C-NMR (100 MHz, CDCl_3_) δ 37.9, 52.5, 53.6, 122.7, 124.9, 125.1, 125.6, 126.5, 127.3, 128.7, 129.3, 135.6, 137.7, 140.0, 141.0, 161.7, 171.7; HRMS (FAB) Calcd for C_19_H_18_NO_3_S [M + H]^+^: 340.1007, Found: 340.1008.

*(S)-Methyl (benzothiophene-2-carbonyl)tryptophanate* (**43**) was synthesized from benzothiophene-2-carboxylic acid and l-tryptophan methyl ester hydrochloride; yield 36%; white solid; ^1^H-NMR (400 MHz, CDCl_3_) δ 3.47 (d, *J* = 4.8 Hz, 2H), 3.73 (s, 3H), 5.15 (dt, *J* = 8.0 Hz, 4.8 Hz, 1H), 6.66 (d, *J* = 8.0 Hz, 1H), 7.03 (d, *J* = 2.4 Hz, 1H), 7.10 (t, *J* = 8.4 Hz, 1H), 7.20 (t, *J* = 6.8 Hz, 1H), 7.36–7.43 (m, 3H), 7.58 (d, *J* = 8.0 Hz, 1H), 7.60 (s, 1H), 7.77 (d, *J* = 6.8 Hz, 1H), 7.84 (d, *J* = 7.6 Hz, 1H), 8.13 (s, 1H); ^13^C-NMR (100 MHz, CDCl_3_) δ 27.7, 52.5, 53.5, 109.9, 111.3, 118.7, 119.9, 122.4, 122.7, 122.9, 124.9, 125.1, 125.5, 126.4, 127.7, 136.1, 137.9, 139.0, 141.0, 161.8, 172.0; HRMS (FAB) Calcd for C_21_H_19_N_2_O_3_S [M + H]^+^: 379.1116, Found: 379.1110.

*(S)-Methyl (benzothiophene-2-carbonyl)tyrosinate* (**44**) was synthesized from benzothiophene-2-carboxylic acid and l-tyrosine methyl ester hydrochloride; yield 19%; white solid; ^1^H-NMR (400 MHz, DMSO) δ 2.98–3.09 (m, 2H), 3.64 (s, 3H), 4.55–4.61 (m, 1H), 6.65 (d, *J* = 8.3 Hz, 2H), 7.08 (d, *J* = 8.7 Hz, 2H), 7.44–7.49 (m, 2H), 7.96–8.02 (m, 2H), 9.09 (d, *J* = 7.5 Hz, 1H), 9.20 (s, 1H); ^13^C-NMR (100 MHz, DMSO) δ 35.5, 52.0, 54.7, 115.1, 122.8, 124.9, 125.3, 125.4, 126.3, 127.4, 130.0, 138.9, 139.0, 140.2, 155.9, 161.6, 172.0; HRMS (FAB) Calcd for C_19_H_18_NO_4_S [M + H]^+^: 356.0957, Found: 356.0956.

### 4.7. Docking Simulation

To calculate the binding affinity between FIH-1 and the identified compounds (compounds **7**, **9**–**11**, **15–18**, **28**, **30**, **38**), we used Molegro Virtual Docker 6.0.0 [44]. We obtained the crystal structure data for FIH-1 (PDB ID: 1YCI [31]) from the RCSB Protein Data Bank. The binding motifs were also predicted and drawn with Molegro Virtual Docker 6.0.0. To determine the binding affinity, we used the MolDock score from MolDock Optimizer using default settings. The number of trial runs for calculations was 20. The chemical structures of the ligands were drawn using ChemBioDraw Ultra 13.0, and optimized by MM2 calculations in Chem3D Pro 13.0.

### 4.8. Calculation of Solubility

The clogP values of each compounds were calculated by ChemBioDraw Ultra 13.0.

### 4.9. Cell Culture

The human neuroblastoma cell line, SK-N-BE(2)c was purchased from American Type Culture Collection (ATCC, Manassas, VA, USA). SKN:HRE-MLuc were stable transformants of SK-N-BE(2)c by a reporter vector possessing secretion-type luciferase (*Metridia* luciferase, MLuc) under controlling 7 times repeated VEGFA regulatory sequence (40 bp length containing hypoxia response element (HRE, indicated with underline, 5′-CCA CAG TGC ATA CGT GGG CTC CAA CAG GTC CTC TGG ATC T-3′) and mini TATA promoter (5′-CGA GCT CTA GAG GGT ATA TAA TGG AAG CTC GAA TTC CAG CTT GGC ATT CCG GTA CTG TTG GTA AA-3′) [41]. SK-N-BE(2)c and SKN:HRE-MLuc were cultured in RPMI1640 medium (Nacalai, Kyoto, Japan) containing 10% charcoal/resin absorbed heat-inactivated fetal bovine serum (FBS, MP Biomedicals, Santa Ana, CA, USA) and antibiotics (100 U/mL penicillin and 100 μg/mL streptomycin) under a humidified atmosphere with 5% CO_2_ at 37 °C.

### 4.10. Evaluation of HIF Activity under Hypoxia Using a Luciferase Assay

SKN:HRE-MLuc (4 × 10^4^) were incubated for 24 h on a 96 well culture plate. The medium was replaced with complete media and the test compounds (100, 25, 6.3 and 1.6 μM). DMSO (0.1%) was used as a vehicle control, DMOG (100 μM) and FG4592 (100 μM) were used as a PHDs or FIH-1 inhibitor. The cells were incubated for 24 h under a humidified atmosphere of 5% CO_2_ and 3% O_2_ at 37 °C. After incubation, the MLuc activity in culture media was measured with luciferase substrate (Takara, Shiga, Japan) by LB960 luminometer (Berthold, Wildbad, Germany). The HIF activity was calculated as the MLuc intensity, based on the vehicle and positive controls. The lower limit dose out of 100 μM, 25 μM, 6.3 μM, 1.6 μM was listed in Table 1 and Table 2, where 25% RLU intensity was measured using DMOG as a positive control.

### 4.11. MTS Assay

Cellular toxicity was measured using the CellTiter 96^®^ Aqueous One Solution Cell Proliferation Assay kit (Promega, Madison, WI, USA). The cells were stimulated with each compound and treated with the CellTiter solution. After incubation for 3 h, the absorbance of the solutions at 492 nm was measured by Multiskan FC (Thermo Fisher Scientific, Waltham, MS, USA). As a negative control, wells with culture media were used. Wells treated with DMSO (0.1%) were used as a positive control. The IC_50_ values were determined using these controls.

### 4.12. Evaluation of HIF Activity under Normoxia Using a Luciferase Assay 

SKN:HRE-MLuc (4 × 10^4^) were incubated for 24 h on a 96 well culture plate. The medium was replaced with complete media and the tested compounds (100 μM). DMSO (0.1%) was used as a negative control, and DMOG (100 μM) was used as a positive control. The cells were incubated for 24 h under a humidified atmosphere of 5% CO_2_ at 37 °C. After incubation, the MLuc activity in culture media was measured with the method descried above.

### 4.13. Silence of FIH-1 by Small Interfering RNA

The siRNA reagents, including the transfection reagents, were obtained from Thermo Fisher Scientific. Sequence: forward 5′-GAA ACA UUG AGA AGA UGC UUG GAG A-3′ and reverse 5′-UCU CCA AGC AUC UUC UCA AUG UUU C-3′. SKN: HRE-MLuc were transfected with siRNA transiently, according to the protocol for Lipofectamine 2000 (Thermo Fisher Scientific) under normoxic conditions. The siRNA-transfectants were incubated for 72 h. To confirm the FIH-1 knockdown effect in cellular lysate was evaluated by immunoblotting after an additional 24 h culture under normoxic condition. Briefly, cell lysate from siRNA transfectants were separated by SDS polyacrylamide gels, transferred to polyvinylidene difluoride membranes. The blots were blocked with 5% skim milk (Nacalai Tesque) in TBS-Tween, and treated with anti-human FIH-1 polyclonal goat antibody (sc-26219, Santa Cruz Biotechnology, Dallas, TX, USA) or anti β-actin mouse monoclonal antibody (Merck) antibody, subsequently the blots were treated with horseradish peroxidase-conjugated anti- goat or -mouse secondary antibody. Immunoreactivity was visualized with LAS400 image analyzer (GE healthcare, Little Chalfont, UK) using the Western Lightning Chemiluminescence Reagent Plus kit (PerkinElmer, Waltham, MS, USA).

### 4.14. Gene Expression Analysis

Total RNA was extracted from cells using ISOGEN (Nippon Gene, Tokyo, Japan). cDNA syntheses were performed using random hexamer primers and SuperScript II Reverse Transcriptase (Thermo Fisher Scientific) following the manufacturer’s instructions. Quantitative reverse transcription polymerase-chain reaction (RT-PCR) analysis was performed as follows: amplification was done in an initial denaturing step at 96 °C for 2 min, followed by 35 cycles of PCR (96 °C for 1 min, 57 °C for 30 s, 72 °C for 30 s) using recombinant Taq polymerase (Takara, Otsu, Japan) with the respective primer pairs. For human carbonic anhydrase IX (CA9), 5′-CCT GGC TGC TGG TGA CAT CC-3′ and 5′-AAG GAA GTG GCA TAA TGA GC-3′. Real time RT-PCR was performed on a LightCycler480 thermal cycler system using a LightCycler480 SYBR Green I Master (Roche Applied Science, Indianapolis, IN) according to the manufacturer’s instructions. Data were analyzed using the comparative Ct method as means of relative quantification, and were normalized to an endogenous reference (β-actin) and relative to a calibrator (normalized Ct value obtained from cells treated with vehicle only). Data were expressed as 2^−ΔΔCt^.

### 4.15. Statistical Analyses

All data were analyzed using one-way ANOVA followed by Newman-Keuls Multiple Comparison Test using GraphPad Prism 6 software (La Jolla, CA, USA).

## Figures and Tables

**Table 1:** Evaluation of HIF activity by several furan- and thiophene-2-carbonyl amino acid derivatives, their toxicity, and their partition coefficients.

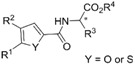											
Entry	Y	R^1^	R^2^	R^3^	R^4^	*R*/*S*	Compound No.	25% Activity ^a^	100 μM Activity ^b^	Toxicity IC_50_^c^	clog*P*
1	O	H	H	H	Me	*-*	**1**	25 μM	20%	>100 μM	0.04
2	S	H	H	H	Me	*-*	**2**	ND	0%	>100 μM	0.70
3	O	H	H	-CH_2_-3-indolyl	Me	*S*	**3**	ND	0%	>100 μM	1.03
4	O	H	H	-CH_2_-3-indolyl	Me	*R*	**4**	ND	0%	>100 μM	1.03
5	S	H	H	-CH_2_-3-indolyl	Me	*S*	**5**	ND	0%	>100 μM	2.39
6	S	H	H	-CH_2_-3-indolyl	Me	*R*	**6**	ND	0%	>100 μM	2.39
7	O	Me	H	-CH_2_-Ph	Me	*S*	**7**	25 μM	22%	>100 μM	1.82
8	O	Me	H	-CH_2_-Ph	Me	*R*	**8**	ND	2%	>100 μM	1.82
9	O	Me	H	-CH_2_-3-indolyl	Me	*S*	**9**	25 μM	22%	>100 μM	1.37
10	O	Me	H	-CH_2_-3-indolyl	Me	*R*	**10**	6.3 μM	0%	>100 μM	1.37
11	S	Me	H	H	H	*-*	**11**	25 μM	23%	>100 μM	0.49
12	S	Me	H	Me	H	*S*	**12**	ND	18%	>100 μM	1.25
13	S	Me	H	Me	Et	*S*	**13**	ND	0%	>100 μM	1.85
14	S	Me	H	-CH_2_-Ph	Me	*R*	**14**	ND	0%	>100 μM	3.19
15	S	Me	H	-CH_2_-Ph	Et	*S*	**15**	25 μM	12%	>100 μM	3.53
16	S	Me	H	-CH_2_-3-indolyl	H	*R*	**16**	100 μM	30%	>100 μM	2.47
17	S	Me	H	-CH_2_-3-indolyl	Et	*S*	**17**	100 μM	49%	>100 μM	3.07
18	S	Me	H	-CH_2_-3-indolyl	Et	*R*	**18**	100 μM	52%	>100 μM	3.07
19	O	Ph	H	-CH_2_-Ph	H	*S*	**19**	ND	0%	>100 μM	2.96
20	O	Ph	H	-CH_2_-Ph	Me	*S*	**20**	ND	10%	>100 μM	3.22
21	S	Ph	H	Me	H	*S*	**21**	ND	0%	>100 μM	2.65
22	S	Ph	H	Me	Et	*S*	**22**	ND	16%	>100 μM	3.25
23	S	Ph	H	-CH_2_-Ph	H	*S*	**23**	ND	0%	>100 μM	4.32
24	S	Ph	H	-CH_2_-Ph	Me	*S*	**24**	ND	7%	>100 μM	4.58
25	S	Ph	H	-CH_2_-3-indolyl	Me	*S*	**25**	ND	0%	>100 μM	4.13
26	S	H	Ph	H	Me	*-*	**26**	ND	0%	>100 μM	2.36
27	S	H	Ph	-CH_2_-Ph	Et	*S*	**27**	ND	2%	>100 μM	4.87
28	S	H	Ph	-CH_2_-3-indolyl	Me	*S*	**28**	25 μM	0%	>100 μM	4.07
29	S	H	Ph	-CH_2_-3-indolyl	Me	*R*	**29**	ND	0%	>100 μM	4.07
30	S	H	Ph	-CH_2_-C_6_H_4_-4-OH	Me	*S*	**30**	6.3 μM	0%	75 μM	4.14
31	S	4-Ph-C_6_H_4_	H	H	Me	*-*	**31**	ND	0%	>100 μM	4.09
32	S	4-Ph-C_6_H_4_	H	-CH_2_-Ph	Et	*S*	**32**	ND	0%	>100 μM	6.60
33	S	4-PhO-C_6_H_4_	H	H	Me	*-*	**33**	ND	0%	>100 μM	3.96
34	S	4-PhO-C_6_H_4_	H	Me	Me	*S*	**34**	ND	0%	>100 μM	4.45
35	S	4-PhO-C_6_H_4_	H	-CH_2_-Ph	Et	*S*	**35**	ND	0%	>100 μM	6.12
36	S	4-PhO-C_6_H_4_	H	-CH_2_-3-indolyl	Me	*S*	**36**	ND	0%	>100 μM	5.66

^a^ 25% activity represents the lower dose limit showing 25% of the RLU intensity of the positive control DMOG; ^b^ 100 μM activity represents the RLU intensity at 100 μM stimulation compared with the positive control DMOG; ^c^ The values were calculated using the MTS assay results. ND = not determined.

**Table 2:** Evaluation of HIF activity by benzofuran- and benzothiophene-2-carbonyl amino acid derivatives, their toxicity, and their partition coefficients.

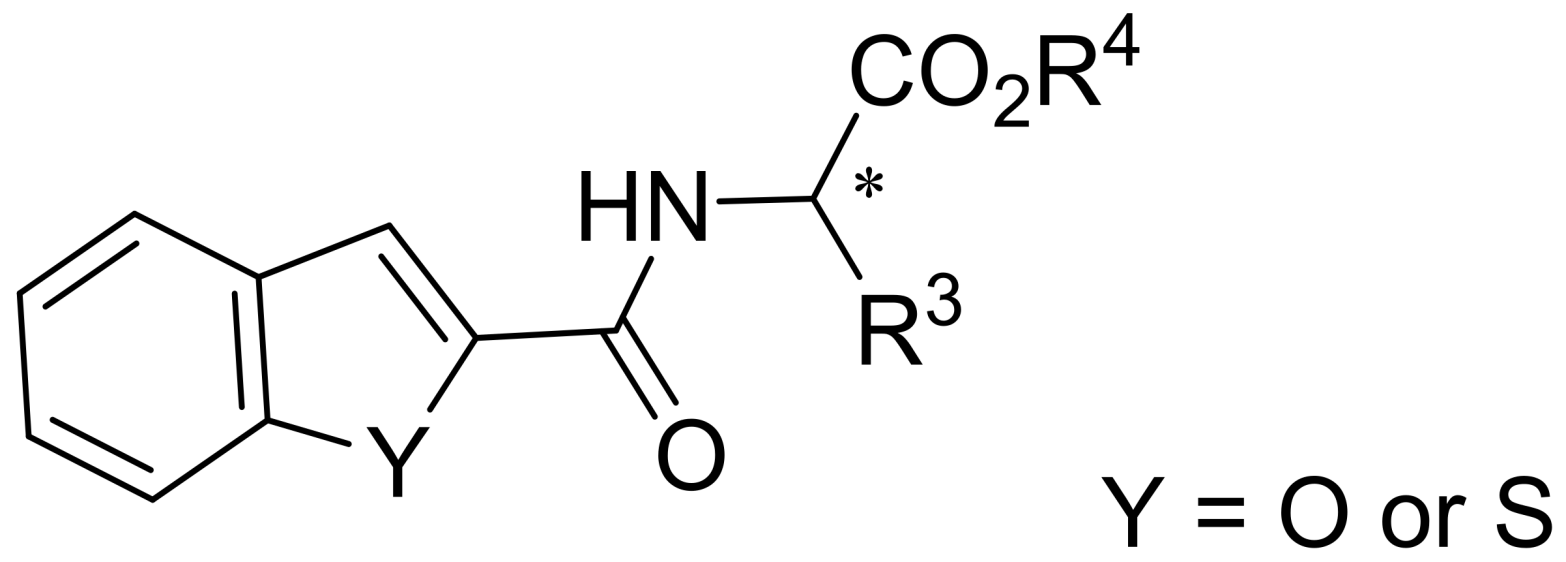									
Entry	Y	R^3^	R^4^	*R*/*S*	Compound No.	25% Activity ^a^	100 μM Activity ^b^	Toxicity IC_50_^c^	clog*P*
37	O	-CH_2_-Ph	Et	*S*	**37**	ND	0%	>100 μM	2.88
38	O	-CH_2_-3-indolyl	Me	*S*	**38**	25 μM	25%	>100 μM	2.08
39	S	H	Me	*-*	**39**	ND	13%	>100 μM	1.74
40	S	Me	Et	*S*	**40**	ND	0%	>100 μM	2.57
41	S	-CH_2_-Ph	Et	*S*	**41**	ND	2%	>100 μM	4.24
42	S	-CH_2_-Ph	Me	*R*	**42**	ND	0%	>100 μM	3.91
43	S	-CH_2_-3-indolyl	Me	*S*	**43**	ND	0%	>100 μM	3.45
44	S	-CH_2_-C_6_H_4_-4-OH	Me	*S*	**44**	ND	0%	>100 μM	3.52

^a^ 25% activity represents the lower dose limit showing 25% of the RLU intensity of the positive control DMOG; ^b^ 100 μM activity represents the RLU intensity at 100 μM stimulation compared with the positive control DMOG; ^c^ The values were calculated using the MTS assay results. ND = not determined.

**Table 3:** The docking scores of FIH-1 with compounds **7**, **9**, **10**, and **16** run on Molegro Virtual Docker 6.0.0.

Entry	Compound	Score (Plants)
1 ^a^	**7** (hydrolysate)	−63.8
2 ^a^	**9** (hydrolysate)	−70.8
3 ^a^	**10** (hydrolysate)	−57.7
4	**16**	−29.9

^a^ Docking simulations of FIH-1 were performed with their carboxylic acid.

## Supplementary Materials

Supplementary File 1

## References

[1] Ozer A., Bruick R.K. (2007). Non-heme dioxygenases: Cellular sensors and regulators jelly rolled into one?. Nat. Chem. Biol..

[2] Fraisl P., Aragones J., Carmeliet P. (2009). Inhibition of oxygen sensors as a therapeutic strategy for ischaemic and inflammatory disease. Nat. Rev. Drug Discov..

[3] Rabinowitz M.H. (2013). Inhibition of hypoxia-inducible factor prolyl hydroxylase domain oxygen sensors: Tricking the body into mounting orchestrated survival and repair responses. J. Med. Chem..

[4] Miyata T., de Strihou C.V. (2010). Diabetic nephropathy: A disorder of oxygen metabolism?. Nat. Rev. Nephrol..

[5] Miyata T., Suzuki N., de Strihou C.V. (2013). Diabetic nephropathy: Are there new and potentially promising therapies targeting oxygen biology?. Kidney Int..

[6] Ke Q., Costa M. (2006). Hypoxia-inducible factor-1 (HIF-1). Mol. Pharmacol..

[7] Chan M.C., Holt-Martyn J.P., Schofield C.J., Ratcliffe P.J. (2016). Pharmacological targeting of the HIF hydroxylases—A new field in medicine development. Mol. Asp. Med..

[8] Gupta N., Wish J.B. (2017). Hypoxia-inducible factor prolyl hydroxylase inhibitors: A potential new treatment for anemia in patients with CKD. Am. J. Kidney Dis..

[9] Lando D., Peet D.J., Whelan D.A., Gorman J.J., Whitelaw M.L. (2002). Asparagine hydroxylation of the HIF transactivation domain a hypoxic switch. Science.

[10] Stolze I.P., Tian Y.M., Appelhoff R.J., Turley H., Wykoff C.C., Gleadle J.M., Ratcliffe P.J. (2004). Genetic analysis of the role of the asparaginyl hydroxylase factor inhibiting hypoxia-inducible factor (HIF) in regulating HIF transcriptional target genes. J. Biol. Chem..

[11] Hirota K., Semenza G.L. (2005). Regulation of hypoxia-inducible factor 1 by prolyl and asparaginyl hydroxylases. Biochem. Biophys. Res. Commun..

[12] Mahon P.C., Hirota K., Semenza G.L. (2001). FIH-1: A novel protein that interacts with HIF-1α and VHL to mediate repression of HIF-1 transcriptional activity. Genes Dev..

[13] Barrett T.D., Palomino H.L., Brondstetter T.I., Kanelakis K.C., Wu X., Haug P.V., Yan W., Young A., Hua H., Hart J.C. (2011). Pharmacological characterization of 1-(5-chloro-6-(trifluoromethoxy)-1H-benzoimidazol-2-yl)-1H-pyrazole-4-carboxylic acid (JNJ-42041935), a potent and selective hypoxia-inducible factor prolyl hydroxylase inhibitor. Mol. Pharmacol..

[14] Vachal P., Miao S., Pierce J.M., Guiadeen D., Colandrea V.J., Wyvratt M.J., Salowe S.P., Sonatore L.M., Milligan J.A., Hajdu R. (2012). 1,3,8-Triazaspiro[4,5]decane-2,4-diones as efficacious pan-inhibitors of hypoxia-inducible factor prolyl hydroxylase 1–3 (HIF PHD1–3) for the treatment of anemia. J. Med. Chem..

[15] Pergola P.E., Spinowitz B.S., Hartman C.S., Maroni B.J., Haase V.H. (2016). Vadadustat, A novel oral HIF stabilizer, provides effective anemia treatment in nondialysis-dependent chronic kidney disease. Kidney Int..

[16] Thevis M., Milosovich S., Licea-Perez H., Knecht D., Cavalier T., Schänzer W. (2016). Mass spectrometric characterization of a prolyl hydroxylase inhibitor GSK1278863, its bishydroxylated metabolite, and its implementation into routine doping controls. Drug Test. Anal..

[17] Eichner D., Van Wagoner R.M., Brenner M., Chou J., Leigh S., Wright L.R., Flippin L.A., Martinelli M., Krug O., Schänzer W. (2017). Lmplementation of the prolyl hydroxylase inhibitor roxadustat (FG-4592) and its main metabolites into routine doping controls. Drug Test. Anal..

[18] Mole D.R., Schlemminger I., McNeill L.A., Hewitson K.S., Pugh C.W., Ratcliffe P.J., Schofield C.J. (2003). 2-Oxoglutarate analogue inhibitors of HIF prolyl hydroxylase. Bioorg. Med. Chem. Lett..

[19] Mecinovic J., Loenarz C., Chowdhury R., Schofield C.J. (2009). 2-Oxoglutarate analogue inhibitors of prolyl hydroxylase domain 2. Bioorg. Med. Chem. Lett..

[20] Murray J.K., Balan C., Allgeier A.M., Kasparian A., Viswanadhan V., Wilde C., Allen J.R., Yoder S.C., Biddlecome G., Hungate R.W. (2010). Dipeptidyl-quinolone derivatives inhibit hypoxia inducible factor-1 alpha prolyl hydroxylases-1, -2, and -3 with altered selectivity. J. Comb. Chem..

[21] Kwon H.S., Choi Y.K., Kim J.W., Park Y.K., Yang E.G., Ahn D.R. (2011). Inhibition of a prolyl hydroxylase domain (PHD) by substrate analog peptides. Bioorg. Med. Chem. Lett..

[22] Nangaku M., Izuhara Y., Takizawa S., Yamashita T., Fujii-Kuriyama Y., Ohneda O., Yamamoto M., de Strihou C.V., Hirayama N., Miyata T. (2007). A novel class of prolyl hydroxylase inhibitors induces angiogenesis and exerts organ protection against ischemia. Arterioscler. Thromb. Vasc. Biol..

[23] Rosen M.D., Venkatesan H., Peltier H.M., Bembenek S.D., Kanelakis K.C., Zhao L.X., Leonard B.E., Hocutt F.M., Wu X.D., Palomino H.L. (2010). Benzimidazole-2-pyrazole HIF prolyl 4-hydroxylase inhibitors as oral erythropoietin secretagogues. ACS Med. Chem. Lett..

[24] Ivan M., Haberberger T., Gervasi D.C., Michelson K.S., Gunzler V., Kondo K., Yang H., Sorokina I., Conaway R.C., Conaway J.W. (2002). Biochemical purification and pharmacological inhibition of a mammalian prolyl hydroxylase acting on hypoxia-inducible factor. Proc. Natl. Acad. Sci. USA.

[25] Warshakoon N.C., Wu S., Boyer A., Kawamoto R., Sheville J., Renock S., Xu K., Pokross M., Zhou S., Winter C. (2006). Structure-based design, synthesis, and SAR evaluation of a new series of 8-hydroxyquinolines as HIF-1α prolyl hydroxylase inhibitors. Bioorg. Med. Chem. Lett..

[26] Frohn M., Viswanadhan V., Pickrell A.J., Golden J.E., Muller K.M., Burli R.W., Biddlecome G., Yoder S.C., Rogers N., Dao J.H. (2008). Structure-guided design of substituted aza-benzimidazoles as potent hypoxia inducible factor-1alpha prolyl hydroxylase-2 inhibitors. Bioorg. Med. Chem. Lett..

[27] McDonough M.A., Li V., Flashman E., Chowdhury R., Mohr C., Lienard B.M., Zondlo J., Oldham N.J., Clifton I.J., Lewis J. (2006). Cellular oxygen sensing: Crystal structure of hypoxia-inducible factor prolyl hydroxylase (PHD2). Proc. Natl. Acad. Sci. USA.

[28] Hong Y.R., Kim H.T., Lee S.C., Ro S., Cho J.M., Kim I.S., Jung Y.H. (2013). [(4-Hydroxyl-benzo[4,5]thieno[3,2-c]pyridine-3-carbonyl)-amino]-acetic acid derivatives; HIF prolyl 4-hydroxylase inhibitors as oral erythropoietin secretagogues. Bioorg. Med. Chem. Lett..

[29] Zhang N., Fu Z., Linke S., Chicher J., Gorman J.J., Visk D., Haddad G.G., Poellinger L., Peet D.J., Powell F. (2010). The asparaginyl hydroxylase factor inhibiting HIF-1α is an essential regulator of metabolism. Cell Metab..

[30] Dayan F., Roux D., Brahimi-Horn M.C., Pouyssegur J., Mazure N.M. (2006). The oxygen sensor factor-inhibiting hypoxia-inducible factor-1 controls expression of distinct genes through the bifunctional transcriptional character of hypoxia-inducible factor-1α. Cancer Res..

[31] McDonough M.A., McNeill L.A., Tilliet M., Papamicaël C.A., Chen Q.-Y., Banerji B., Hewitson K.S., Schofield C.J. (2005). Selective inhibition of factor inhibiting hypoxia-inducible factor. J. Am. Chem. Soc..

[32] Banerji B., Conejo-Garcia A., McNeill L.A., McDonough M.A., Buck M.R.G., Hewitson K.S., Oldham N.J., Schofield C.J. (2005). The inhibition of factor inhibiting hypoxia-inducible factor (FIH) by β-oxocarboxylic acids. Chem. Commun..

[33] Conejo-Garcia A., McDonough M.A., Loenarz C., McNeill L.A., Hewitson K.S., Ge W., Liénard B.M., Schofield C.J., Clifton I.J. (2010). Structural basis for binding of cyclic 2-oxoglutarate analogues to factor-inhibiting hypoxia-inducible factor. Bioorg. Med. Chem. Lett..

[34] Chan M.C., Ilott N.E., Schödel J., Sims D., Tumber A., Lippl K., Mole D.R., Pugh C.W., Ratcliffe P.J., Ponting C.P. (2016). Tuning the transcriptional response to hypoxia by inhibiting hypoxia-inducible factor (HIF) prolyl and asparaginyl hydroxylases. J. Biol. Chem..

[35] Tian Y.-M., Yeoh K.K., Lee M.K., Eriksson T., Kessler B.M., Kramer H.B., Edelmann M.J., Willam C., Pugh C.W., Schofield C.J. (2011). Differential sensitivity of hypoxia inducible factor hydroxylation sites to hypoxia and hydroxylase inhibitors. J. Biol. Chem..

[36] Yeh T.-L., Leissing T., Abboud M.I., Thinnes C.C., Atasoylu O., Holt-Martyn J.P., Zhang D., Tumber A., Lippl K., Lohans C.T. (2017). Molecular and cellular mechanisms of HIF prolyl hydroxylase inhibitors in clinical trials. Chem. Sci..

[37] Dann C.E., Bruick R.K., Deisenhofer J. (2002). Structure of factor-inhibiting hypoxia-inducible factor 1: An asparaginyl hydroxylase involved in the hypoxic response pathway. Proc. Natl. Acad. Sci. USA.

[38] Nagel S., Talbot N.P., Mecinovic J., Smith T.G., Buchan A.M., Schofield C.J. (2010). Therapeutic manipulation of the HIF hydroxylases. Antioxid. Redox Signal..

[39] Rose N.R., McDonough M.A., King O.N., Kawamura A., Schofield C.J. (2011). Inhibition of 2-oxoglutarate dependent oxygenases. Chem. Soc. Rev..

[40] Tsujita T., Kawaguchi S.-I., Dan T., Baird L., Miyata T., Yamamoto M. (2015). Hypoxia-sensitive reporter system for high-throughput screening. Tohoku J. Exp. Med..

[41] Dayan F., Monticelli M., Pouyssegur J., Pecou E. (2009). Gene regulation in response to graded hypoxia: The non-redundant roles of the oxygen sensors PHD and FIH in the HIF pathway. J. Theor. Biol..

[42] Guenzler-Pukall V.W.Q., Langsetmo P.I., Guo G. (2009). Methods for Reducing Blood Pressure. WO Patent.

[43] Thomsen R., Christensen M.H. (2006). Moldock: A new technique for high-accuracy molecular docking. J. Med. Chem..

[44] Jiang B.-H., Zheng J.Z., Roe L.R., Semenza G.L. (1997). Transactivation and inhibitory domains of hypoxia-inducible factor-1α. J. Biol. Chem..

[45] Rose N.R., Ng S.S., Mecinovic J., Lienard B.M.R., Bello S.H., Sun Z., McDonough M.A., Oppermann U., Schofield C.J. (2008). Inhibitor scaffolds for 2-oxoglutarate-dependent histone lysine demethylases. J. Med. Chem..

[46] Amatore C., Jutand A., Negri S., Fauvarque J.F. (1990). Efficient palladium-catalyzed synthesis of unsymmetrical donor-acceptor biaryls and polyaryls. J. Organomet. Chem..

[47] Tserng K.Y., Bauer L. (1975). Synthesis of 3-hydroxythienopyrimidine-2,4(1H,3H)-diones from 2,3-thiophenedicarboxylic and 3,4-thiophenedicarboxylic acids. J. Org. Chem..

[48] Li J.J., Carson K.G., Trivedi B.K., Yue W.S., Ye Q., Glynn R.A., Miller S.R., Connor D.T., Roth B.D., Luly J.R. (2003). Synthesis and structure–activity relationship of 2-amino-3-heteroaryl-quinoxalines as non-peptide, small-molecule antagonists for interleukin-8 receptor. Bioorg. Med. Chem..

[49] Murasawa S., Iuchi K., Sato S., Noguchi-Yachide T., Sodeoka M., Yokomatsu T., Dodo K., Hashimoto Y., Aoyama H. (2012). Small-molecular inhibitors of Ca^2+^-induced mitochondrial permeability transition (MPT) derived from muscle relaxant dantrolene. Bioorg. Med. Chem..

[50] Itahara T. (1985). Arylation of aromatic heterocycles with arenes and palladium(II) acetate. J. Org. Chem..

[51] Frizler M., Schmitz J., Schulz-Fincke A.-C., Gütschow M. (2012). Selective nitrile inhibitors to modulate the proteolytic synergism of cathepsins S and F. J. Med. Chem..

[52] Horio Y., Ootake Y., Sawaki S., Inukai S., Agata M., Umezawa M., Goto M. (1993). Tetrazoleacetic Acid Derivatives Having Aldose Reductase Inhibitory Activity. U.S. Patent.

[53] Antonow D., Marrafa T., Dawood I., Ahmed T., Haque M.R., Thurston D.E., Zinzalla G. (2010). Facile oxidation of electron-poor benzo[b]thiophenes to the corresponding sulfones with an aqueous solution of H_2_O_2_ and P_2_O_5_. Chem. Commun..

