# Supplementary material for: Furan- and Thiophene-2-Carbonyl Amino Acid Derivatives Activate Hypoxia-Inducible Factor via Inhibition of Factor Inhibiting Hypoxia-Inducible Factor-1
Source: Molecules. 2018 Apr 11;23(4):885. doi: 10.3390/molecules23040885 (PMC6017830; doi:10.3390/molecules23040885)

Supporting Information for

Furan- and thiophene-2-carbonyl amino acid derivatives activate the hypoxia inducible factor via inhibition of factor inhibiting HIF-1

Shin-ichi Kawaguchi [1,2,3], Yuhei Gonda [2], Takuya Yamamoto [2], Yuki Sato [2], Hiroyuki Shinohara [2], Yohsuke Kobiki [2], Atsuhiko Ichimura [3,4],Takashi Dan [3], Motohiro Sonoda [2,5], Toshio Miyata [3] Akiya Ogawa [2], and Tadayuki Tsujita [3,6,*]

[1] Center for Education and Research in Agricultural Innovation, Faculty of Agriculture, Saga University, 152-1 Shonan-cho, Karatsu, Saga 847-0021, Japan; skawa@cc.saga-u.ac.jp
[2] Department of Applied Chemistry, Graduate School of Engineering, Osaka Prefecture University, 1-1 Gakuen-cho, Naka-ku, Sakai, Osaka 599-8531, Japan
[3] Department of Molecular Medicine and Therapy, Tohoku University Graduate School of Medicine, 2-1 Seiryo-machi, Aobaku, Sendai, Miyagi 980-8575, Japan
[4] Keihanshin Consortium for Fostering the Next Generation of Global Leaders in Research (K-CONNEX), Kyoto University, Kyoto 606-8501, Japan
[5] Department of Applied Biosciences, Graduate School of Life and Environmental Sciences, Osaka Prefecture University, 1-1 Gakuen-cho, Naka-ku, Sakai, Osaka 599-8531, Japan
[6] Department of Applied Biochemistry and Food Science, Faculty of Agriculture, Saga University, 1 Honjo-machi, Saga, 840-8502, Japan; tada@cc.saga-u.ac.jp

* Correspondence: tada@cc.saga-u.ac.jp; Tel.: +81-952-28-8771

CONTENTS

page

HPLC chart of compound 16 S3

Copies of ^{1}H NMR spectra of the compounds (1-44) S4-S25

HPLC chart for compound 16 (solvent : hexane/ 2-propanol = 1/ 1)

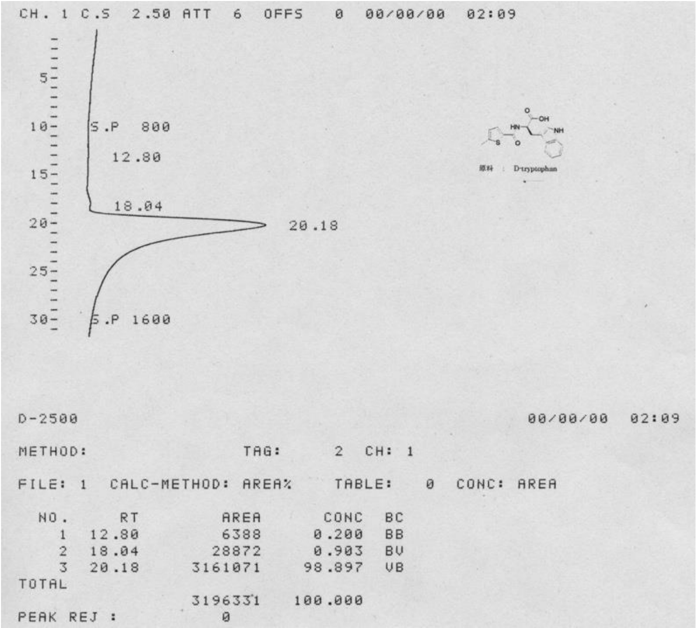

HPLC chart for the *RS*-racemic mixture of (5-methylthiophene-2-carbonyl)tryptophan

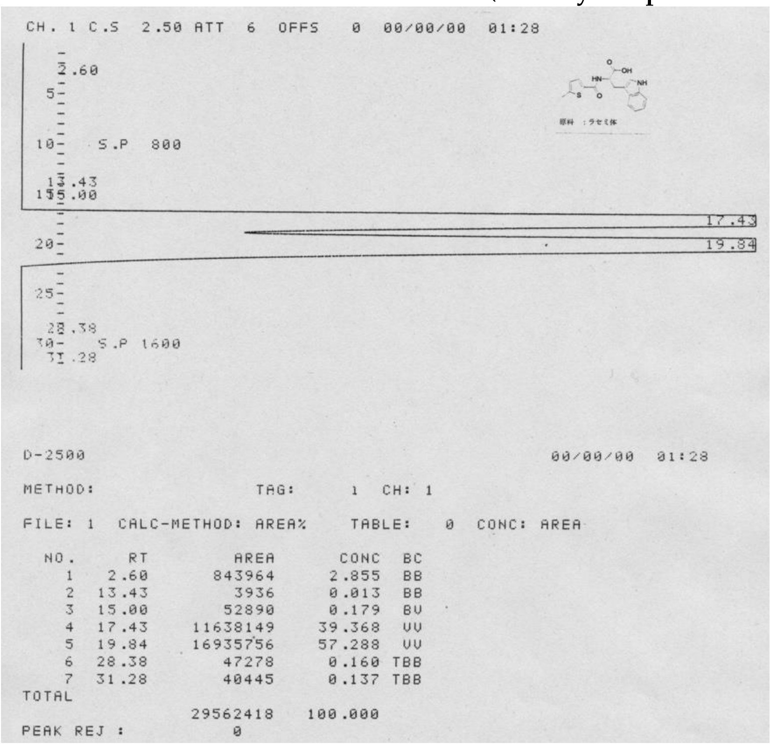

^{1}H NMR spectrum for compound 1

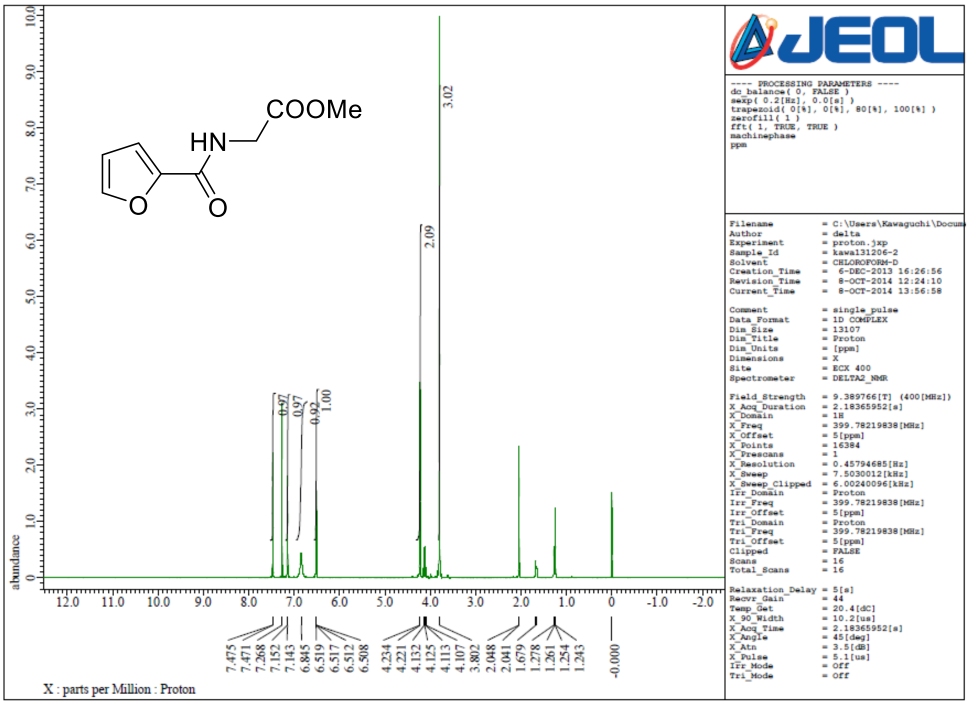

^{1}H NMR spectrum for compound 2

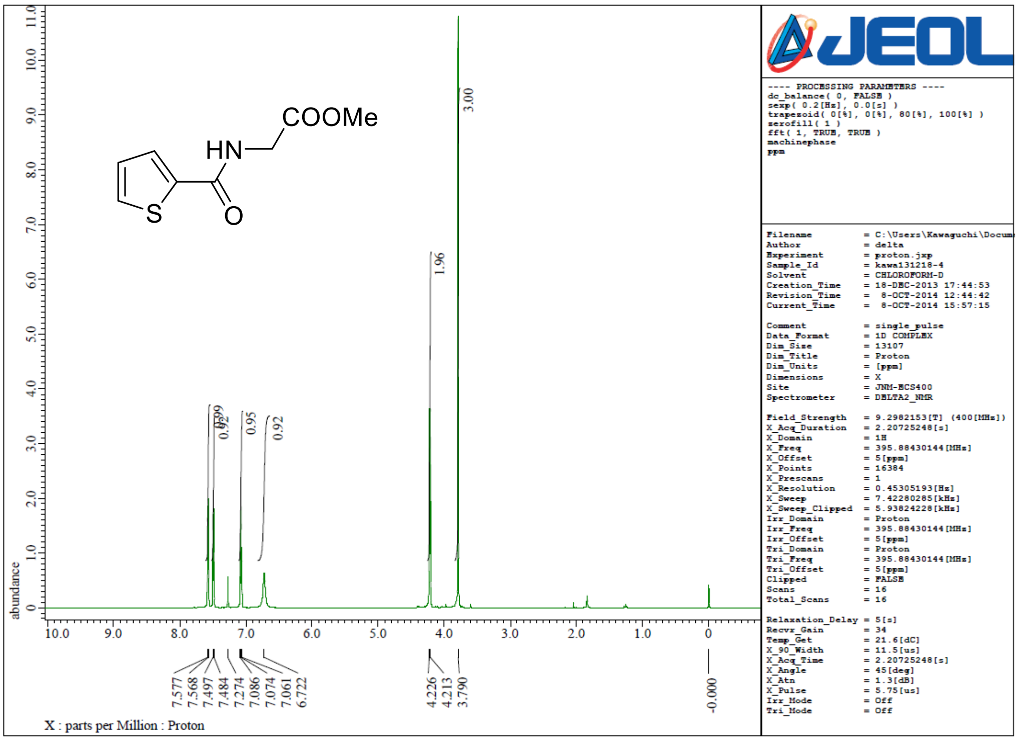

^{1}H NMR spectrum for compound 3

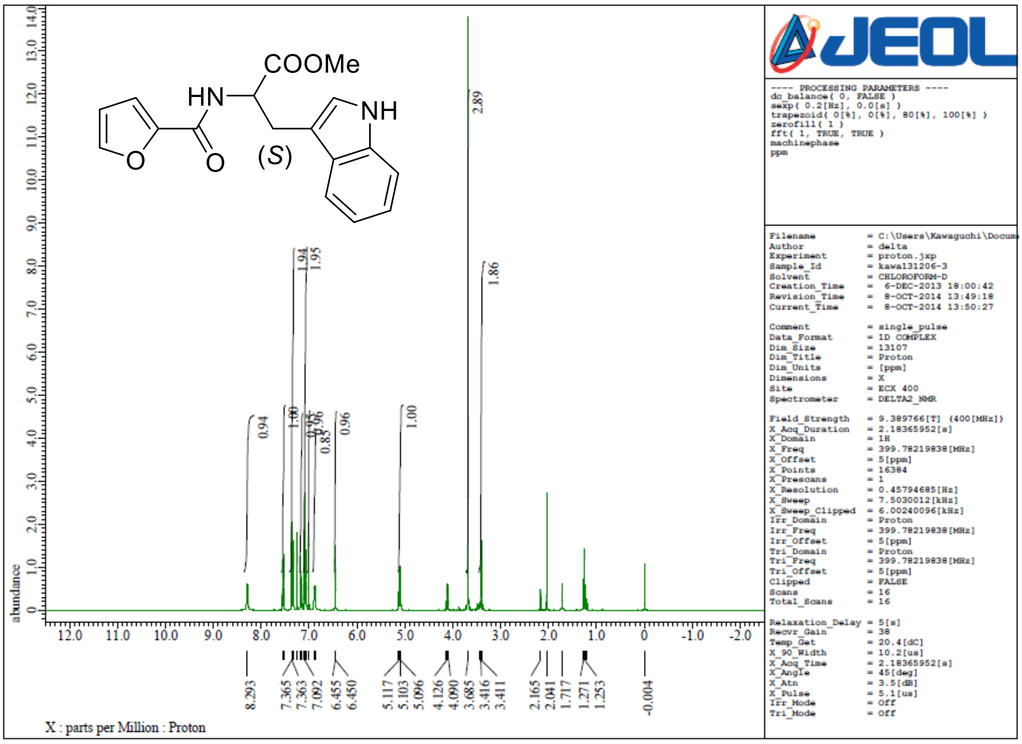

^{1}H NMR spectrum for compound 4

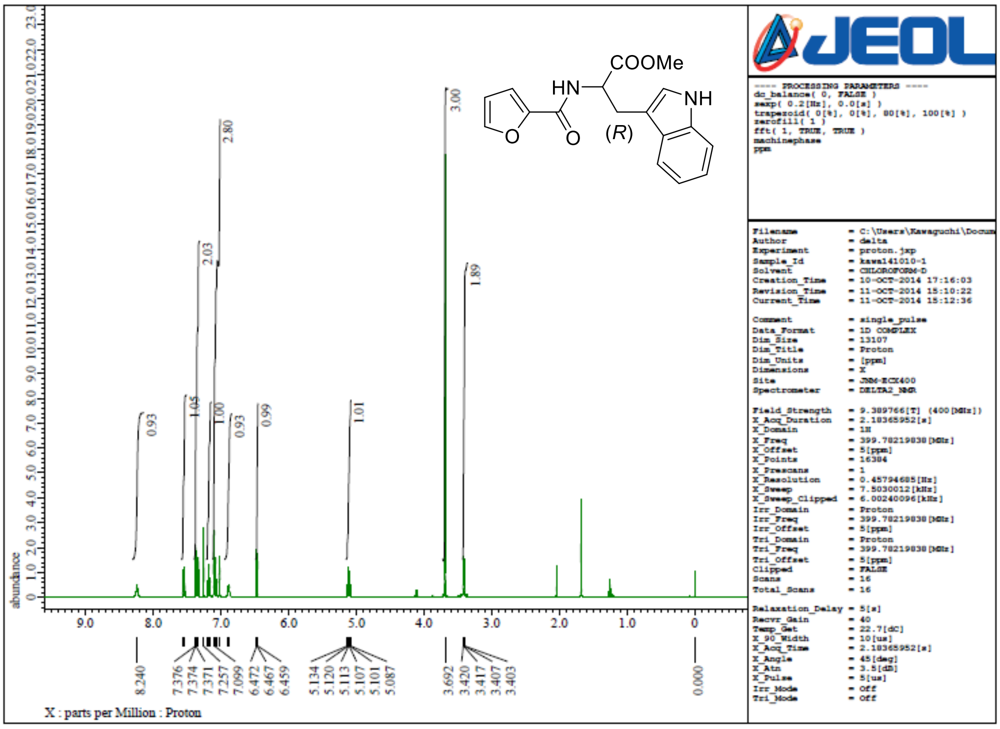

^{1}H NMR spectrum for compound 5

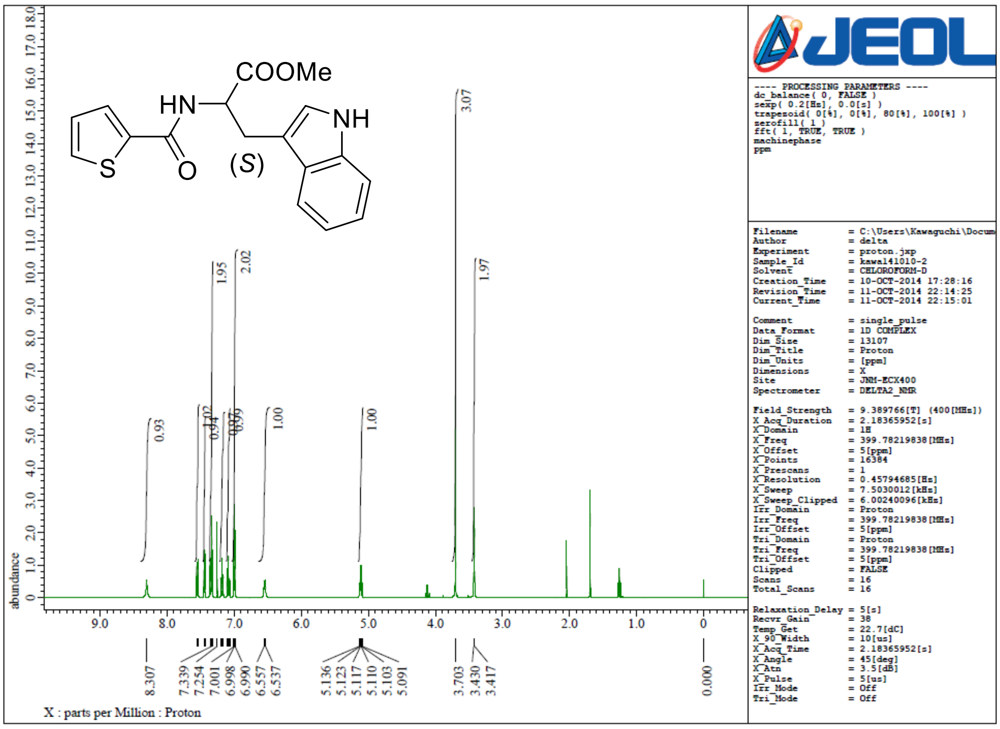

^{1}H NMR spectrum for compound 6

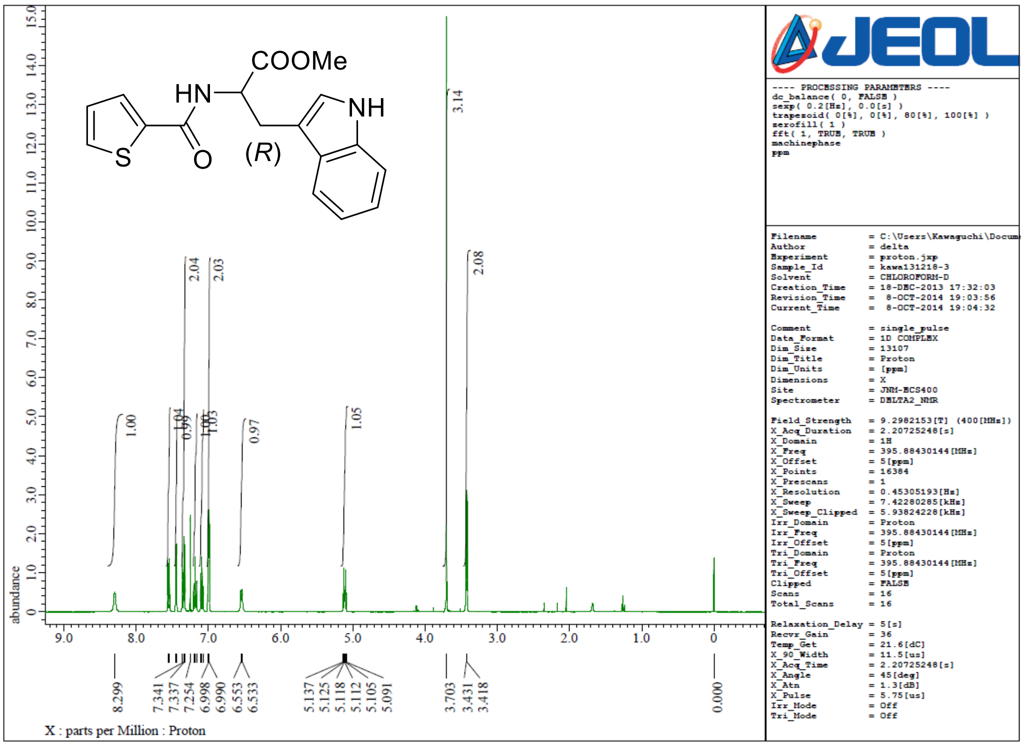

^{1}H NMR spectrum for compound 7

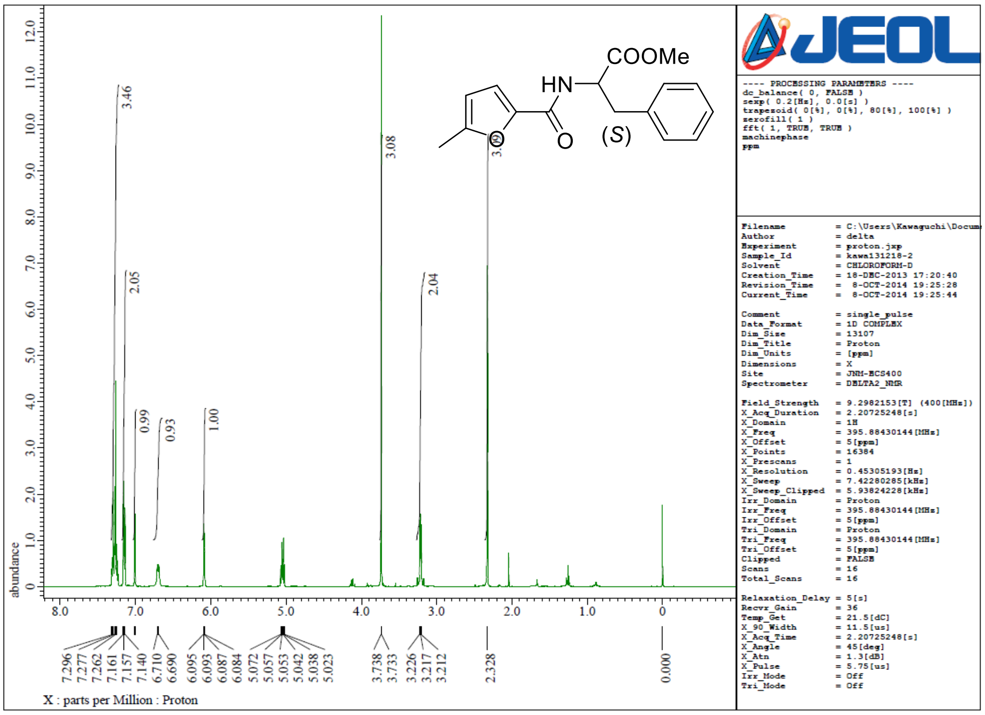

^{1}H NMR spectrum for compound 8

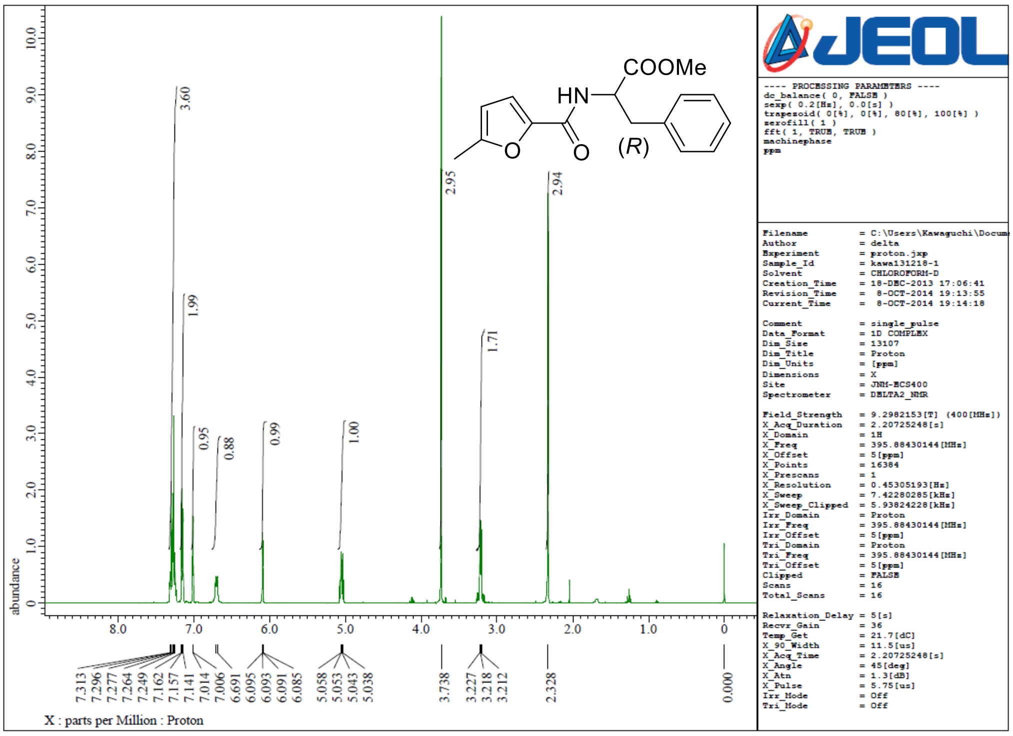

^{1}H NMR spectrum for compound 9

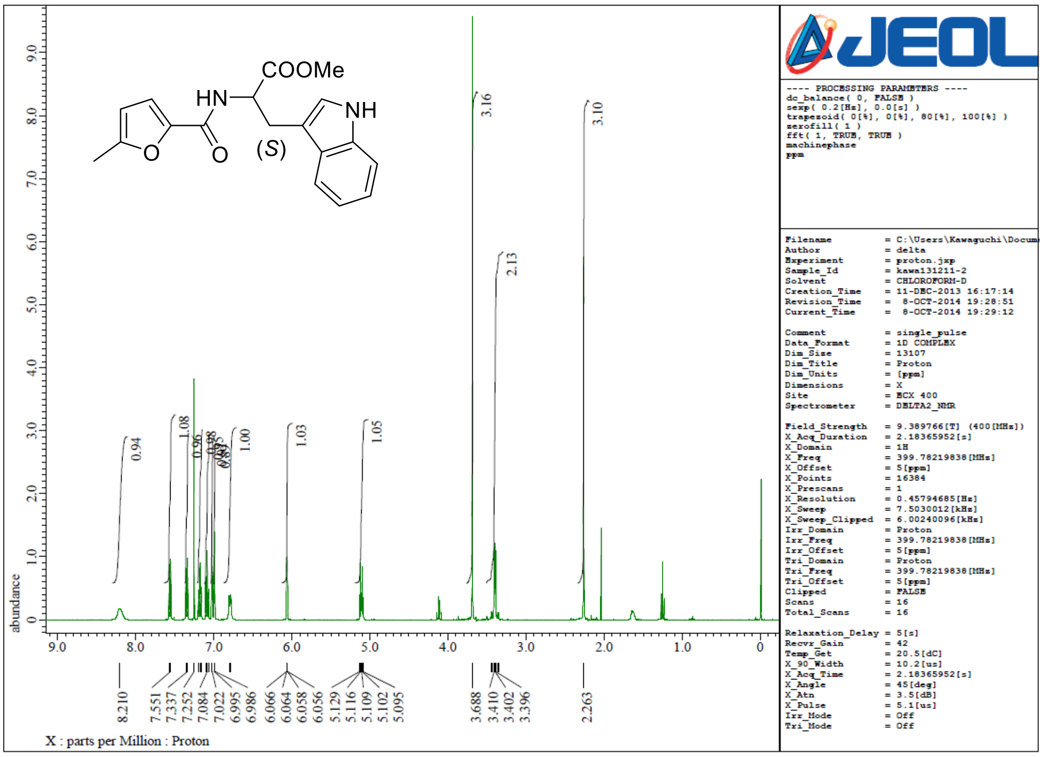

^{1}H NMR spectrum for compound 10

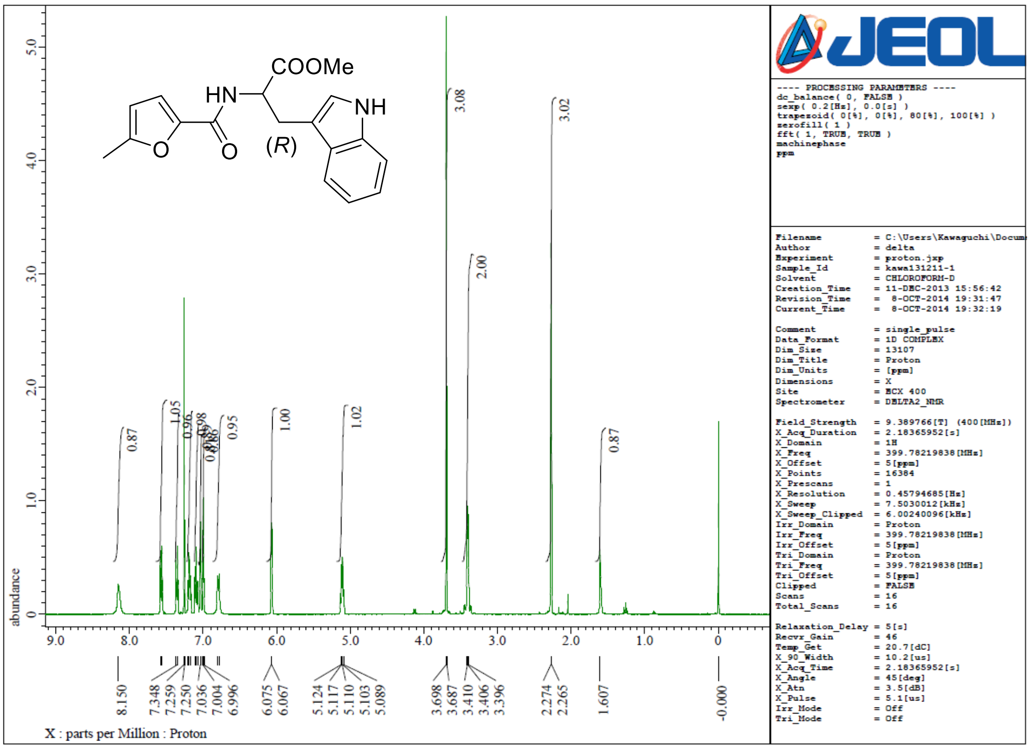

^{1}H NMR spectrum for compound 11

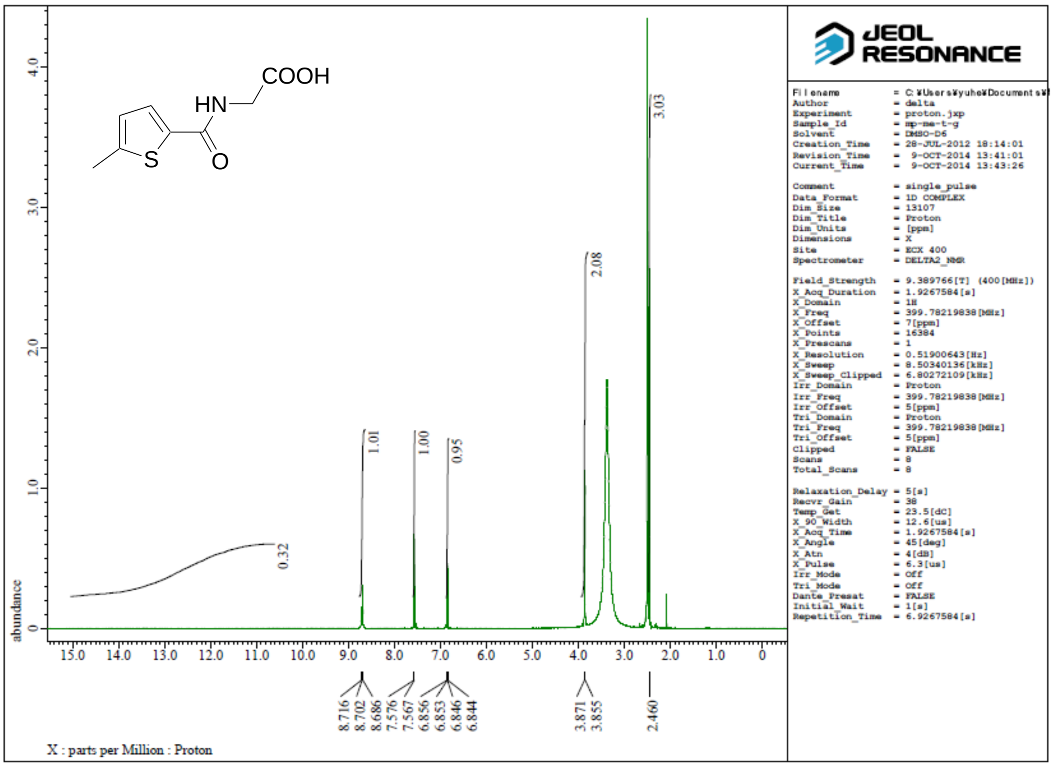

^{1}H NMR spectrum for compound 12

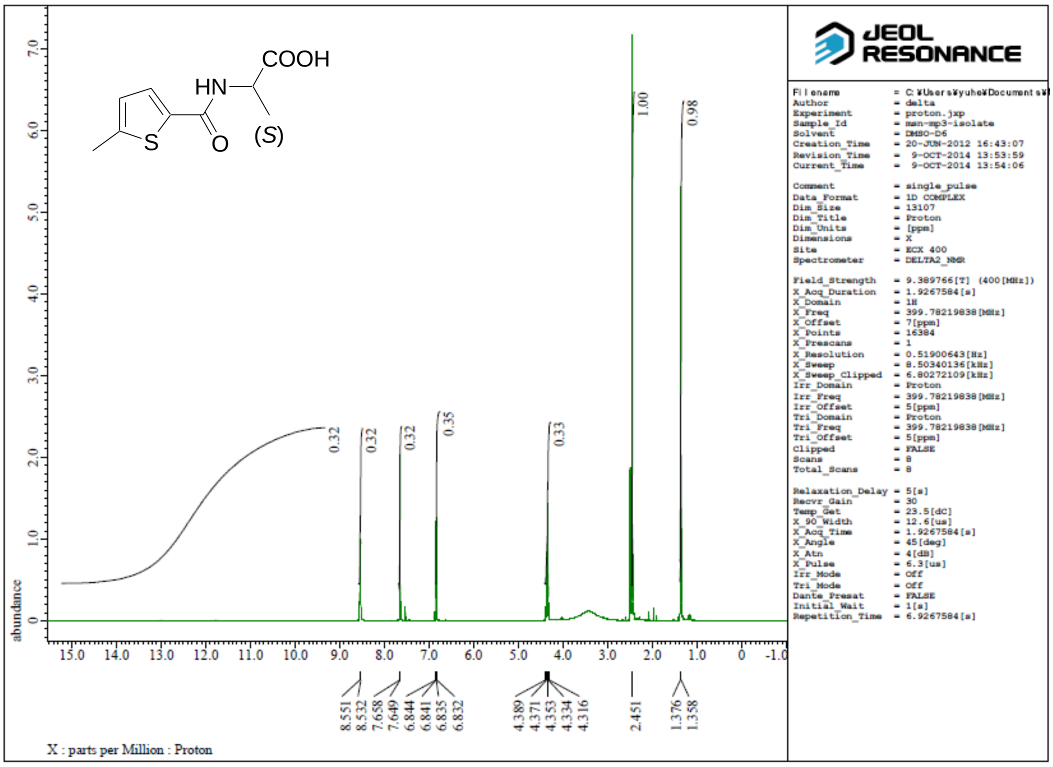

^{1}H NMR spectrum for compound 13

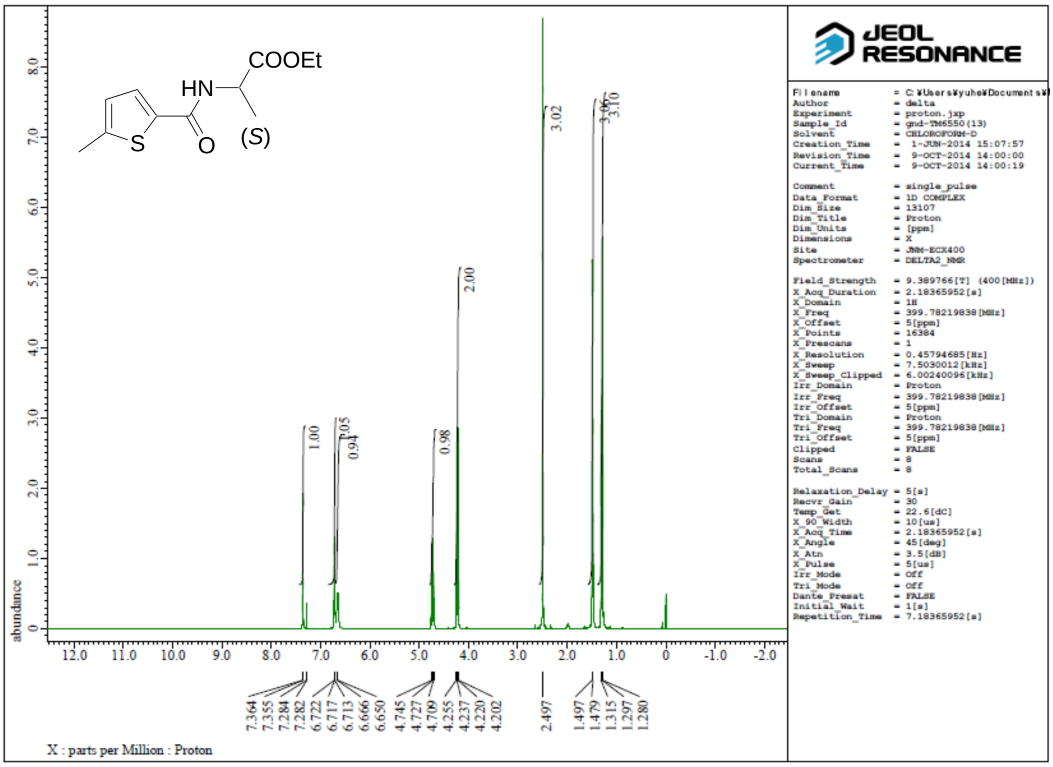

^{1}H NMR spectrum for compound 14

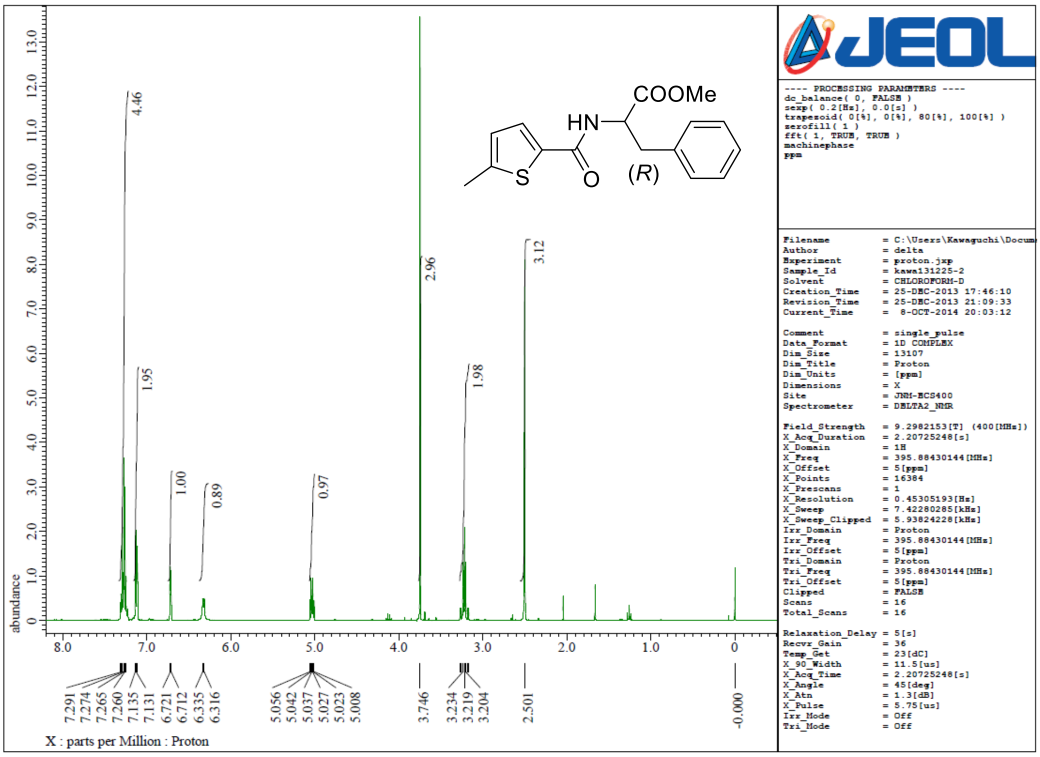

¹H NMR spectrum for compound 15

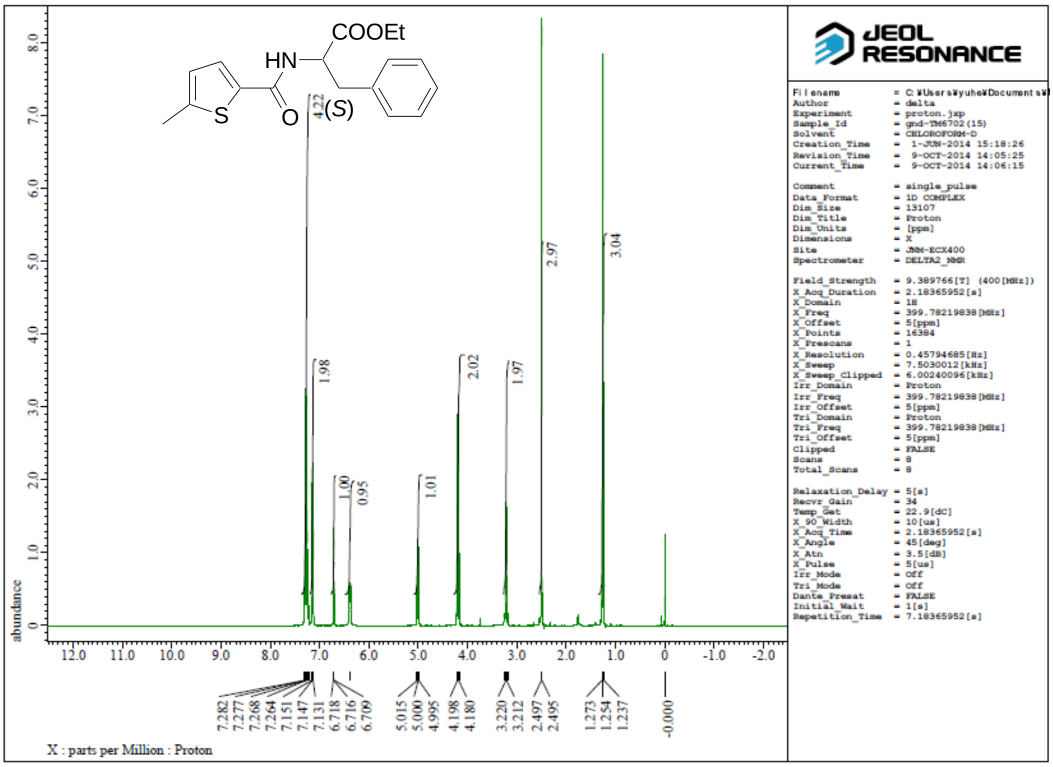

¹H NMR spectrum for compound 16

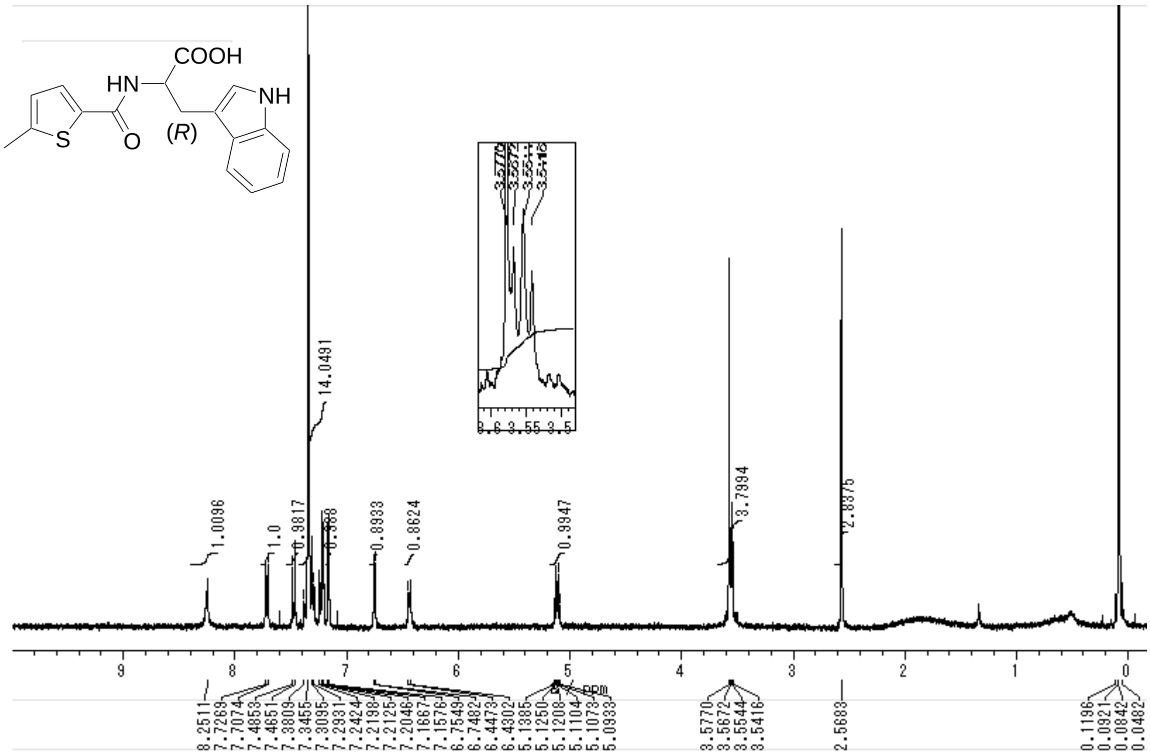

¹H NMR spectrum for compound 17

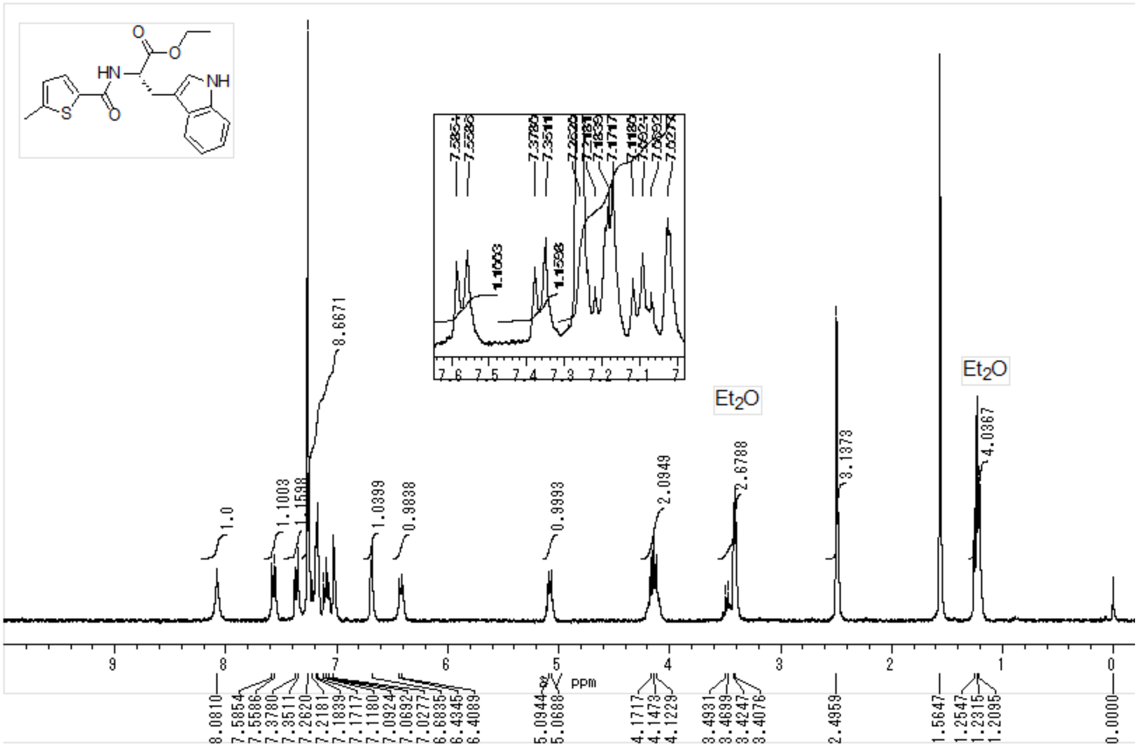

^{1}H NMR spectrum for compound 18

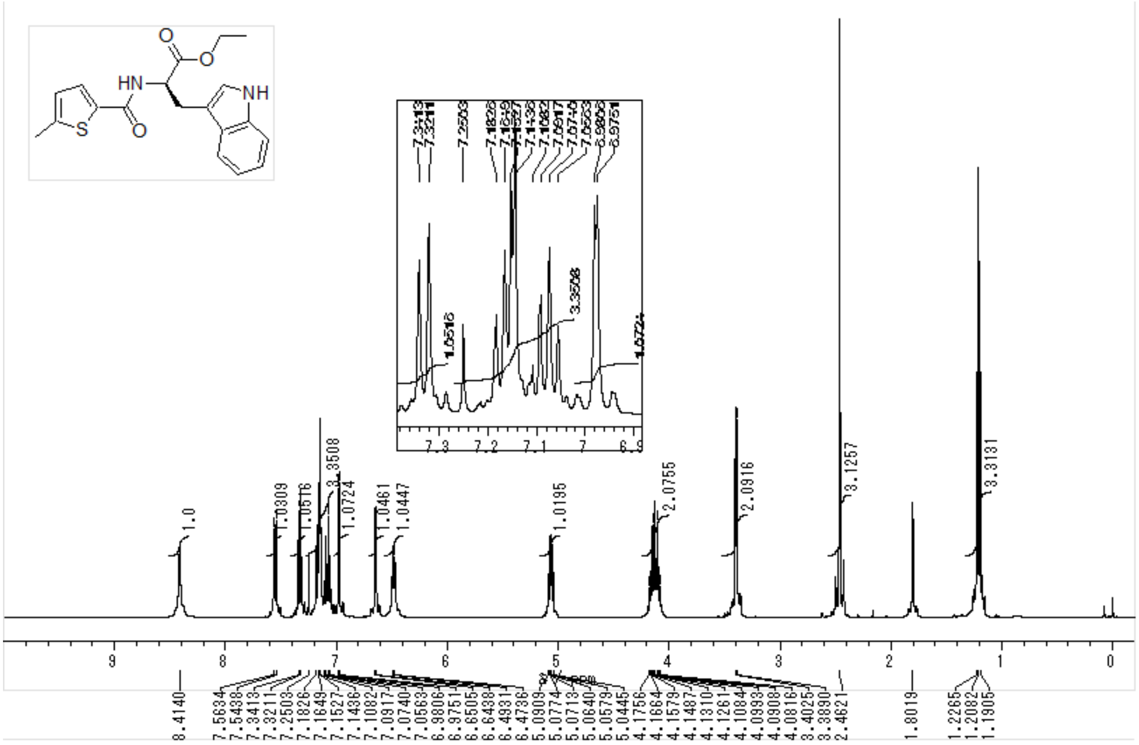

^{1}H NMR spectrum for compound 19

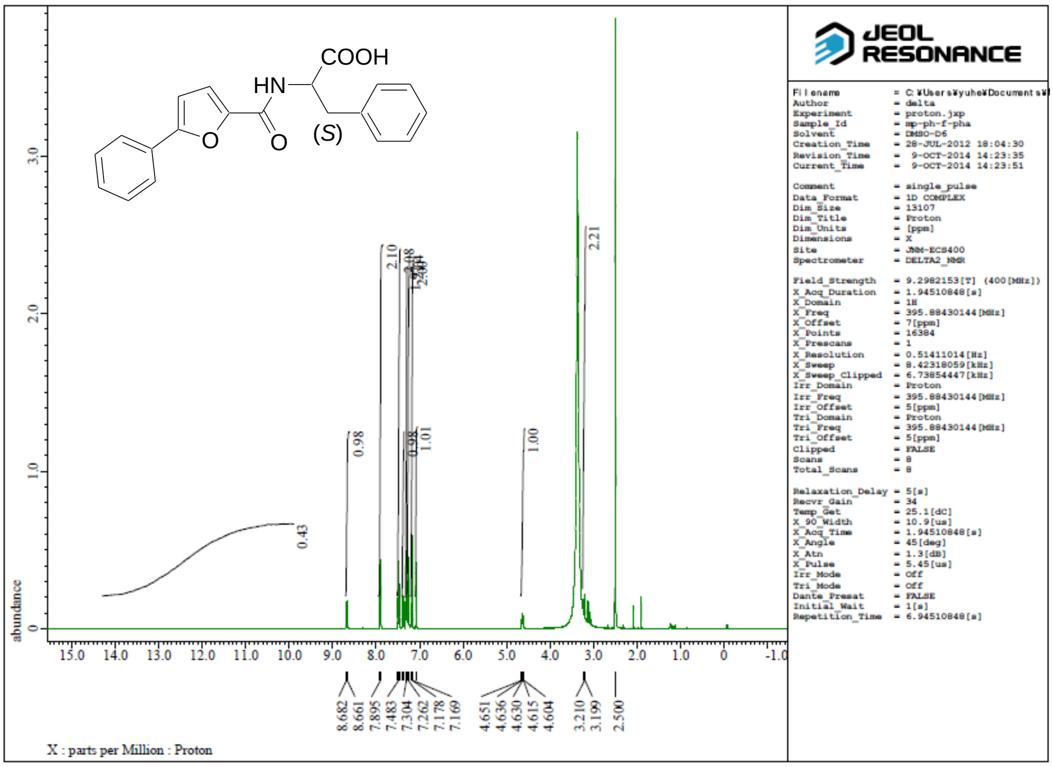

^{1}H NMR spectrum for compound 20

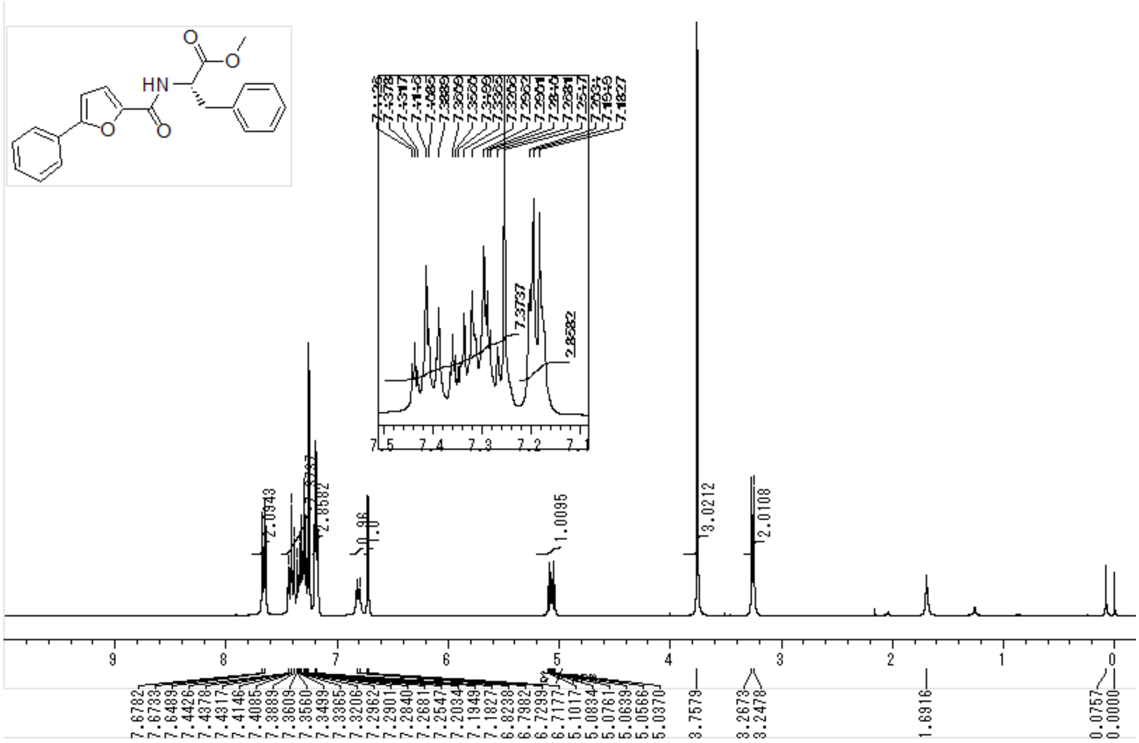

^{1}H NMR spectrum for compound 21

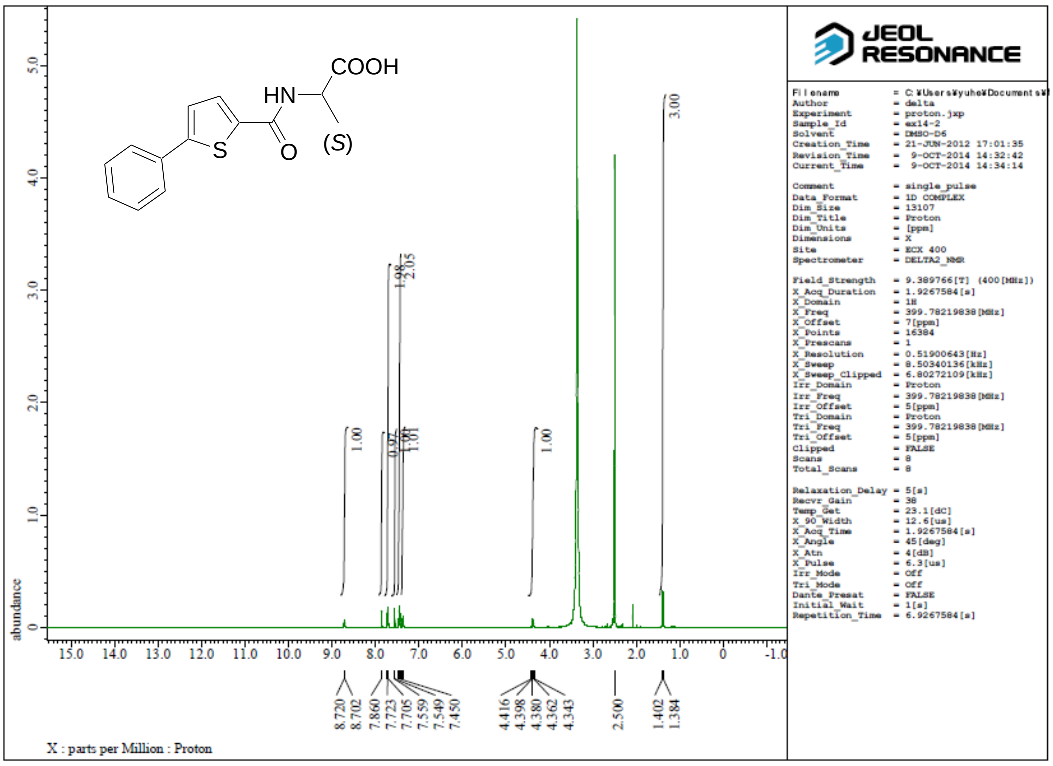

^{1}H NMR spectrum for compound 22

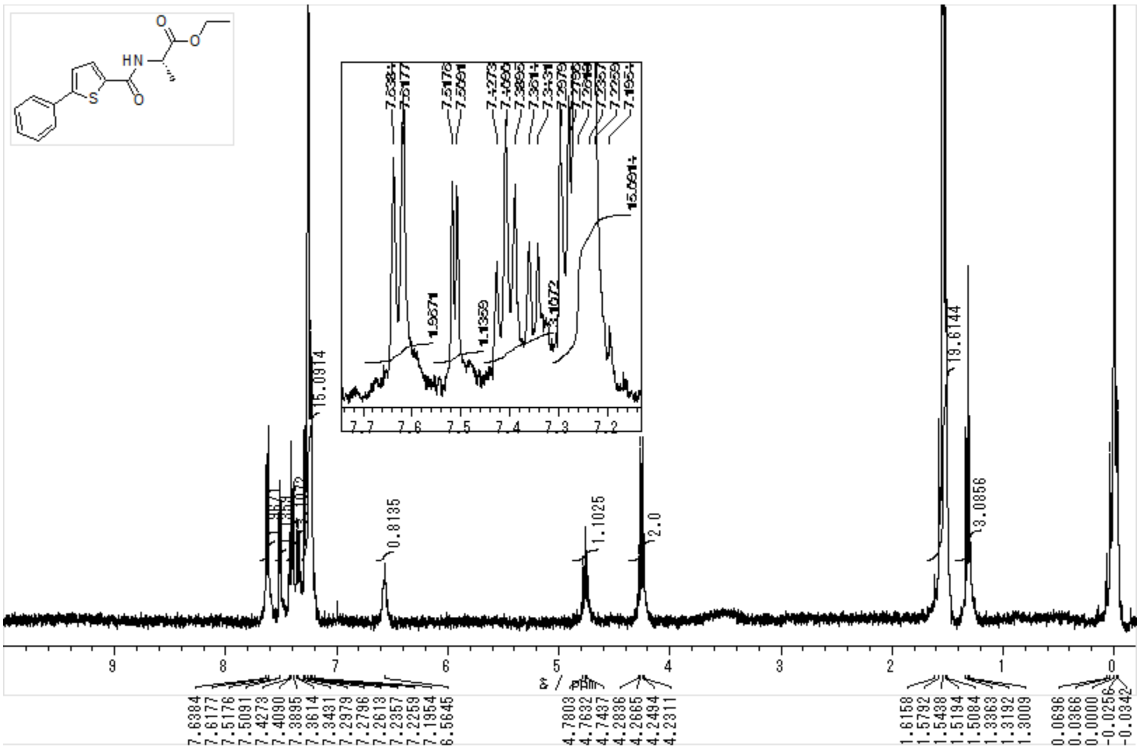

^{1}H NMR spectrum for compound 23

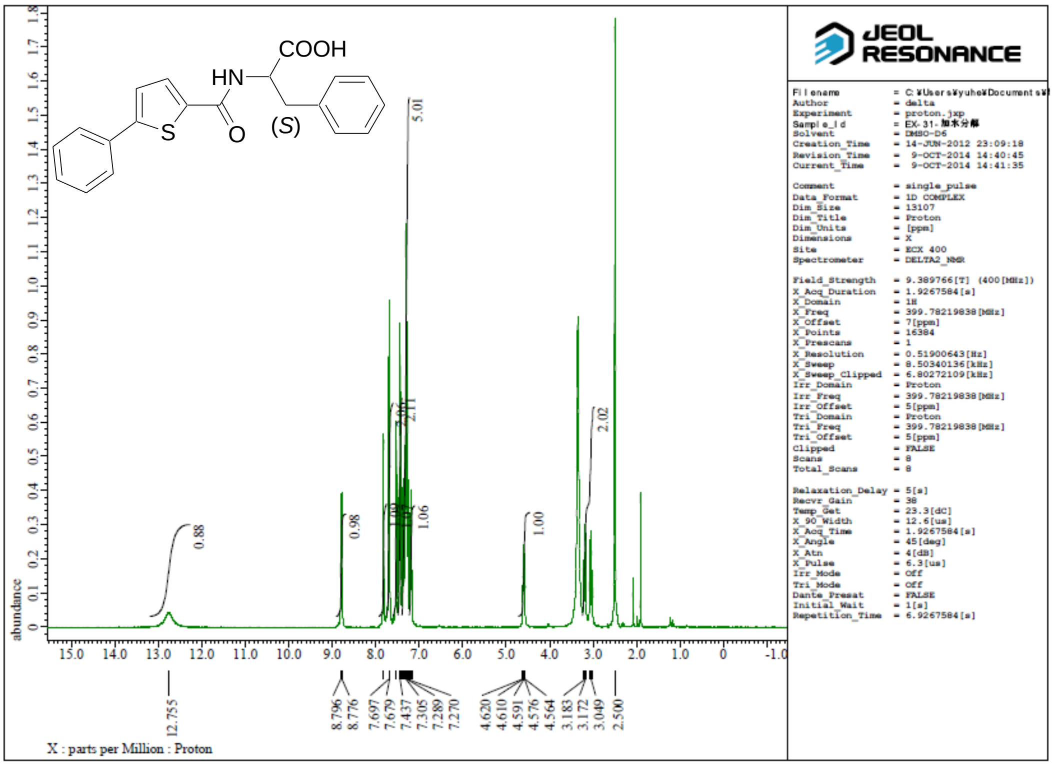

^{1}H NMR spectrum for compound 24

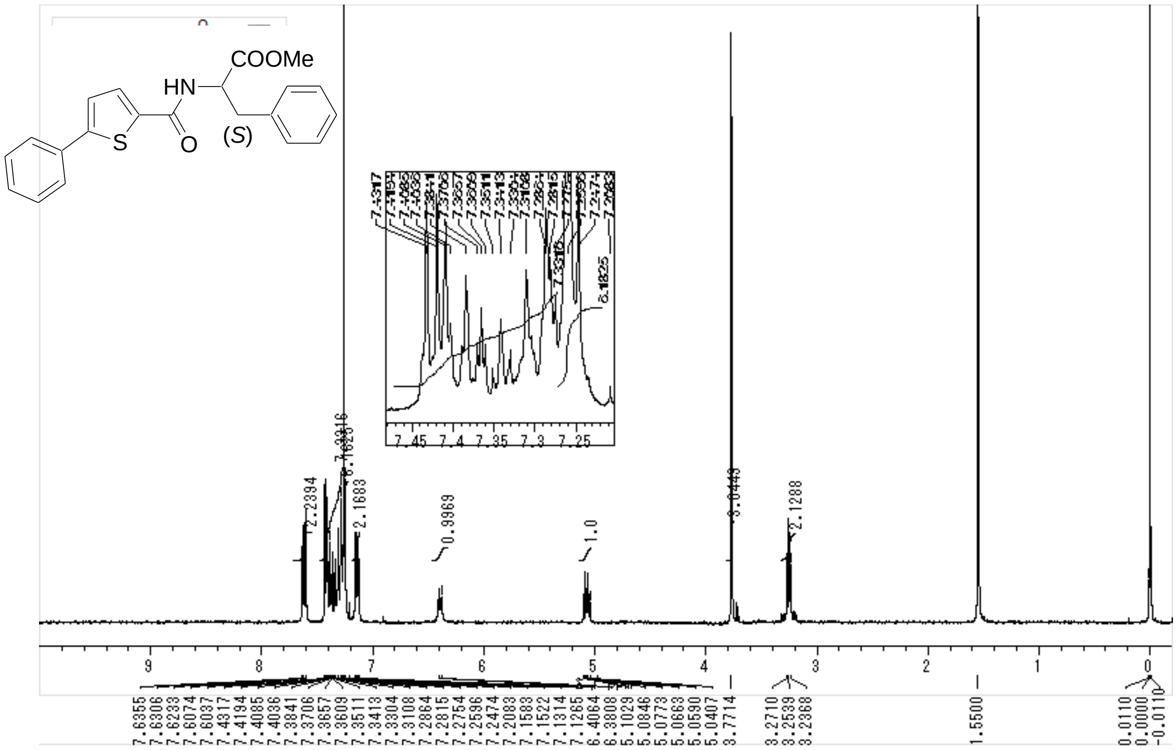

^{1}H NMR spectrum for compound 25

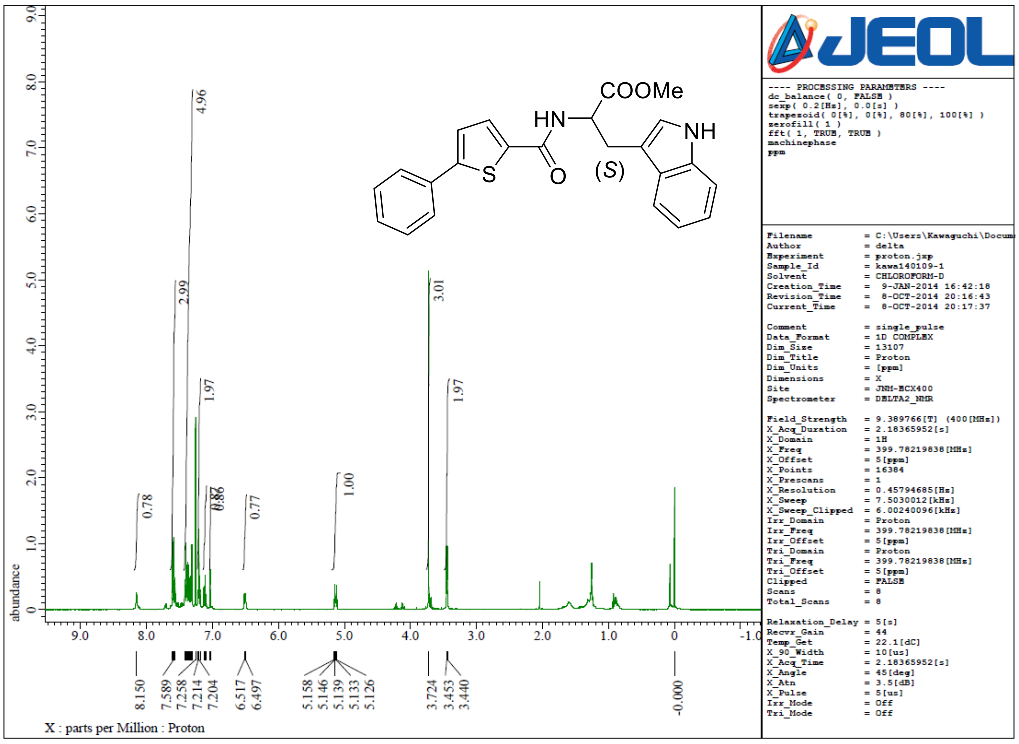

^{1}H NMR spectrum for compound 26

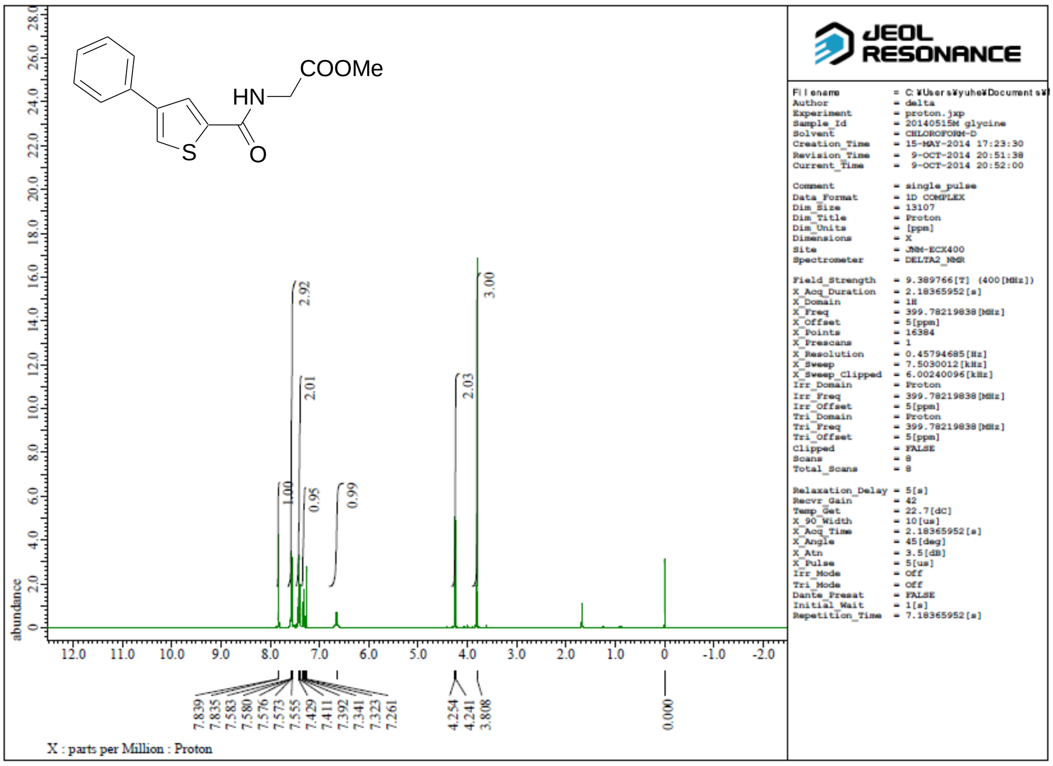

^{1}H NMR spectrum for compound 27

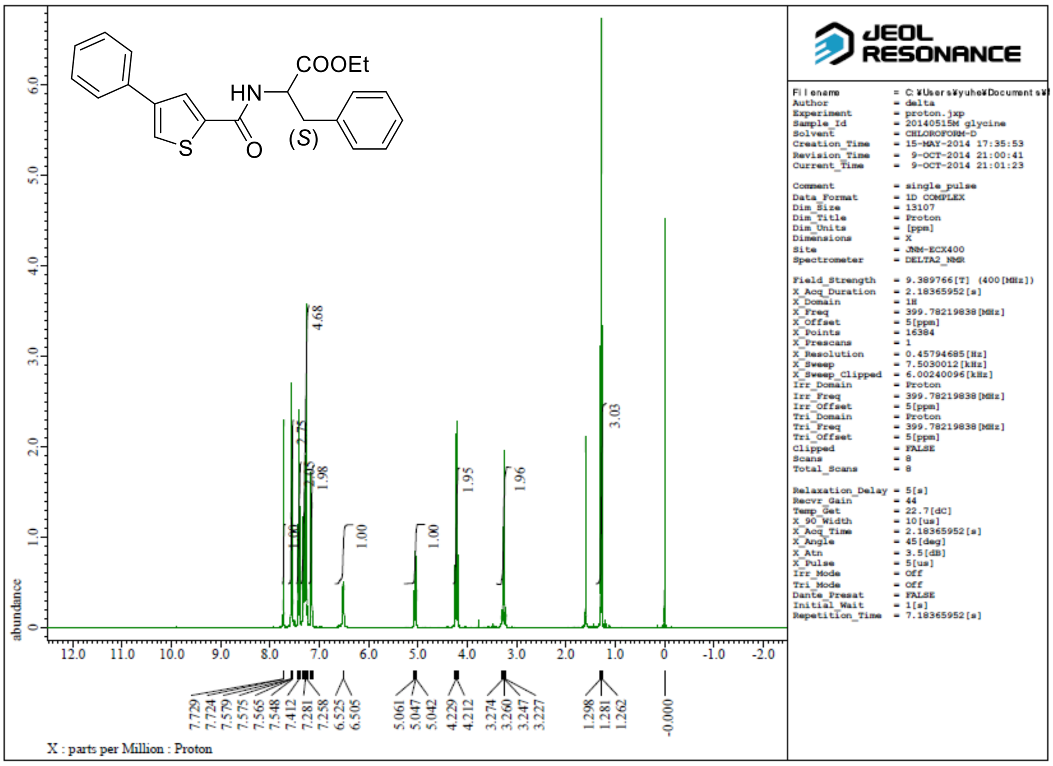

¹H NMR spectrum for compound 28

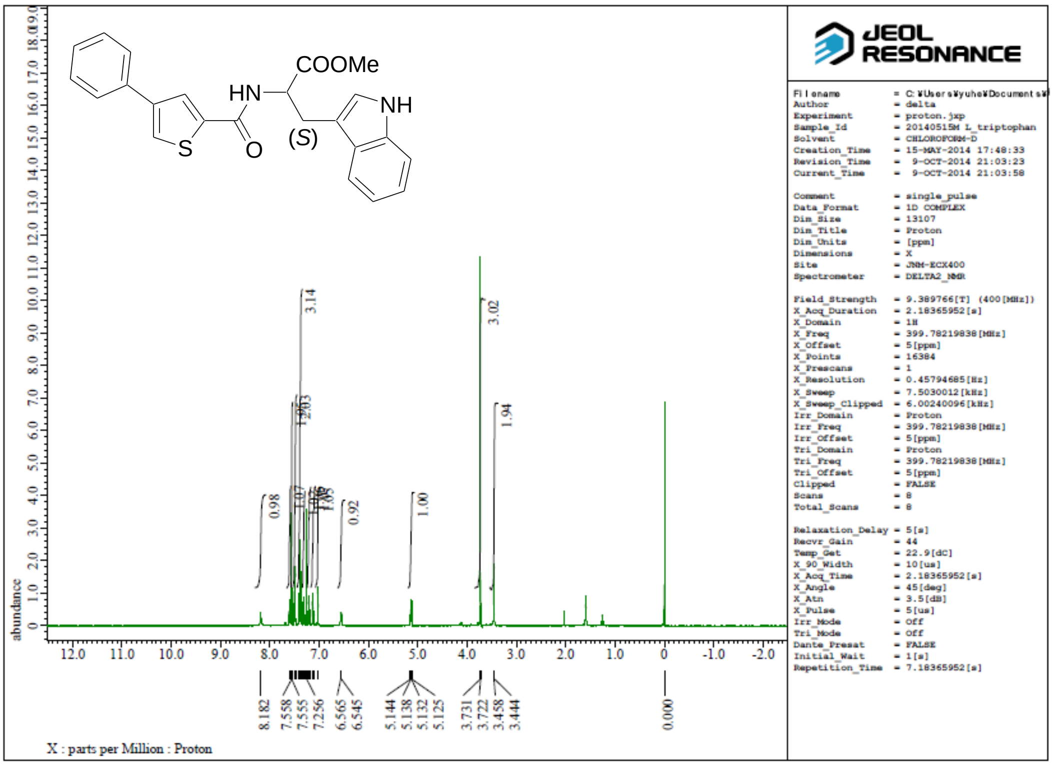

¹H NMR spectrum for compound 29

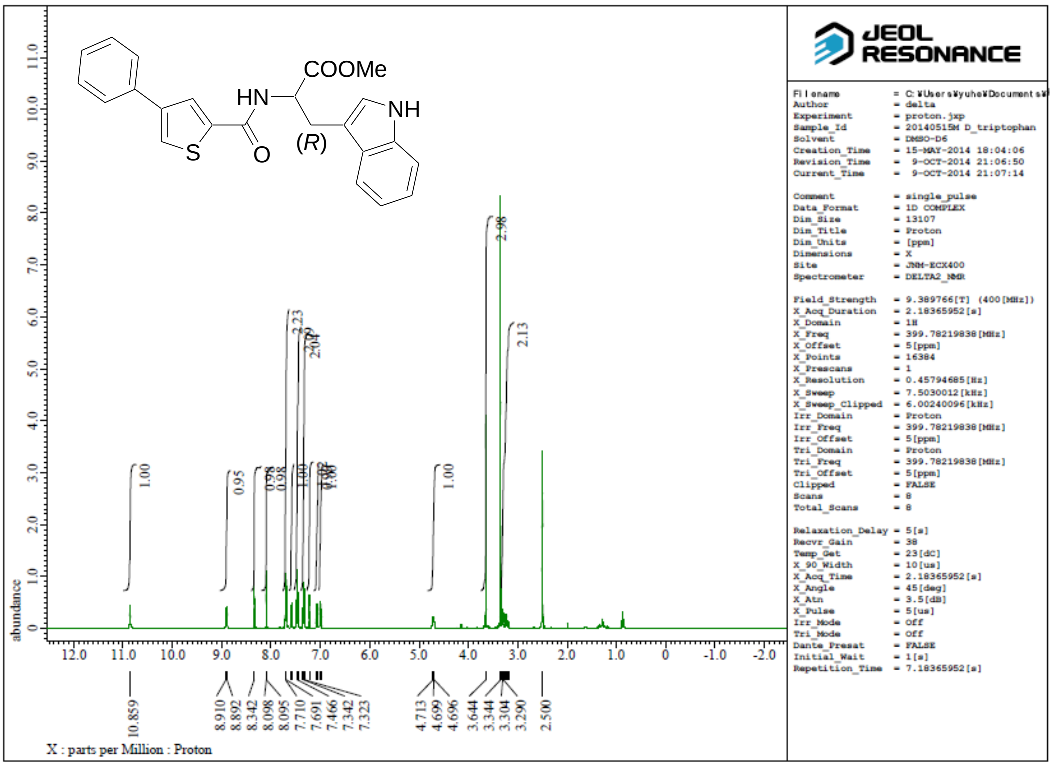

¹H NMR spectrum for compound 30

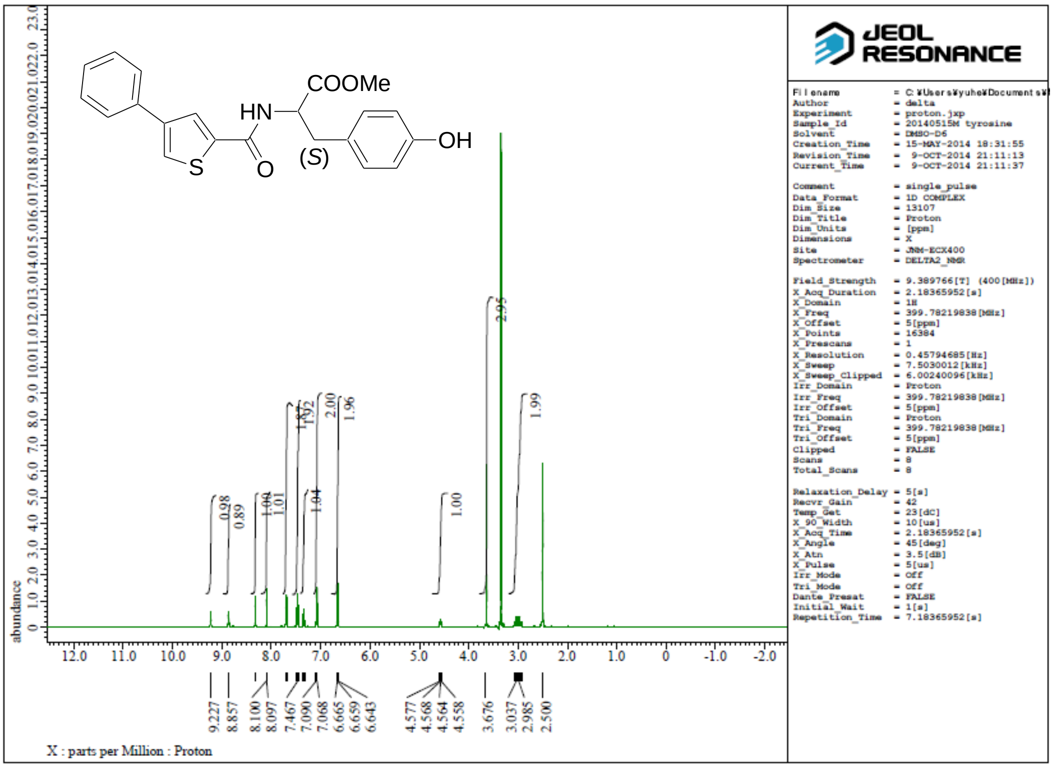

¹H NMR spectrum for compound 31

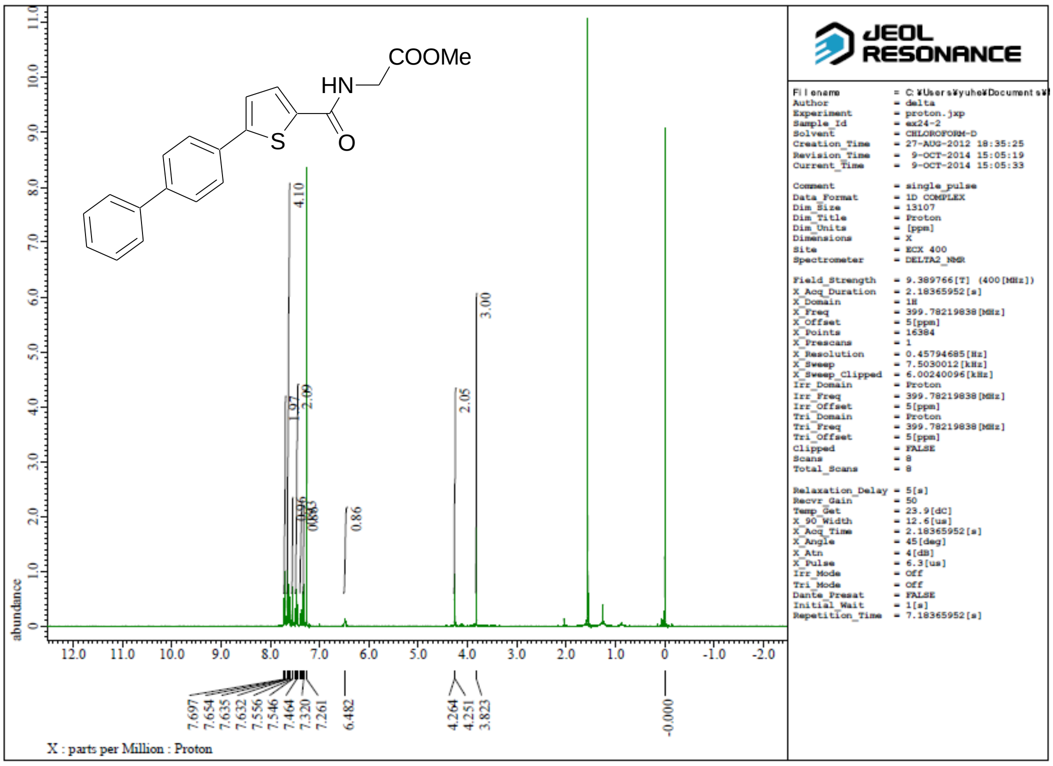

^{1}H NMR spectrum for compound 32

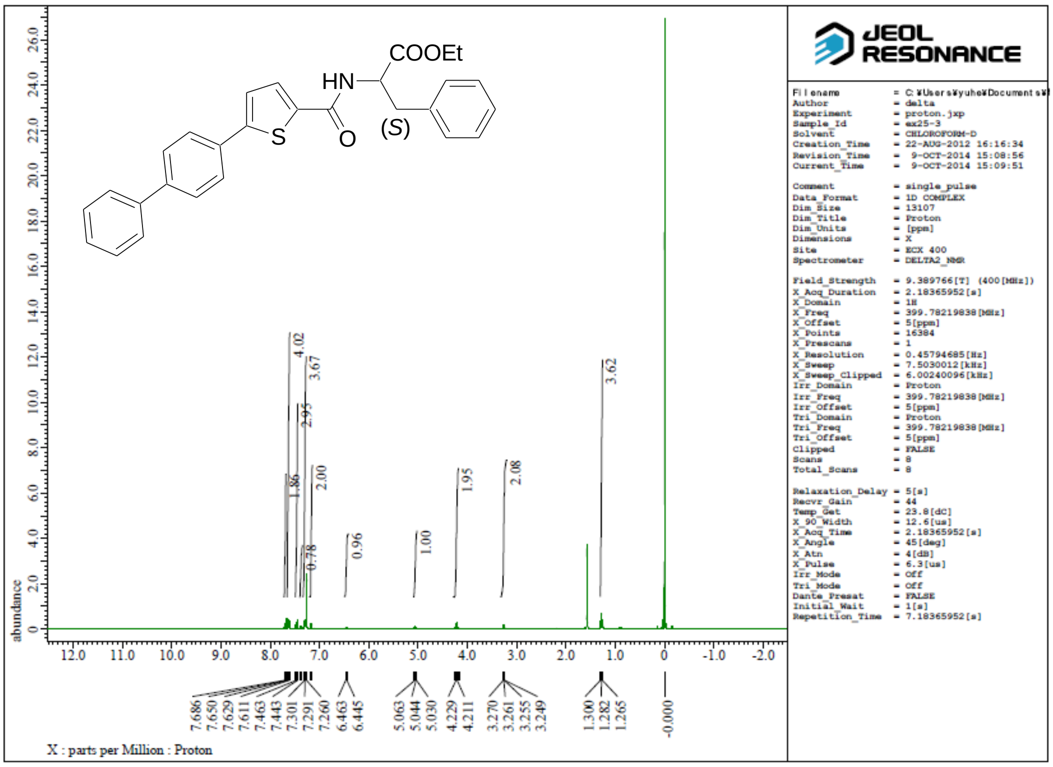

^{1}H NMR spectrum for compound 33

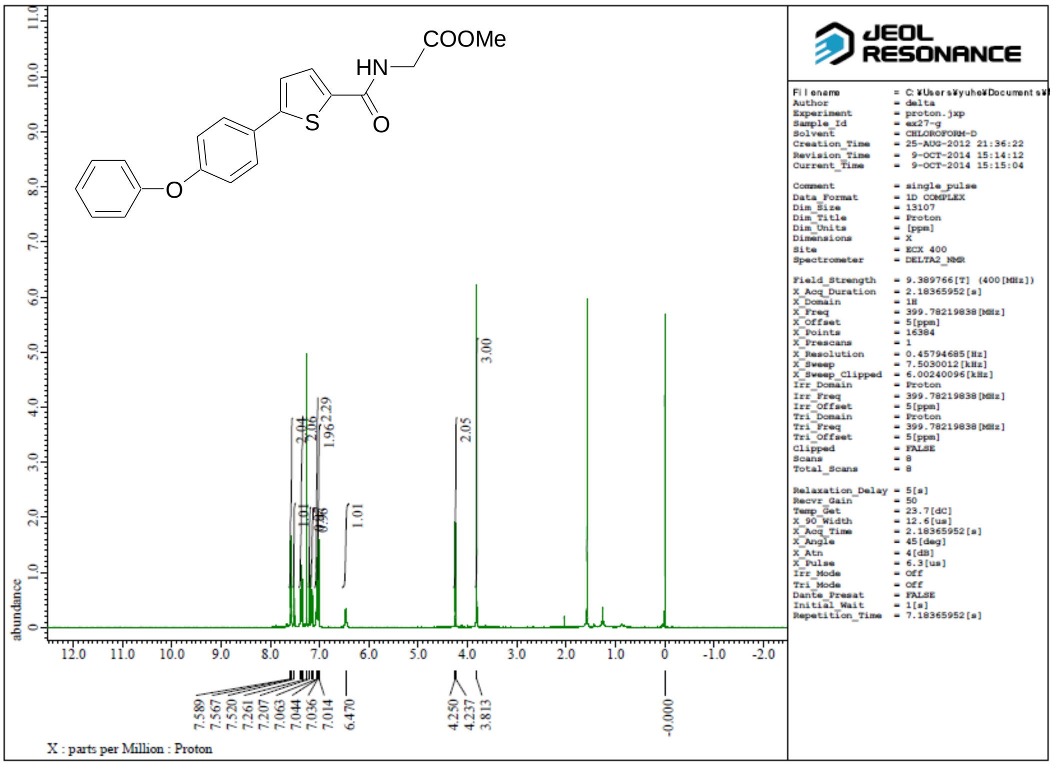

^{1}H NMR spectrum for compound 34

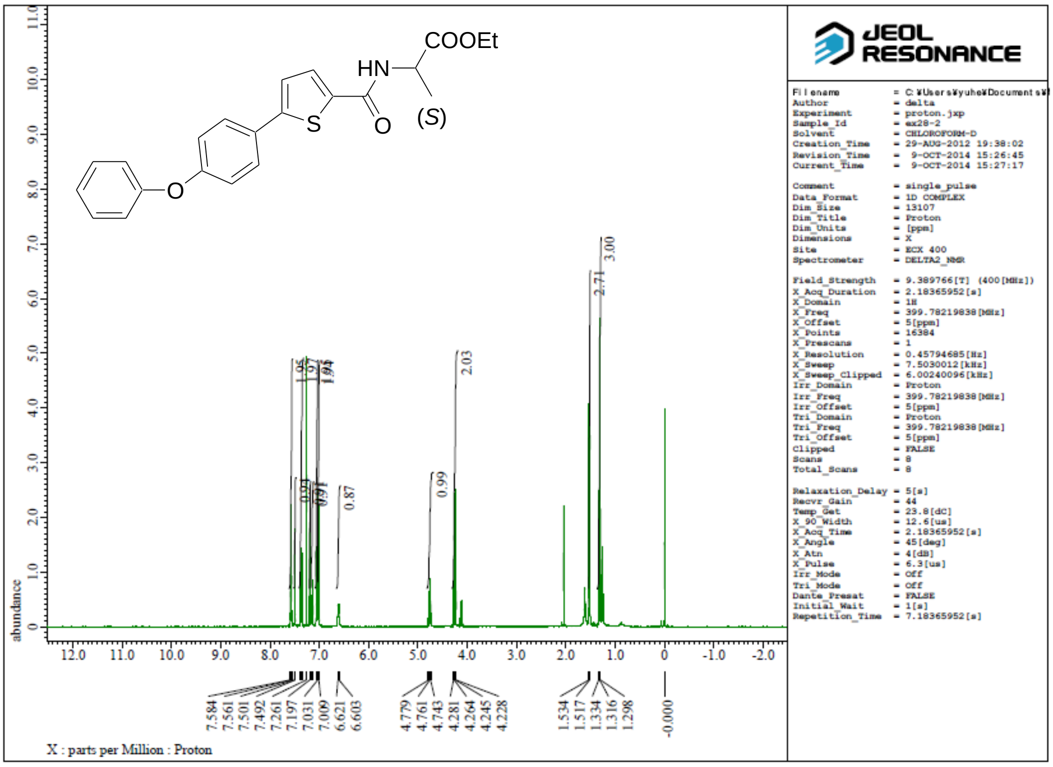

^{1}H NMR spectrum for compound 35

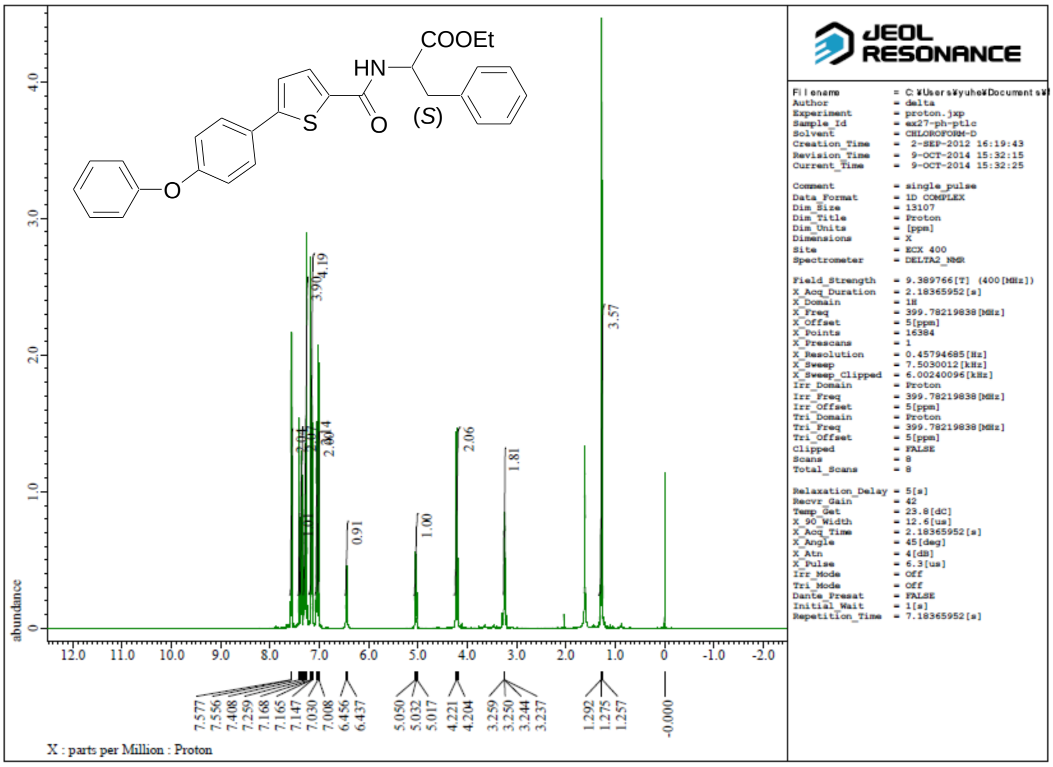

¹H NMR spectrum for compound 36

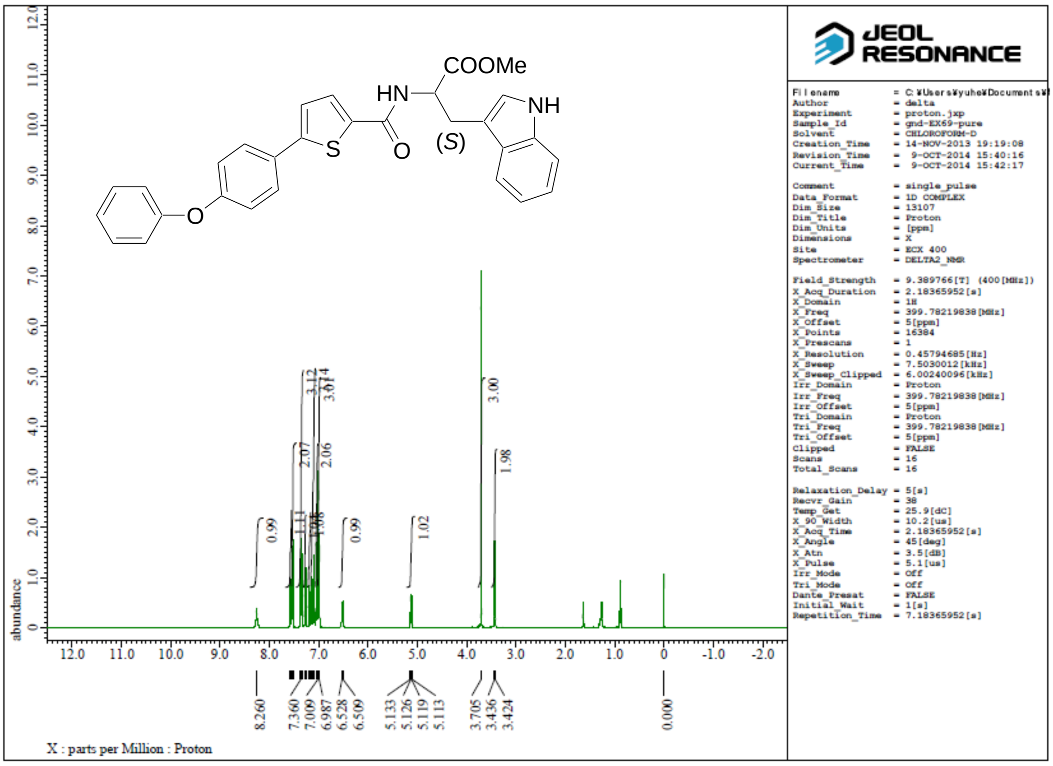

¹H NMR spectrum for compound 37

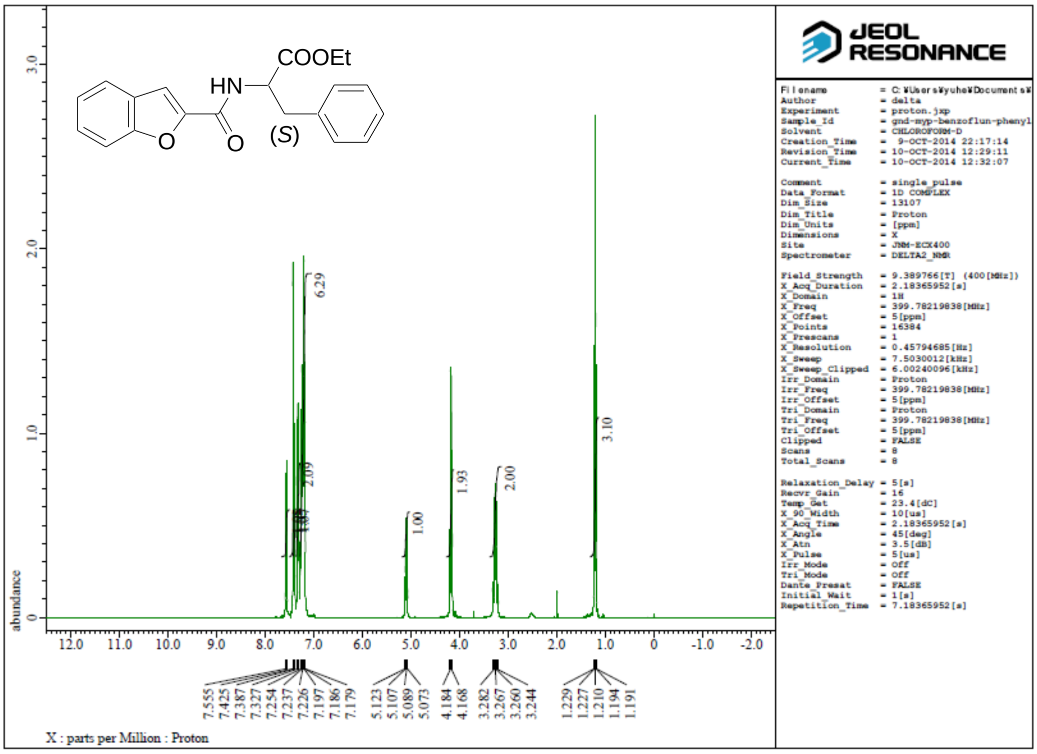

^{1}H NMR spectrum for compound 38

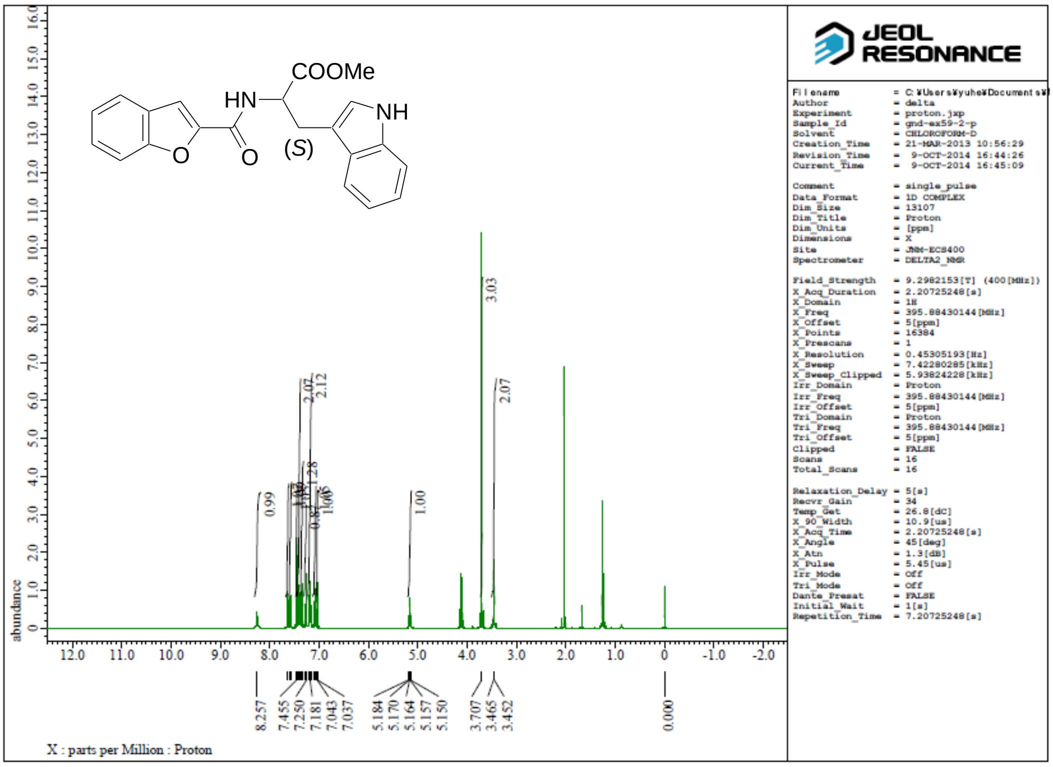

^{1}H NMR spectrum for compound 39

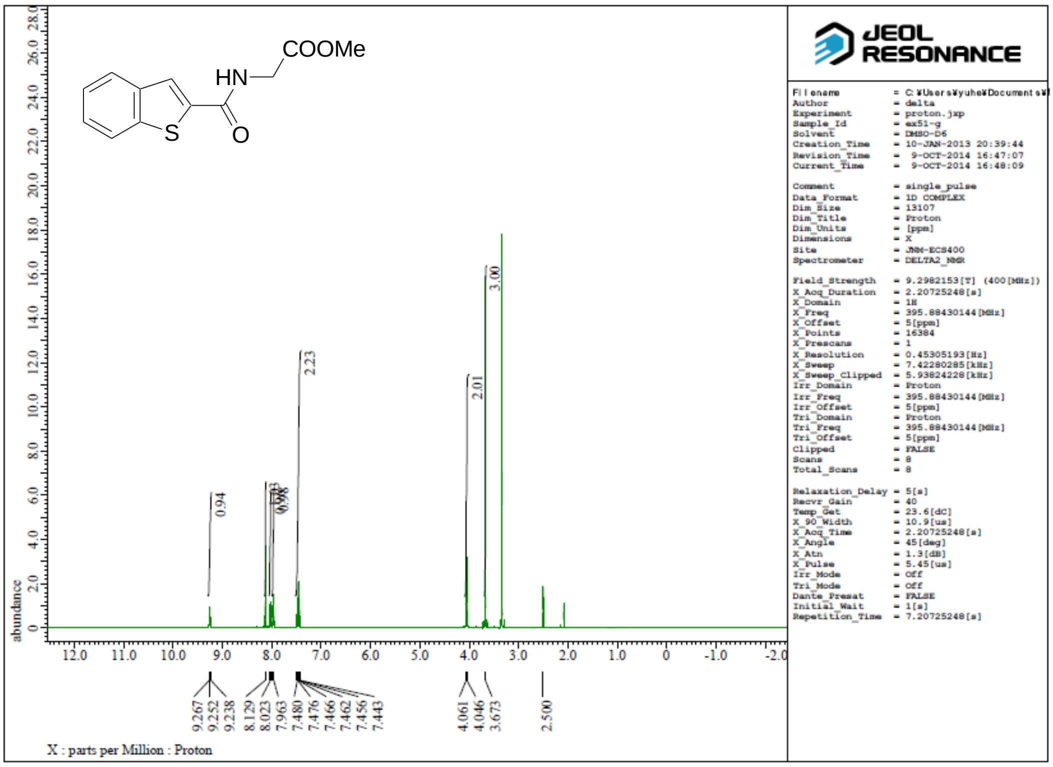

¹H NMR spectrum for compound 40

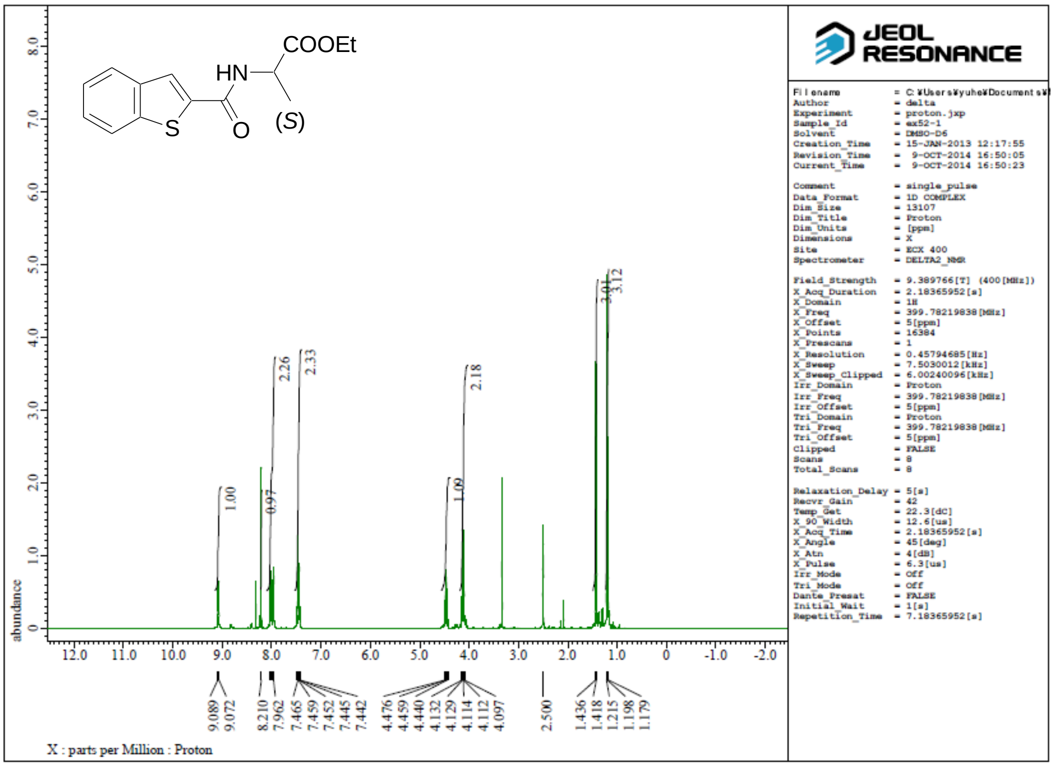

¹H NMR spectrum for compound 41

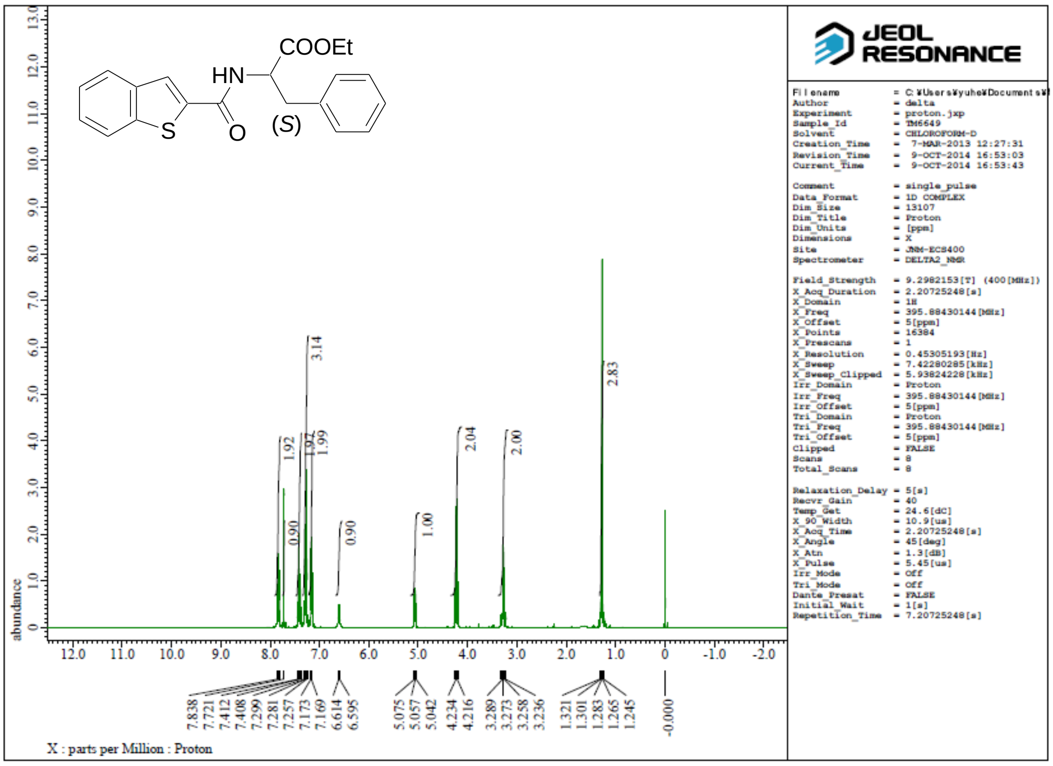

¹H NMR spectrum for compound 42

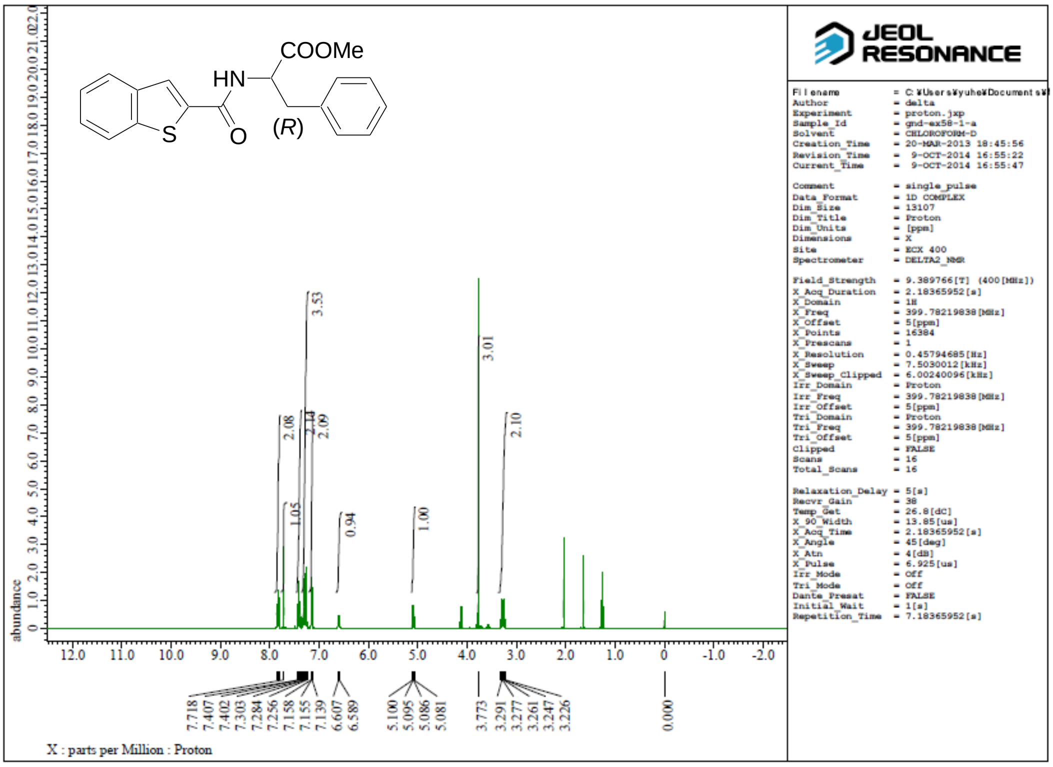

¹H NMR spectrum for compound 43

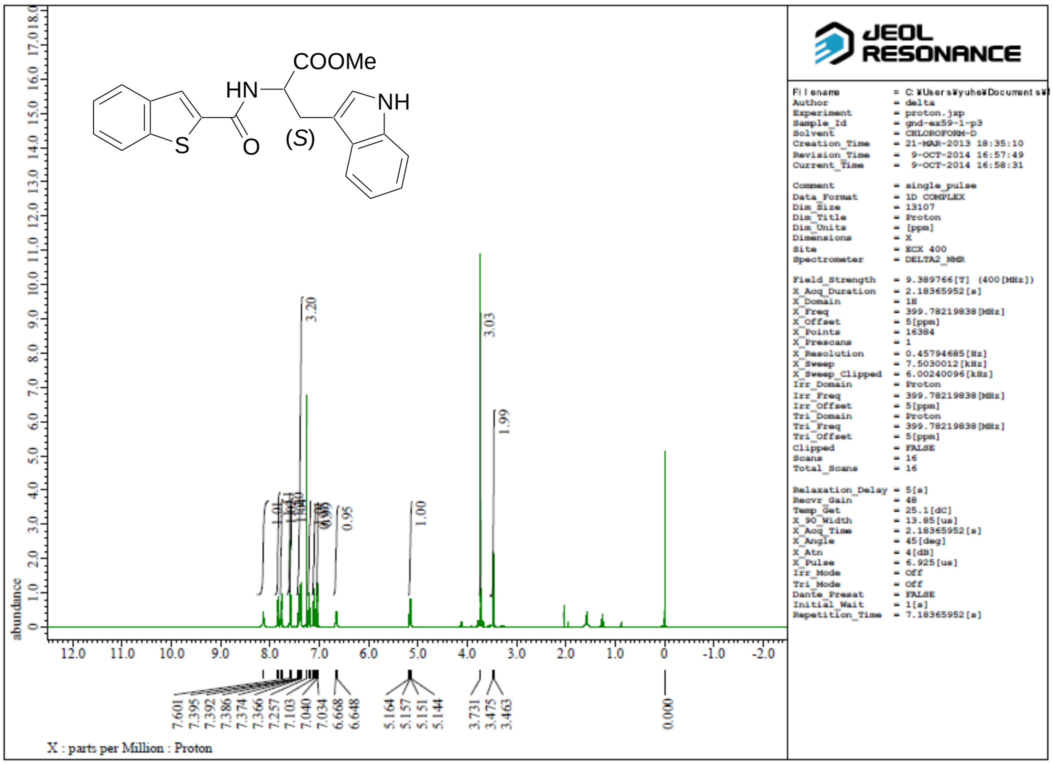

^{1}H NMR spectrum for compound 44

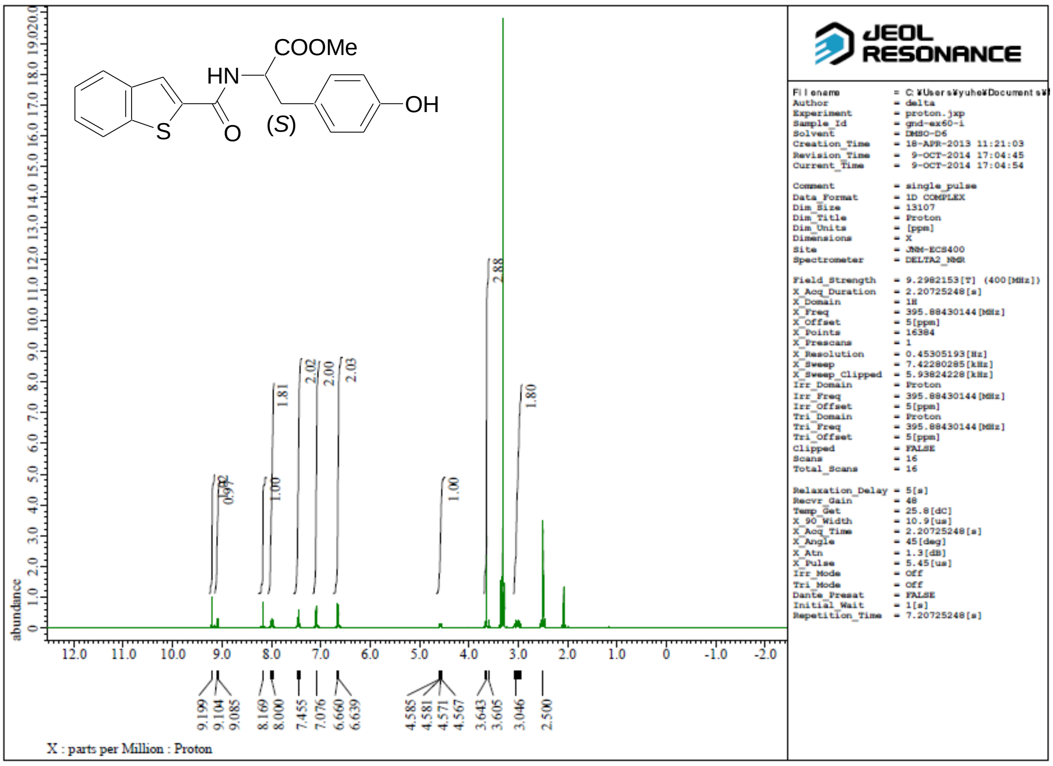

Supplement: Supplementary File 1 [file molecules-23-00885-s001.pdf]
